# Design and Synthesis of New Benzophenone Derivatives with In Vivo Anti-Inflammatory Activity through Dual Inhibition of Edema and Neutrophil Recruitment

**DOI:** 10.3390/molecules23081859

**Published:** 2018-07-26

**Authors:** Jaqueline P. Januario, Thiago B. de Souza, Stefânia N. Lavorato, Tatiane C. S. Maiolini, Olívia S. Domingos, João L. Baldim, Laís R. S. Folquitto, Marisi G. Soares, Daniela A. Chagas-Paula, Danielle F. Dias, Marcelo H. dos Santos

**Affiliations:** 1 Chemistry Institute, Federal University of Alfenas, UNIFAL-MG, Alfenas 37130-001, Minas Gerais, Brazil; jaquelinepj@yahoo.com.br (J.P.J.); tatychryss@gmail.com (T.C.S.M.); oliviadomingos@hotmail.com (O.S.D.); jotaelebaldim@gmail.com (J.L.B.); lais.folquitto@gmail.com (L.R.S.F.); marisigs@gmail.com (M.G.S.); daniapchpa@gmail.com (D.A.C.-P.); daniferdias@gmail.com (D.F.D.); 2 Department of Industrial Pharmacy, Federal University of Santa Maria, UFSM, Santa Maria 97105-900, Rio Grande do Sul, Brazil; thiagobs83@yahoo.com.br; 3 Center of Biological Sciences and Health, Federal University of Western Bahia, UFOB, Barreiras 47808-021, Bahia, Brazil; stelavorato@gmail.com; 4 Department of Chemistry, Federal University of Viçosa, UFV, Viçosa 36570-900, Minas Gerais, Brazil

**Keywords:** hydrazinothiazole, tiosemicarbazone, molecular docking, structure activity relationship, ear edema

## Abstract

A series of novel benzophenone derivatives containing a thiazole heterocyclic nucleus were designed by molecular hybridization. Molecular docking studies have demonstrated the inhibitory potential of the designed compounds against cyclooxygenase (COX) isoenzymes. These compounds were synthesized, characterized, and evaluated for their anti-inflammatory properties by the croton oil-induced ear edema assay to examine their effect on both prostaglandin (PG) production and neutrophils recruitment. The thiazole derivatives displayed a potent effect in terms of reducing ear edema. The analysis suggested that the presence of 4-phenyl-2-hydrazinothiazole and the absence of C4′-OCH_3_ on the benzophenone derivative structure are strongly related to the inhibition of PG production. In addition, the derivatives **2e**, **3a** and **3c** concomitantly inhibit PG production and neutrophil recruitment, which may be a mechanism of action better than of common NSAIDs due to their inability to inhibit the neutrophil recruitment. Thus, these compounds can be considered as potential lead compounds toward the development of new anti-inflammatory drugs with an innovating mechanism of action.

## 1. Introduction

The inflammatory process consists of a physiological response triggered by a lesion or tissue infection and is characterized by a set of symptoms such as pain, edema, and redness in the affected region. After tissue injury, inflammatory chemical mediators lead to increased vascular permeability and chemotaxis to the lesion site [1]. The treatment of inflammation is commonly based on the administration of non-steroidal anti-inflammatory drugs (NSAIDs) [2]. This class of drugs basically acts by inhibiting the isoforms of the enzyme cyclooxygenase: COX-1 and/or COX-2. Prostaglandins (PG) stimulate the production of protective mucus and bicarbonate and also improve blood flow in the gastrointestinal (GI) mucosa. Thus, the inhibition of PG may promote the accumulation and release of hydrogen ions in the stomach mucosa, contributing on GI-related side effects [3]. Conversely, highly selective COX-2 anti-inflammatory drugs are associated with the development of thrombotic events since the selective inhibition of cyclooxygenase isoform 2 inhibits the synthesis of prostacyclin, which is an inhibitor of platelet aggregation. Therefore, the activity of thromboxane produced via COX-1 becomes exacerbated, leading to increased platelet aggregation [2,4,5].

The PG produced by COX-1 or COX-2 are responsible for some symptoms of inflammation including hyperalgesia, edema, and fever. Although the production of PG is inhibited by NSAIDs, other important inflammatory processes are not inhibited. Arachidonic acid is metabolized by the COX or lipoxygenase (LOX) enzymes. If COX is inhibited, the LOX enzymes will continue to produce leukotrienes (LT). LTB_4_ stimulates the chemotaxis of leukocytes such as neutrophils. The gastric side effects of NSAIDs are also attributed to the high neutrophil infiltration, as they might contribute to ulceration by occluding microvessels, reducing mucosal blood flow, and through the release of proteases and free radicals [3]. Thus, compounds which concomitantly inhibit the production of PG and neutrophil recruitment could have fewer associated gastric side effects and could be more effective anti-inflammatory drugs [3,6,7].

Among the NSAIDs, ketoprofen represents one of the most widely used drugs, even though the gastric effects associated with this drug class remain present. Ketoprofen, a benzophenone structure, is available in a variety of forms and is used for treatment in patients with rheumatic diseases such as osteoarthritis, rheumatoid arthritis, inflammatory disorders and pain, proving to be an effective analgesic [8]. New ketoprofen derivatives have been shown to improve activity against the two COX isoforms and are generally more selective for COX-2 [9]. Some derivatives, such as ketoprofenamides containing a heterocyclic nucleus, have exhibited more significant analgesic and anti-inflammatory activities when compared to the original drug [10]. 

Several studies also report a different heterocyclic nucleus with the anti-inflammatory effect [11,12,13,14,15] with the thiazole group being one of them [16,17,18,19,20]. Moreover, the substitution of the carboxylate function of NSAIDs by some azoles groups, thiazole, resulted in an increase in anti-inflammatory activity [21]. The presence of 2-phenyl-thiazole group in meloxicam was shown to be a more active inhibitor in the acute phase than its precursor, the commercial drug used in the treatment of inflammatory diseases containing 2-methylthiazole [22].

**Figure 1:**
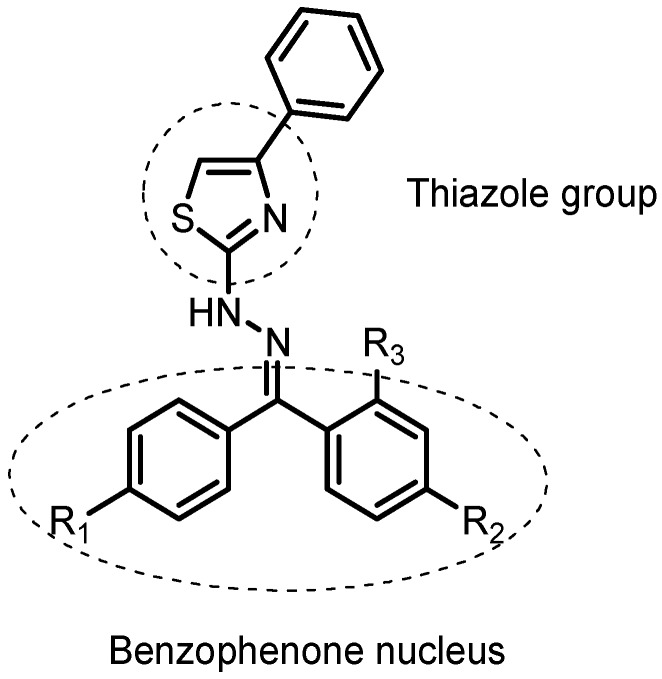
General structure of planned compounds by molecular hybridization.

In our study, we described the synthesis of a novel series of benzophenone derivatives attached to a thiazole group, two known pharmacophores which were potentially anti-inflammatory. The commercially available ketoprofen was used as a reference drug for the structural planning of these proposed compounds (Figure 1 and in Supplementary Material). The new proposed structural pattern can be considered a hybrid between the two pharmacologically known units and may present a higher potential when compared with the isolated prototypes. The planned substances were first analyzed in silico for inhibition capacity of the cyclooxygenase enzymes (COX-1 and COX-2) and considering the promising results, these compounds were then synthesized and theirs activity was evaluated using the ear edema test to analyze their effects on both PG production and neutrophil recruitment. Additionally, we determined the main structural features correlated with anti-inflammatory activity through multivariate statistical analysis.

## 2. Results and Discussion

### 2.1. Docking Studies

There is a widespread use of virtual screening to select potential bioactive compounds, with molecular docking being one of the most used computational approaches for this purpose [23,24]. In order to evaluate the anti-inflammatory potential of the planned compounds, they were docked into the active sites of COX-1 and COX-2 isoenzymes. The docking studies were performed using AutoDock Vina [25] while calculating the predicted binding affinity. For asymmetrical benzophenone derivatives, all regioisomers were evaluated. For comparative purposes, COX isoenzymes inhibitors indomethacin and ketoprofen were also submitted to docking studies. The binding free energies of ligand-enzyme complexes are described in Table 1.

**Figure 2:**
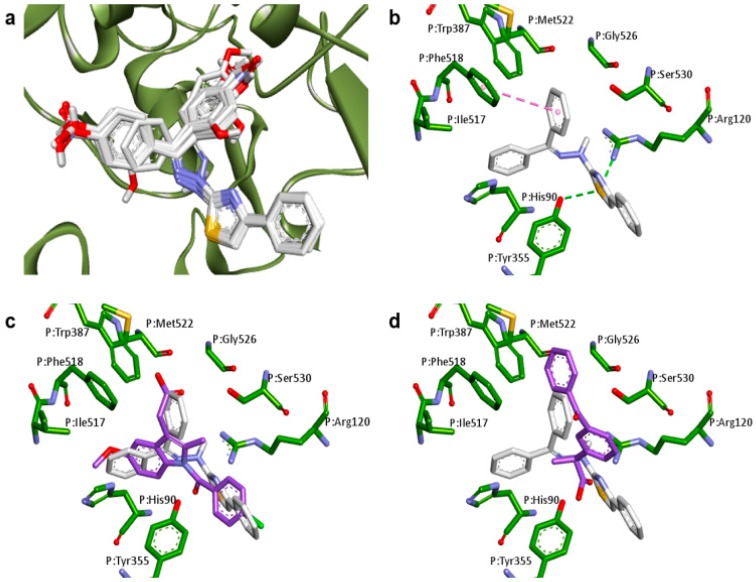
Docking results of planned compounds into COX-1 active site (PDB ID: 2OYU). (**a**) Conformation of planned compounds found in their most stable complex with COX-1; (**b**) Interaction of **3a** (white carbons, yellow sulfurs, blue nitrogens and red oxygens) and COX-1 active site (green carbons). Hydrogen bonds (dotted green line) and pi-pi stacking interactions (dotted pink line) are indicated; (**c**) Conformation comparison between **3a** (white carbons) and indomethacin (purple carbons) interacting with COX-1 (green carbons); (**d**) Conformation comparison between **3a** (white carbons) and ketoprofen (purple carbons) interacting with COX-1 (green carbons).

With the exception of *Z*-isomers of **3e** and **3f**, the best score complexes between the planned thiazole compounds and COX-1 (PDB ID: 2OYU) presented lower predicted binding energies in docking studies than the anti-inflammatory drug indomethacin (Table 1). Moreover, *Z*-isomer of **3g** was able to form a complex with COX-1 that was more stable than the *S*-Ketoprofen-COX-1 complex, indicating that COX-1 is likely to be inhibited by the planned compounds. As shown in Figure 2a, the common scaffold of planned compounds has a similar position in COX-1 active site to individually generate the most stable complex. Compound **3a**, the non-substituted planned benzophenone derivative, was selected to examine the interaction between the compounds in the study (Figure 2b). The two rings of benzophenone moiety of **3a** interaction by van der Waals forces with a hydrophobic pocket of COX-1 active site, formed by the residues HIS90, ILE517, SER530, TRP387, GLY526, and MET522. A pi-pi stacking interaction between one of these rings and the residue PHE518 was observed. Moreover, the sulfur atom of **3a** can act as a hydrogen bond (HB) acceptor, interacting with two residues of COX-1, TYR355, and ARG120.

Docking results indicate that the thiazole derivatives and indomethacin occupy a similar region of the COX-1 active site (Figure 2c). The region where the interaction of benzophenone moiety among planned compounds coincides with that of carboxymethylindole moiety of indomethacin, whereas the interaction region of phenylthiazole moiety coincides with that of benzamide moiety. The sulfur atom of benzophenone derivatives can interact with TYR355 of COX-1 in a similar way as amide carbonyl of indomethacin. Interestingly, the benzophenone moiety of planned compounds does not interact with the COX-1 active site the way benzophenone moiety of ketoprofen does (Figure 2d). On the other hand, this mechanism of interaction allows the thiazole ring to interact with TYR355 and ARG120 by hydrogen bonds similar to the way in which carboxylic group of ketoprofen interacts.

Although the interactions shown in Figure 2a are common among thiazole compounds, the differences in binding energies are an indication that the aromatic substituents can modulate the affinity of these compounds and the enzymes.

In docking studies of the planned compounds into COX-2 (PDB ID: 3NT1), the best score complexes presented lower predicted binding energies than indomethacin-COX-2 complex (Table 1). Despite the low value of binding energy of ketoprofen-COX-2 complex, the *E*-isomer of **3j** could bind to COX-2 with a lower energy, indicating that the planned compounds have the potential to inhibit both COX isoenzymes. In general, the predicted binding energy of a complex between a thiazole derivative and COX-1 was closer to that of the complex between the same compound and COX-2, suggesting that the planned compounds would inhibit the enzymes in a non-selective form which was similar to ketoprofen.

**Figure 3:**
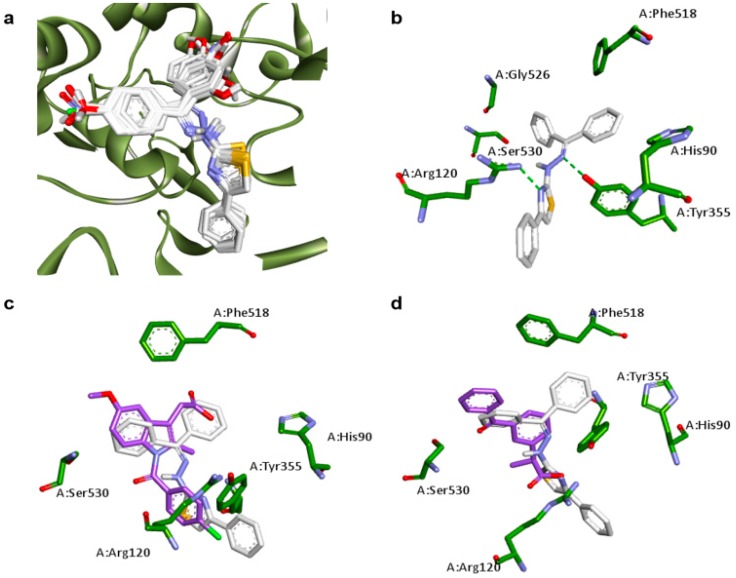
Docking results of planned compounds into COX-2 active site (PDB ID: 3NT1). (**a**) Conformation of planned compounds found in their most stable complex with COX-1; (**b**) Interaction of **3a** (white carbons, yellow sulfurs, blue nitrogens and red oxygens) and COX-2 active site (green carbons). Hydrogen bonds (dotted green line) are indicated; (**c**) Conformation comparisons between **3a** (white carbons) and indomethacin (purple carbons) interacting with COX-2 (green carbons); (**d**) Conformation comparisons between **3a** (white carbons) and ketoprofen (purple carbons) interacting with COX-2 (green carbons).

As observed for COX-1, the common scaffold of each planned compound presented similar coordinates in its most stable complex with COX-2 (Figure 3a). The two rings of benzophenone moiety also find a hydrophobic region to dock into the active site of COX-2, which are included the residues HIS90, PHE518, SER530 and GLY526. In this case, the HB acceptors that interact with the guanidine group of ARG120 are the nitrogen of thiazole ring, instead of the sulfur. The other sp^2^ nitrogen acts as a HB acceptor, which interacts with the OH group of TYR355 (Figure 3b).

All benzophenone derivatives presented lower binding energies than indomethacin when their interaction with COX-2 was evaluated. Docking results reveal that the benzamide ring of indomethacin occupies the same position of thiazole ring of planned compounds (Figure 3c), which impair the interaction of amide carbonyl group with residues ARG120 and TYR355, as observed for the COX-1-indomethacin complex (Figure 2c). Regarding the correlation among ketoprofen and planned compounds when docked into COX-2 active site (Figure 3d), the same observations for COX-1 can be made on COX-2.

**Figure 4:**
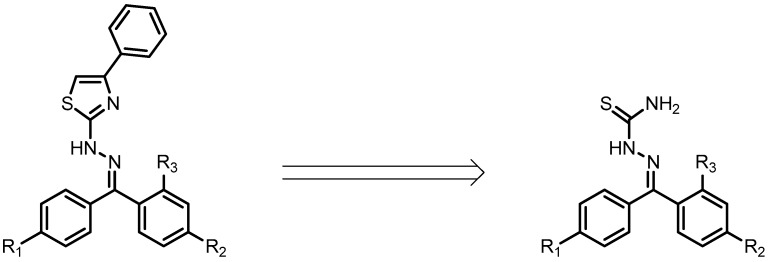
Retrosynthetic analysis of planned thiazole derivatives.

The planned thiazole derivatives are obtained from the respective thiosemicarbazones precursor, as shown in the retrosynthetic analysis in Figure 4. The presence of thiosemicarbazone moiety has already been associated with the anti-inflammatory activity of several compounds [26,27] suggesting that these starting materials could also present an anti-inflammatory potential. Thus, we decided to include these molecules in the docking analysis. The predicted binding energies for the best score complexes between thiosemicarbazones and COX isoenzymes are shown in Table 2.

The predicted binding energies of thiosemicarbazones-COX-1 complexes (Table 2) were similar to those presented by thiazoles-COX-1 complexes (Table 1). However, the binding energies of thiosemicarbazones-COX-2 complexes were higher than the ones presented by thiazoles-COX-2 complexes, suggesting thiazoles may have a greater anti-inflammatory potential, since they can form lower energy complexes with both isoenzymes.

**Figure 5:**
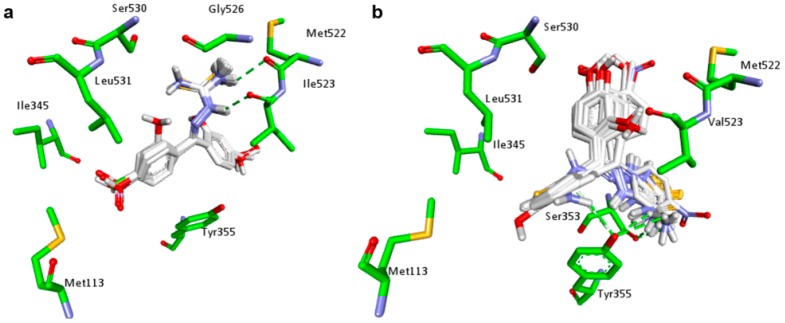
Docking results of **2a**–**2j** isomers into COX isoenzymes active sites. (**a**) Conformation of cited compounds (white carbons, yellow sulfurs, blue nitrogens and red oxygens) found in their most stable complex with COX-1 (PDB ID: 2OYU) (green carbons); (**b**) Conformation of cited compounds found in their most stable complex with COX-2 (PDB ID: 3NT1). Hydrogen bonds (dotted green line) are indicated.

Docking studies indicated that the thiosemicarbazone moiety appears to be important in the interaction between series **2** compounds and COX-1, mediating two hydrogen bonds with MET522 and ILE523 residues in the enzyme active site, as shown in Figure 5a. The predicted binding modes of **2a**–**2j** into the COX-2 active site also suggest the importance of the thiosemicarbazone moiety in the interaction, while only one hydrogen bond is observed, which involves the terminal NH_2_ of thiosemicarbazones and SER353 or TYR355 residue (Figure 5b).

**Figure 6:**
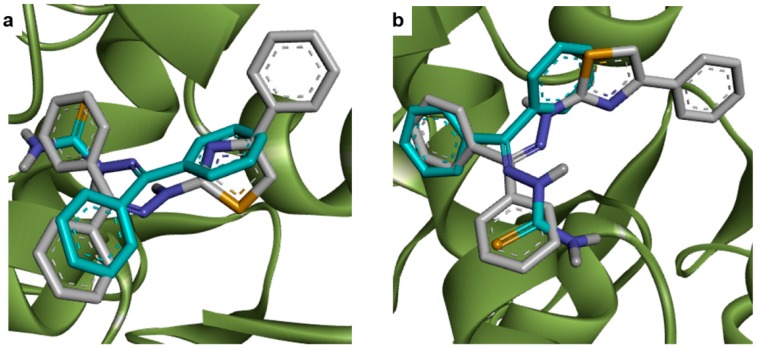
Comparative analysis of predicted poses of thiosemicarbazones and thiazoles into COX isoenzymes active sites (green ribbon). (**a**) Comparison between of **2a** (cyan carbons, yellow sulfurs, blue nitrogens and red oxygens) and **3a** (white carbons) poses into COX-1 (PDB ID: 2OYU) active site; (**b**) Comparison between of **2a** (cyan carbons) and **3a** (white carbons) poses into COX-2 active site (PDB ID: 3NT1).

**Figure 7:**
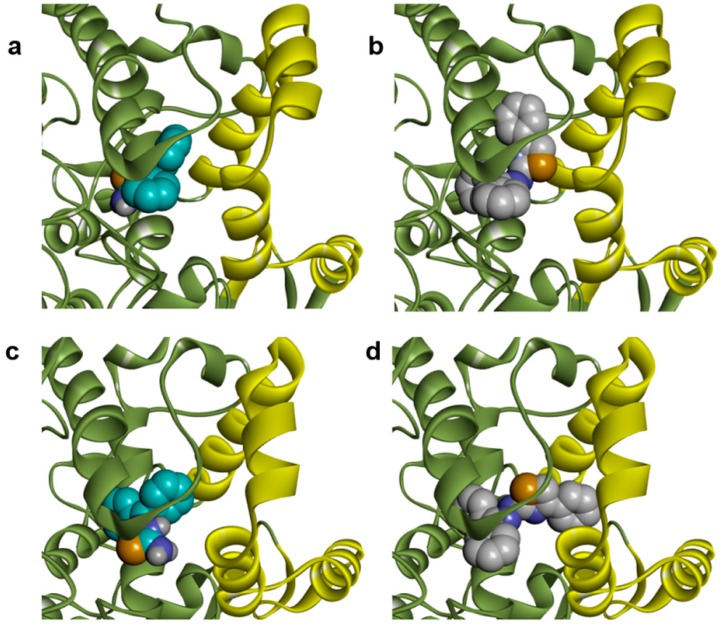
Conformations of **2a** (cyan carbons, yellow sulfur, blue nitrogen) and **3a** (white carbons) into COX isoenzymes active sites, highlighting their position relative to the catalytic (green ribbon) and the membrane binding domain (yellow ribbon). (**a**) Pose of **2a** into COX-1 (PDB ID: 2OYU) active site; (**b**) Pose of **3a** into COX-1 (PDB ID: 2OYU) active site; (**c**) Pose of **2a** into COX-2 (PDB ID: 3NT1) active site; (**d**) Pose of **3a** into COX-2 (PDB ID: 3NT1) active site.

The COX active site is organized as a long hydrophobic channel which begins at the base of the membrane binding domain and extends into the catalytic domain [28]. In docking studies, thiosemicarbazones and thiazoles find similar interaction regions in the active sites of COX isoenzymes, as demonstrated in Figure 6a,b for compounds **2a** and **3a**, which were selected as representatives of each class. Nevertheless, we observed differences in predicted binding energies when we evaluated these compounds against COX-2. Series **3** compounds are larger than series **2** compounds. The volume differences do not seem to interfere substantially with the interaction between these two series with the COX-1 active site, as demonstrated by similar predicted binding energies in Table 1 and Table 2 and by the binding modes shown in Figure 7b. However, docking studies indicate that thiazole compounds may bind in the COX-2 active site fitting the phenyl ring of phenylthiazole moiety with the entrance of the active site, unlike the thiosemicarbazones which do not reach this region (Figure 7c,d). This feature could explain the differences in predicted binding energies observed in docking studies for series **3** and series **2** compounds with COX-2 active site.

### 2.2. Chemistry

**Scheme 1:**
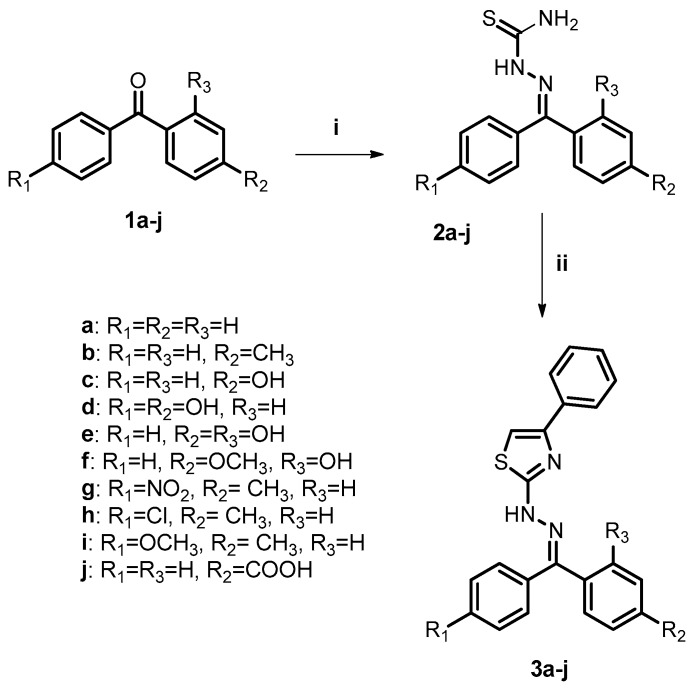
General synthesis of benzophenone thiazole derivatives. Reagents and conditions: (i) thiosemicarbazide (TSC), TsOH, MeOH, 70 °C, 10 h; (ii) 2-bromoacetophenone, i-PrOH, r.t., 4 h.

Considering the promising results obtained from the docking studies, all planned compounds were selected to be synthesized for further biological analysis. The synthesis of benzophenone thiazole derivatives (**3a**–**j**) is shown in Scheme 1 as a result of the reaction between benzophenones (**1a**–**j**) and thiosemicarbazide, followed by a cyclization reaction of thiosemicarbazones (**2a**–**j**) obtained with 2-bromoacetophenone in the presence of isopropyl alcohol, affording these products average yields of 68–92%. The synthesized derivatives have different groups and a heterocyclic ring, as observed in the chemical structure of several anti-inflammatory drugs, such as indomethacin, etoricoxib, and meloxicam. All the thiosemicarbazones and thiazole derivatives were properly characterized by IR, ^1^H and ^13^C nuclear magnetic resonance and high resolution mass spectroscopy (Supplementary Material). It was found that the asymmetrical compounds were obtained as a mixture of E/Z isomers, according to their ^1^H- and ^13^C-NMR spectra. Imines are well-known for their trend to isomerize in solution via catalyzed and non-catalyzed reactions [29]. Analyses of the ^1^H-NMR spectra of the synthesized thiazoles showed signals between 7.32 ppm and 7.59 ppm, corresponding to the protons of thiazole rings, confirming the formation of this heterocyclic core. Signals related to the imine carbons of the compounds were registered between 137.66 ppm and 161.57 ppm in the ^13^C-NMR spectra. In the infrared spectra of derivatives, bands corresponding to the NH bond deformation (3232–3414 cm^−1^) of thiazoles were observed.

**Scheme 2:**
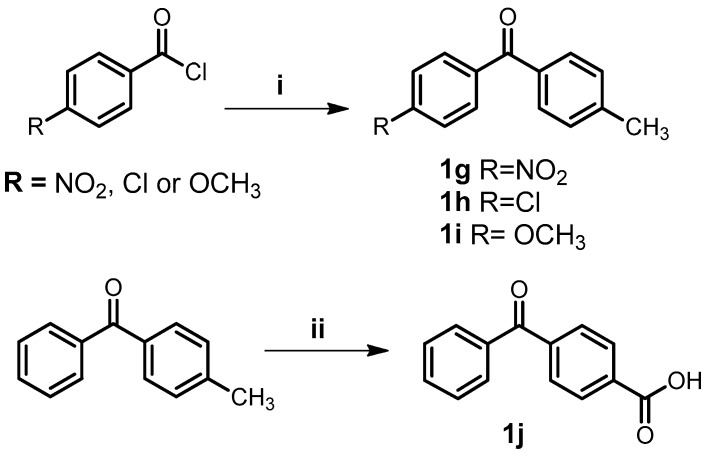
General synthesis of benzophenone derivatives. Reagents and conditions: (i) toluene, AlCl_3_, CH_2_Cl_2_, r.t., 4 h; (ii) CrO_3_, AcOH, H_2_SO_4_, 100 °C, 6 h.

The benzophenones **1g**–**i** were obtained from Friedel-Crafts acylation [30] of toluene with the appropriate acyl chloride in presence of dry aluminum chloride, as shown in Scheme 2. The benzophenone **1j** was obtained by the oxidation of 4-methylbenzophenone with chromium oxide.

### 2.3. Biological Assays

#### 2.3.1. Croton Oil-Induced Ear Edema

An ear edema model was performed with all the derivatives to evaluate the topical inflammatory activity [31,32]. All derivatives were evaluated in vivo because they showed promising docking results and were planned to improve activity. Also, the croton oil-induced ear edema method is an in vivo model that can be used to evaluate both the inhibition of COX pathway (which is mainly responsible for the production of PGE2 and consequently the edema effect) and neutrophil recruitment in the same experiment [33,34,35]. In vivo experiments can evaluate anti-inflammatory activity with regard to the unknown targets and innovative mechanisms, while also evaluating pharmacokinetics and pharmacodynamics of the derivatives planned with different substituents [36,37,38].

In this test, all the thiazole derivatives of benzophenone (**3a**–**3j**) led to the significant inhibition of edema development (Table 3). They showed significant inhibition of edema which was considered statistically similar to the positive control ketoprofen and different from vehicle (negative control) by one-way ANOVA followed by Dunnett’s test. Among them, **3e**, **3f**, **3h** and **3j** displayed a 72% higher reduction in edema, which was close to the values found for the NSAIDs used as positive controls (indomethacin 71% and ketoprofen 68%), reinforcing the results for **3j** which presented low energy to generate a more stable complex in the molecular coupling with the COX enzymes. This finding is relevant since all substances and controls were evaluated at the same concentration of 1.26 μmol/ear (Table 3). Thus, they have similar potency in comparison to positive standards.

A high degree of ear edema inhibition was also observed for intermediate derivatives **2e**–**h**, while excluding **2c** and **2i**. It should be noted that many of the active derivatives are made up of hydroxyl, chloro, nitro and carboxylic acid, and these groups are also present in some NSAIDs such as ketoprofen, diclofenac, indomethacin, meclofenamic acid, nimesulide, and etoricoxib (selective COX-2 inhibitor) [39].

In general, thiazole derivatives inhibited the ear edema at a higher degree than thiosemicarbazones starting materials, in accordance with docking results, suggesting that these two series could act as anti-inflammatory compounds by inhibiting these enzymes. Docking studies predicted a similar inhibitory profile for series **2** and **3** against COX-1 (Table 1 and Table 2), but the predicted binding energies of thiazoles-COX-2 complexes were higher than the ones presented by thiosemicarbazones-COX-2 complexes, which may explain the lower in vivo anti-inflammatory activity of series **2**. As discussed in the docking studies section, these in vivo differences may also be explained due the larger volume of thiazoles, which allows them to reach the entrance of COX-2 active site and to interact with a larger surface of this region.

Confirming the information predicted by docking studies, compounds **2g** and **2h**, whose complexes with COX-1 presented the lowest predicted binding energies, also presented the highest percentage of ear edema inhibition among series **2** compounds (Table 3). Although predicted binding energies indicate **2g** and **2h** form the most stable complexes with COX-1, the role of aromatic substituent to anti-inflammatory activity is not clear by in the docking analysis.

#### 2.3.2. Recruitment of Neutrophils Determination

The anti-inflammatory effects of corticosteroids may inhibit neutrophil recruitment [34]. Thus, a corticosteroid, dexamethasone, was used in this study as a positive control for the inhibition of neutrophil migration measured by the myeloperoxidase activity on ear fragments obtained from mice subjected to croton oil-induced ear edema. Dexamethasone inhibited neutrophil migration by 53.6%. The NSAIDs ketoprofen and indomethacin did not present significant inhibition of neutrophil migration compared to the negative control as expected [34].

We observed in this study that compounds **3a**, **3c** and **2e**, when applied topically, inhibited edema and neutrophil migration (Scheme 1 and Table 3). The heterocyclic derivatives **3a** and **3c** showed 68% and 66% inhibition of neutrophil migration, respectively; **2e** showed 52% migration. It is important to note that **3a** and **3c** exhibited a higher percentage of inhibited neutrophil migration which was inclusive from the reference drug dexamethasone (54%). The NSAIDs ketoprofen and indomethacin did not present significant inhibition of neutrophil migration compared to the negative control, as expected [34].

These derivatives may act similarly to the NSAIDs through inhibition of COX or affect other enzymes which are also responsible for the synthesis of PGE_2_, since they are anti-edematogenic (Table 3). In addition, they also inhibit neutrophil migration, an effect not seen with NSAIDs. Thus, these compounds should be more efficient and free of the main side effects of NSAIDs (gastric lesions), which are associated with neutrophil infiltration [3,32,34]. Therefore, these three derivatives can be considered promising lead compounds toward the development of a more efficient NSAID which presents less gastric side effects.

### 2.4. Important Molecular Features for the Anti-Inflammatory Activity of Designed Derivatives

The multivariate statistical analysis of all benzophenones was carried out with the development of supervised orthogonal projections to the latent structures discriminate analysis (O2PLS-DA) method. This method of analysis reduces the complexity of data by associating *X* to *Y* variables [40]. The multivariate statistical analysis used derivatives from our in-house database for the identification of essential groups for the anti-inflammatory activity. All benzophenone used in this study populated the dataset for this purpose. The analysis also made it possible to find the molecular features that distinguish the designed benzophenones according their activity.

The moieties that reached higher values of variable importance for the projection (VIPs > 1) [41] were 4-phenyl-2-hydrazinothiazole (VIP value: 1.93), C4-CH_3_ (VIP value: 1.74), and carbonyl (VIP value: 1.56). These values indicate that these subunits were important for the separation of instances in the model. The coefficient values indicate that the presence of 4-phenyl-2-hydrazinothiazole (Coeff: 0.30) and the absence of C4′-OCH_3_ (Coeff: −0.34) is a strong indication that the heterocyclic ring is the pharmacophore of this series. Comparing coefficient values for the inactive group (G5 (ns)) the subunit 4-phenyl-2-hydrazinothiazole (Coeff: −0.20) and the presence of a C4′-OCH_3_ (Coeff: 0.42) moiety corroborates this conclusion. It can be clearly observed in the compound **3j** (with a 4-phenyl-2-hydrazinothiazole without C4′-OCH_3_) classified as G1 (****), and the compound **1i** (with a benzophenone core and C4′-OCH_3_, without 4-phenyl-2-hydrazinothiazole), which is inactive. Thus, the substitution pattern of benzophenone, as the presence of 4-phenyl-2-hydrazinothiazole and the absence of C4′-OCH_3_, is important for anti-edematogenic activity.

The distribution of instances and attributes in the loading scatter plot also corroborated these findings as the most correlated attribute (*X* variable) to G1 (****) (*Y* variable) was 4-phenyl-2-hydrazinothiazole, whereas the closest attribute to G5 (ns) is C4′-OCH_3_. This investigation was validated through machine learning while selecting the most important attributes for the benzophenone series with the software Weka 3.8.0 (The University of Waikato, Hamilton, New Zealand). The results showed that after an attribute selection with tenfold cross validation, the heterocyclic substituent was selected as an important attribute for the series.

**Figure 8:**
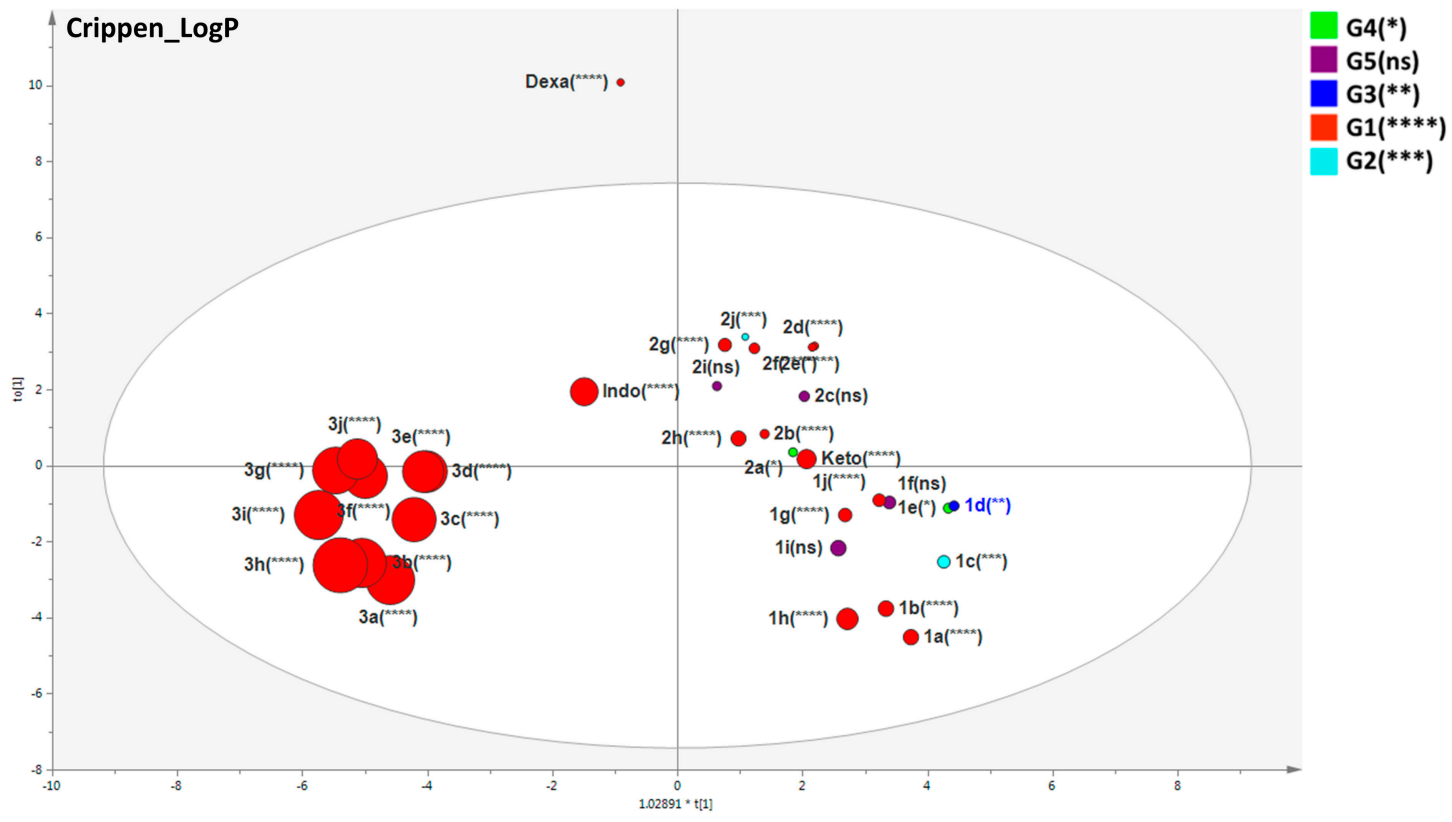
Scatter plot of the supervised O2PLS-DA model using twenty-eight selected descriptors (calculated on PaDEL v.2.2.1 software) explaining the molecular features correlated to the benzophenone derivatives. The size of each variable was defined in the graph properties as being directly correlated to the CrippenLogP to evaluate the values of this feature among all substances. The compounds used as standards in the anti-inflammatory assay (dexamethasone, indomethacin, and ketoprofen) were also evaluated in this model. The asterisks denote the levels of significance in comparison with vehicle (*n* = 8). G1 (**** *p* ≤ 0.0001); G2 (*** *p* ≤ 0.001); G3 (** *p* ≤ 0.01); G4 (* *p* ≤ 0.05), ns = not significant.

With the purpose of investigating molecular characteristics of designed benzophenones compared with their intermediates and standard drugs, a list of biologically relevant molecular descriptors was selected based on the number of aromatic bonds, LogP, electron-richness of the molecule, complexity, lipoaffinity index, number of hydrogen donors and acceptors, first potential of ionization, among others. The values of these molecular descriptors were calculated in the software PaDEL (Version 2.2.1, National University of Singapore, Singapore) and the results were submitted to SIMCA-P with an O2PLS-DA model for measuring their statistical weights and distribution. The designed benzophenones **3a**–**j** was clustered in the score scatter plot evidencing common chemical features which are different from the others (Figure 8).

Molecular descriptors indicated as most important attributes for anti-edematogenic activity (VIPs > 1, Table 4) are correlated with nAromBond (aromatic bonds count), nRotB (rotatable bonds count), lipoaffinity index, nRing (number of rings), Spe (Sum of atomic Pauling electronegativities, scaled on carbon atom), CrippenLogP (atom-based calculation of partition coefficient–log P), Sp (Sum of atomic polarizabilities, scaled on carbon atom), VABC (Van der Waals volume calculated, and apol (Sum of the atomic polarizabilities, including implicit hydrogens). These descriptors also presented coefficient values which were positive to active substances (G1) and negative to the inactive derivatives (G5).

**Figure 9:**
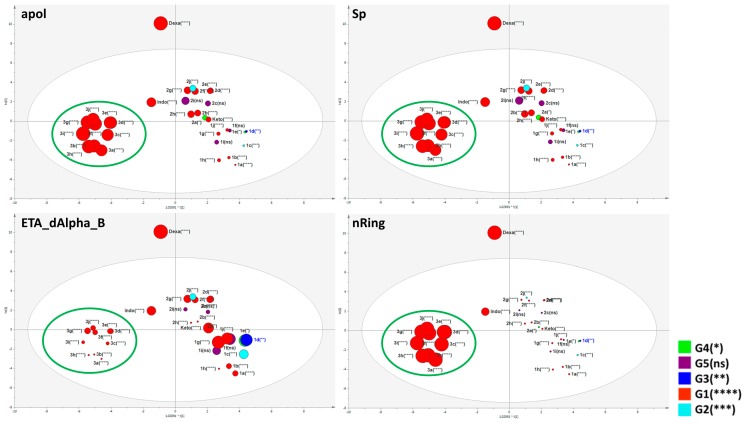
Score scatter plot overview for the supervised O2PLS-DA model with the twenty-eight selected PaDEL descriptors explaining the molecular features correlated to the benzophenone derivatives. The compounds used as standards in the anti-inflammatory assay (dexamethasone, indomethacin, and ketoprofen) were also evaluated. The G1 (****) grouping (highlighted in big green circles clustered to the left of the Hotelling’s t2 ellipse) represents the most similar benzophenone derivatives (in terms of structure, activity, and descriptions). Each score scatter plot demonstrates the variables correlated to a singular molecular feature, presenting different sizes to the respective values in a mode directly proportional to the respective descriptor value. Due to the structural differences between benzophenone derivatives, indomethacin, ketoprofen and dexamethasone, the compound dexamethasone is considered an outlier (as expected) since it has different chemical properties. The asterisks denote the levels of significance in comparison with vehicle (*n* = 8). G1 (**** *p* ≤ 0.0001); G2 (*** *p* ≤ 0.001); G3 (** *p* ≤ 0.01); G4 (* *p* ≤ 0.05), ns = not significant.

The distribution of benzophenones in the score scatter plot based on the biologically relevant descriptors resulted in two clusters: one closer to ketoprofen, suggesting similar molecular features, and the other cluster **3a**–**j** which was closer to indomethacin. The control dexamethasone (a steroidal anti-inflammatory) was an outlier placed outside of the Hotteling’s t2 ellipse due to its structure. This was an important expected result for a good descriptor model, since dexamethasone is quite different from benzophenone derivatives. Figure 9 exhibits one molecular characteristic based on CrippenLogP values. The designed benzophenones **3a**–**j** presented higher CrippenLogP values whereas the other compounds, including ketoprofen, presented smaller values. Higher values of lipoaffinity are in accordance with docking results, since the benzophenones present similar descriptions of indomethacin and the interaction of sulfur atoms of compounds **3a**–**j** with TYR355 is similar to the amide carbonyl of indomethacin in the model of COX-1. As described above, the interaction of designed benzophenones occurred in a hydrophobic pocket of COX-1 and COX-2 active sites. In addition, lipoaffinity, electropological state, the number of rings, electronegatives, polarizabilities, and volume reached higher VIPs correlated to the anti-edematogenic activity corroborating results previously discussed, in which the presence of 4-phenyl-2-hydrazinothiazole is a strong indicator of the inhibition of PG production. In this context, these physico-chemical features can be useful for the projection of novel compounds aimed at improvements in biological activity based on their interaction enzyme-ligand energies and the results of MSA.

Concerning the ability to concomitantly inhibit both edema and neutrophil recruitment, it was not possible to conduct a statistical analysis of molecular features associated with this mechanism of few derivatives which displayed the dual ability. It appears that that the thiazol or thiosemicarbazol group can be important for this mechanism since ketoprofen cannot inhibit neutrophil recruitment and the **2e**, **3a**, and **3c** derivatives have both effects. Also, it appears that all derivatives with bulky substituents missed the inhibition of neutrophil recruitment; thus, these substituents can interfere on this property.

## 3. Materials and Methods

### 3.1. General Information

All synthesized derivatives were chemically characterized by ^1^H and ^13^C nuclear magnetic resonance (NMR) spectra in DMSO-*d*_6_, CD_3_OD, or CDCl_3_ solutions using a BrukerAV-300 spectrometer (Rheinstetten, Germany) at 25 °C and were externally referenced to the tetramethylsilane (TMS) standard. Chemical shifts were reported in ppm (δ) and the coupling constant values (*J*) were given in Hertz. Signal multiplicities are represented as singlet (s), doublet (d), triplet (t), or multiplet (m). The melting points were determined using a PFM-II Aaker apparatus and the high resolution mass spectra were obtained by (UHPLC-UV-DAD-HRMS) using Thermo Scientific Exactive™ equipment powered by Orbitrap™ technology (Thermo Fisher Scientific, Bremen, Germany). Reactions were monitored by thin layer chromatography (TLC) in silica gel which contained a fluorescence indicator and aluminum support (60 G, 0.20 mm thick). Infrared (IR) spectra were obtained with a spectrometer Nicolet iS50 FTIR (Thermo Scientic, Waltham, MA, USA) coupled to GladiATR (Thermo Scientific, Waltham, MA USA). The benzophenones **1a**–**f** and all chemicals were purchased from Sigma Aldrich^®^ (St. Louis, MO, USA).

### 3.2. General Procedure for the Synthesis of Benzophenones *(**1g**–**i**)*

Toluene (5.2 mmol) was added to a solution of appropriate benzoyl chloride (6.2 mmol) in anhydrous dichloromethane (15 mL). The reaction was cooled to 5 °C and added anhydrous aluminum chloride (7.8 mmol) [30]. The reaction was stirred at room temperature for 4 h. The mixture was extracted with water/dichloromethane; the organic layer was washed with saturated NaHCO_3_ solution until pH 6–7, dried over anhydrous magnesium sulfate and evaporated. The crude product was purified by column chromatography. 

*(4-Nitrophenyl)(p-tolyl)methanone* (**1g**) [42]. Yield 50%, yellow, m.p. 221–225 °C. IR-ATR (cm^−1^) 3098, 2971, 2921, 1650, 1595, 1457, 1515, 1349, 830. ^1^H-NMR (300 MHz, CDCl_3_) δ 8.33 (d, 2H, ^3^*J* = 9.0 Hz), 7.92 (d, 2H, ^3^*J* = 9.0 Hz), 7.72 (d, 2H, ^3^*J* = 8.1 Hz), 7.33 (d, 2H, ^3^*J* = 7.8 Hz), 2.47 (s, 3H). ^13^C-NMR (75 MHz, CDCl_3_) δ 194.47, 149.68, 144.57, 143.32, 133.64, 130.54, 130.32, 129.38, 123.48, 21.74.

*(4-Chlorophenyl)(p-tolyl)methanone* (**1h**). Yield 52%, white solid, m.p. 219–221 °C. IR-ATR (cm^−1^) 3030, 2933, 2882, 1641, 1604, 1582, 1480, 851, 744. ^1^H-NMR (300 MHz, CDCl_3_) δ 7.73 (d, 2H, ^3^*J =* 8.8 Hz), 7.68 (d, 2H, ^3^*J =* 8.1 Hz), 7.44 (d, 2H, ^3^*J =* 9.0 Hz), 7.28 (d, 2H, ^3^*J =* 7.8 Hz), 2.44 (s, 3H). ^13^C-NMR (75 MHz, CDCl_3_) δ 195.28, 143.55, 138.60, 136.23, 134.53, 131.35, 130.18, 129.10, 128.56, 21.66. HRMS (ESI) calculated for C_14_H_12_ClO [M + H]^+^ 231.0577, found 231.0571.

*(4-Methoxyphenyl)(p-tolyl)methanone* (**1i**). Yield 38%, white solid, m.p. 89–93 °C. IR-ATR (cm^−1^) 3002, 2981, 2920, 2854, 1641, 1594, 1504, 1445, 1257, 1020, 846. ^1^H-NMR (300 MHz, CDCl_3_) δ 7.74 (d, 2H, ^3^*J =* 9.0 Hz), 7.60 (d, 2H, ^3^*J =* 8.1 Hz), 7.20 (d, 2H, ^3^*J =* 7.2 Hz), 6.88 (d, 2H, ^3^*J =* 9.0 Hz), 3.81 (s, 3H), 2.36 (s, 3H). ^13^C-NMR (75 MHz, CDCl_3_) δ 195.37, 163.04, 142.61, 135.52, 132.43, 130.50, 130.00, 128.87, 113.49, 55.48, 21.61. HRMS (ESI) calculated for C_15_H_15_O_2_ [M + H]^+^ 227.1072, found 227.1065.

### 3.3. Synthesis of 4-Benzoylbenzoic Acid *(**1j**)*

4-methylbenzophenone (**1b**) (10 mmol) and 10.4 mL of glacial acetic acid was stirred at room temperature. The reaction was cooled to 5 °C and chromium (VI) oxide (40 mmol) and 2 mL of sulfuric acid were added. The system was heated at 100 °C for 6 h. After cooling, water was added and the mixture was extracted with ethyl acetate. The organic layer was washed with NaOH 0.1 M, then the alkaline aqueous layer was acidified with HCl and the white solid **1j** was filtered. Yield 76%, m.p. 205–209 °C. IR-ATR (cm^−1^) 3051, 1676, 1650, 1597, 1577, 1498. ^1^H-NMR (300 MHz, DMSO-*d*_6_) δ 8.07 (d, 2H, ^3^*J* = 8.6 Hz), 7.79 (d, 2H, ^3^*J* = 8.6 Hz), 7.74–7.71 (m, 2H), 7.67 (d, 1H, ^3^*J* = 7.4 Hz), 7.56 (d, 2H, ^3^*J* = 7.7 Hz). ^13^C-NMR (300 MHz, DMSO-*d*_6_) δ 195.92, 167.14, 141.00, 136.93, 134.46, 133.61, 130.19, 130.09, 129.87, 129.17 [43]. HRMS (ESI) calculated for C_14_H_9_O_3_ [M − H]^−^ 225.0552, found 225.0553.

### 3.4. General Procedure for the Synthesis of Thiosemicarbazones *(**2a**–**j**)*

Thiosemicarbazide (2.75 mmol) and *p*-toluenesulfonic acid monohydrate catalytic were dissolved in methanol (20 mL) and water (1 mL). The solution was refluxed for 15 min followed by the addition of benzophenone (2.75 mmol) [44]. Then the resulting precipitate was isolated by filtration and recrystallized or purified by column chromatography.

*1-(Diphenylmethylene) thiosemicarbazide* (**2a**)*.* Yield 80%, white solid, m.p. 208–212 °C. IR-ATR (cm^−1^) 3407, 3344, 3232, 3050, 1596, 1466, 1438, 1025. ^1^H-NMR (300 MHz, DMSO-*d*_6_) δ 8.60 (s, 1H), 8.41 (s, 2H), 7.68–7.60 (m, 5H), 7.41–7.31 (m, 5H). ^13^C-NMR (75 MHz, DMSO-*d*_6_) δ 178.29, 149.69, 136.72, 131.63, 130.48, 130.32, 128.84, 128.72, 128.02. HRMS (ESI) calculated for C_14_H_14_N_3_S [M + H]^+^ 256.0908, found 256.0900.

*(E,Z)-1-(Phenyl(p-tolyl)methylene) thiosemicarbazide* (**2b**). Yield 63%, light yellow solid, m.p. 198–203 °C. IR-ATR (cm^−1^) 3414, 3351, 3245, 3050, 1598, 1473, 1076. ^1^H-NMR (300 MHz, DMSO-*d*_6_) δ 8.61 (s, 1H), 8.34 (s, 2H), 7.67–7.62 (m, 2H), 7.54 (d, 1H, ^3^*J =* 8.3 Hz), 7.46 (d, 1H, ^3^*J =* 7.8 Hz), 7.40–7.36 (m, 2H), 7.32 (dd, 1H, ^4^*J =* 1.6 Hz and ^3^*J =* 7.8 Hz), 7.22 (d, 1H, ^3^*J =* 8.0 Hz), 7.18 (d, 1H, ^3^*J =* 8.1 Hz), 2.43 (s, 3H). ^13^C-NMR (75 MHz, DMSO-*d*_6_) δ 178.22, 149.71, 140.12, 136.89, 134.07, 130.86, 130.28, 129.42, 128.65, 128.06, 21.47. HRMS (ESI) calculated for C_15_H_16_N_3_S [M + H]^+^ 270.1065, found 270.1055.

*(E,Z)-1-((4-Hydroxyphenyl)(phenyl)methylene) thiosemicarbazide* (**2c**). Yield 74%, white solid, m.p. 186–191 °C. IR-ATR (cm^−1^) 3498, 3336, 3264, 3040, 1586, 1476, 1415, 1076. ^1^H-NMR (300 MHz, DMSO-*d*_6_) δ 9.92 (s, 1H), 8.48 (s, 1H), 8.23 (s, 2H), 7.64–7.60 (m, 2H), 7.47 (d, 2H, ^3^*J =* 8.6 Hz), 7.31–7.28 (m, 2H), 7.14 (d, 1H, ^3^*J =* 8.4 Hz), 6.74 (d, 2H, ^3^*J =* 8.6 Hz). ^13^C-NMR (75 MHz, DMSO-*d*_6_) δ 177.85, 159.68, 150.13, 137.25, 130.23, 132.01, 129.80, 128.65, 128.16, 115.67. HRMS (ESI) calculated for C_14_H_14_N_3_OS [M + H]^+^ 272.0858, found 272.0850.

*1-(bis(4-Hydroxyphenyl)methylene) thiosemicarbazide* (**2d**). Yield 71%, white solid, m.p. 282–286 °C. IR-ATR (cm^−1^) 3400, 3343, 3276, 2996, 1604, 1486, 1428, 1082. ^1^H-NMR (300 MHz, CD_3_OD) δ 7.49 (d, 2H, ^3^*J =* 8.9 Hz), 7.15 (d, 2H, ^3^*J =* 8.7 Hz), 7.03 (d, 2H, ^3^*J =* 8.8 Hz), 6.78 (d, 2H, ^3^*J =* 9.0 Hz). ^13^C-NMR (75 MHz, CD_3_OD) δ 177.62, 159.36, 158.86, 152.33, 129.83, 129.44, 128.36, 122.31, 116.06, 114.75. HRMS (ESI) calculated for C_14_H_14_N_3_O_2_S [M + H]^+^ 288.0807, found 288.0798.

*(E,Z)-1-((2,4-Dihydroxyphenyl)(phenyl)methylene) thiosemicarbazide* (**2e**). Yield 68%, light yellow solid, m.p. 210–213 °C. IR-ATR (cm^−1^) 3496, 3383, 3227, 3156, 1578, 1486, 1443, 1091. ^1^H-NMR (300 MHz, DMSO-*d*_6_) δ 10.03 (s, 1H), 9.84 (s, 1H), 8.52 (s, 1H), 8.42 (s, 1H), 8.23 (s, 1H), 7.69–7.66 (m, 2H), 7.37–7.33 (m, 3H), 6.83 (d, 1H, ^3^*J =* 8.3 Hz), 6.52 (d, 1H, ^4^*J =* 2.2 Hz), 6.43 (dd, 1H, ^3^*J =* 8.3 Hz and ^4^*J =* 2.2 Hz). ^13^C-NMR (75 MHz, DMSO-*d*_6_) δ 178.05, 160.54, 156.27, 148.55, 137.53, 131.06, 129.78, 128.61, 128.01, 108.87, 108.22, 103.47. HRMS (ESI) calculated for C_14_H_14_N_3_O_2_S [M + H]^+^ 288.0807, found 288.0803.

*(E,Z)-1-((2-Dihydroxy-4-methoxyphenyl)(phenyl)methylene) thiosemicarbazide* (**2f**). Yield 55%, white solid, m.p. 95–99 °C. IR-ATR (cm^−1^) 3407, 3304, 3122, 3060, 2975, 1591, 1487, 1420, 1088. ^1^H-NMR (300 MHz, CD_3_OD) δ 7.66 (d, 2H, ^3^*J =* 7.0 Hz), 7.38–7.34 (m, 3H), 6.96 (d, 1H, ^3^*J =* 7.9 Hz), 6.67–6.64 (m, 2H), 3.87 (s, 3H). ^13^C-NMR (75 MHz, CD_3_OD) δ 179.61, 164.05, 157.40, 151.21, 138.63, 132.04, 130.74, 129.30, 128.89, 111.91, 107.55, 102.91, 55.91. HRMS (ESI) calculated for C_15_H_16_N_3_O_2_S [M + H]^+^ 302.0963, found 302.0961.

*(E,Z)-((4-Nitrophenyl)(p-tolyl)methylene) thiosemicarbazide* (**2g**). Yield 63%, yellow solid, m.p. 244–248 °C. IR-ATR (cm^−1^) 3409, 3352, 3254, 3102, 2920, 1591, 1466, 1406, 1510, 1346, 1069. ^1^H-NMR (300 MHz, DMSO-*d*_6_) δ 8.66 (s, 3H), 8.18 (d, 2H, ^3^*J =* 9.1 Hz), 7.89 (d, 2H, ^3^*J =* 8.8 Hz), 7.48 (d, 2H, ^3^*J =* 7.8 Hz), 7.24 (d, 2H, ^3^*J =* 7.8 Hz), 2.42 (s, 3H). ^13^C-NMR (75 MHz, DMSO-*d*_6_) δ 179.26, 148.80, 148.20, 143.76, 141.32, 131.78, 130.23, 129.65, 129.38, 124.62, 22.16. HRMS (ESI) calculated for C_15_H_13_N_4_O_2_S [M − H]^−^ 313.0759, found 313.0766.

*(E,Z)-1-((4-Chlorophenyl)(p-tolyl)methylene) thiosemicarbazide* (**2h**). Yield 50%, yellow solid, m.p. 198–203 °C. IR-ATR (cm^−1^) 3423, 3342, 3236, 3134, 2915, 1586, 1471, 1081, 721, 686. ^1^H-NMR (300 MHz, DMSO-*d*_6_) δ 8.41 (s, 3H), 7.67 (dd, 2H, ^3^*J =* 8.6 Hz and ^4^*J =* 2.1 Hz), 7.51 (d, 1H, ^3^*J =* 8.2 Hz), 7.46 (d, 1H, ^3^*J =* 8.0 Hz), 7.42 (d, 1H, ^3^*J =* 8.6 Hz), 7.34 (d, 1H, ^3^*J =* 8.4 Hz), 7.22 (d, 1H, ^3^*J =* 8.0 Hz), 7.18 (d, 1H, ^3^*J =* 8.3 Hz), 2.31 (s, 3H). ^13^C-NMR (75 MHz, DMSO-*d*_6_) δ 178.30, 148.51, 140.32, 140.09, 135.84, 134.90, 130.96, 129.73, 128.84, 128.65, 21.47. HRMS (ESI) calculated for C_15_H_15_ClN_3_S [M + H]^+^ 304.0675, found 304.0667.

*(E,Z)-1-((4-Methoxyphenyl)(p-tolyl)methylene) thiosemicarbazide* (**2i**). Yield 80%, white solid, m.p. 171–173 °C. IR-ATR (cm^−1^) 3430, 3346, 3262, 3153, 2917, 1598, 1473, 1246, 1020, 1081. ^1^H-NMR (300 MHz, CDCl_3_) δ 7.46 (d, 1H, ^3^*J =* 9.1 Hz), 7.40 (d, 1H, ^3^*J =* 8.2 Hz), 7.35 (d, 1H, ^3^*J =* 8.4 Hz), 7.20 (d, 1H, ^3^*J =* 8.8 Hz), 7.14 (d, 2H, ^3^*J =* 8.0 Hz), 7.05 (d, 1H, ^3^*J =* 8.9 Hz), 6.85 (d, 1H, ^3^*J =* 9.1 Hz), 3.83 (s, 3H), 2.44 (s, 3H). ^13^C-NMR (75 MHz, CDCl_3_) δ 178.38, 161.38, 151.40, 140.54, 134.10, 130.51, 129.45, 128.39, 123.10, 113.84, 55.41, 21.46. HRMS (ESI) calculated for C_16_H_18_N_3_OS [M + H]^+^ 300.1171, found 300.1173.

*(E,Z)-(4-Phenylthiosemicarbazidomethyl)benzoic acid* (**2j**). Yield 84%, yellow solid, m.p. 209–212 °C. IR-ATR (cm^−1^) 3474, 3359, 3344, 2991, 1586, 1682, 1462, 1430, 1078. ^1^H-NMR (300 MHz, DMSO-*d*_6_) δ 8.52 (s, 3H), 7.91 (d, 2H, ^3^*J =* 8.5 Hz), 7.76 (d, 2H, ^3^*J =* 8.5 Hz), 7.69–7.62 (m, 3H), 7.35 (d, 2H, ^3^*J =* 7.7 Hz). ^13^C-NMR (75 MHz, DMSO-*d*_6_) δ 178.48, 167.43, 148.51, 140.75, 131.87, 131.22, 130.44, 129.73, 129.39, 128.78, 128.03. HRMS (ESI) calculated for C_15_H_14_N_3_O_2_S [M + H]^+^ 300.0807, found 300.0799.

### 3.5. General Procedure for the Synthesis of Thiazole Derivatives *(**3a**–**j**)*

2-bromoacetophenone (0.78 mmol) was added to a solution of corresponding thiosemicarbazone (0.78 mmol) in 6 mL isopropyl alcohol [45]. The reaction mixture was stirred at room temperature for approximately 4 h. The resulting precipitate was isolated by filtration and washed with cold water.

*1-(Diphenylmethylene)-2-(4-phenylthiazol-2-yl)hydrazine* (**3a**). Yield 92%, yellow solid, m.p. 266–271 °C. IR-ATR (cm^−1^) 3080, 2572, 1594, 1489, 1470, 1444, 1072. ^1^H-NMR (300 MHz, DMSO-*d*_6_) δ 7.76 (d, 2H, ^3^*J =* 8.1 Hz), 7.57–7.52 (m, 3H), 7.47–7.43 (m, 2H), 7.39–7.34 (m, 5H), 7.33–7.31 (m, 3H), 7.29–7.25 (m, 1H). ^13^C-NMR (75 MHz, DMSO-*d*_6_) δ 169.49, 137.63, 133.94, 133.12, 129.92, 129.79, 129.17, 128.96, 128.37, 127.28, 126.05, 104.73. HRMS (ESI) calculated for C_22_H_18_N_3_S [M + H]^+^ 356.1221, found 356.1215.

*(E,Z)-1-(Phenyl(p-tolyl)methylene)-2-(4-phenylthiazol-2-yl)hydrazine* (**3b**). Yield 68%, white solid, m.p. 234–238 °C. IR-ATR (cm^−1^) 3412, 3060, 2904, 1597, 1471, 1442, 688. ^1^H-NMR (300 MHz, DMSO-*d*_6_) δ 7.79 (d, 2H, ^3^*J =* 7.0 Hz), 7.59–7.53 (m, 3H), 7.41–7.39 (m, 3H), 7.36–7.30 (m, 5H), 7.21 (d, 2H, ^3^*J =* 8.5 Hz), 2.32 (s, 3H). ^13^C-NMR (75 MHz, DMSO-*d*_6_) δ 169.52, 139.40, 135.02, 134.08, 133.36, 130.33, 129.81, 129.71, 129.54, 129.20, 129.09, 128.79, 128.31, 127.30, 126.06, 104.59. 21.32. HRMS (ESI) calculated for C_23_H_20_N_3_S [M + H]^+^ 370.1378, found 370.1371.

*(E,Z)-1-((4-Hydroxyphenyl)(phenyl)methylene)-2-(4-phenylthiazol-2-yl)hydrazine* (**3c**). Yield 89%, orange solid, m.p. 249–254 °C. IR-ATR (cm^−1^) 3050, 1604, 1466, 1438. ^1^H-NMR (300 MHz, DMSO-*d*_6_) δ 7.77 (t, 2H, ^3^*J =* 7.2 Hz), 7.59–7.50 (m, 3H), 7.42–7.37 (m, 3H), 7.34–7.31 (m, 4H), 7.17 (d, 1H, ^3^*J =* 8.5 Hz), 6.97 (d, 1H, ^3^*J =* 8.4 Hz), 6.79 (d, 1H, ^3^*J =* 8.7 Hz). ^13^C-NMR(75 MHz, DMSO-*d*_6_) δ 169.38, 159.41, 151.90, 147.86, 137.98, 133.25, 130.76, 129.75, 129.15, 128.89, 128.56, 127.57, 126.11, 123.12, 115.81, 104.55. HRMS (ESI) calculated for C_22_H_16_N_3_OS [M − H]^−^ 370.1014, found 370.1015.

*1-(bis(4-Hydroxyphenyl)methylene)-2-(4-phenylthiazol-2-yl)hydrazine* (**3d**). Yield 86%, light pink solid, m.p. 286–291 °C. IR-ATR (cm^−1^) 3307, 3245, 3068, 1608, 1507, 1433, 689. ^1^H-NMR (300 MHz, DMSO-*d*_6_) δ 7.78 (d, 2H, ^3^*J =* 7.1 Hz), 7.44–7.39 (m, 2H), 7.37–7.33 (m, 3H), 7.34 (s, 1H), 7.16 (d, 2H, ^3^*J =* 8.6 Hz), 6.96 (d, 2H, ^3^*J =* 8.6 Hz), 6.79 (d, 2H, ^3^*J =* 8.8 Hz). ^13^C-NMR (75 MHz, DMSO-*d*_6_) δ 169.18, 159.43, 158.85, 152.58, 147.64, 133.27, 130.72, 129.39, 129.17, 128.75, 128.61, 126.17, 123.32, 116.49, 115.73, 104.58. HRMS (ESI) calculated for C_22_H_16_N_3_O_2_S [M − H]^−^ 386.0963, found 386.0967.

*(E,Z)-1-((2,4-Dihydroxyphenyl)(phenyl)methylene)-2-(4-phenylthiazol-2-yl)hydrazine* (**3e**). Yield 73%, yellow solid, m.p. > 300 °C. IR-ATR (cm^−1^) 3380, 3230, 3044, 1597, 1512, 1443, 681. ^1^H-NMR (300 MHz, DMSO-*d*_6_) δ 7.78 (d, 2H, ^3^*J =* 7.2 Hz), 7.53–7.51 (m, 2H), 7.44–7.37 (m, 5H), 7.36 (s, 1H), 7.33–7.30 (m, 1H), 6.87 (d, 1H, ^3^*J =* 8.3 Hz), 6.52 (d, 1H, ^4^*J =* 2.2 Hz), 6.42 (dd, 1H, ^3^*J =* 8.3 Hz and ^4^*J =* 2.2 Hz). ^13^C-NMR (75 MHz, DMSO-*d*_6_) δ 168.92, 160.39, 156.54, 137.84, 133.29, 131.19, 129.60, 129.19, 128.93, 128.80, 128.66, 127.32, 126.17, 125.94, 110.02, 108.03, 104.91, 103.62. HRMS (ESI) calculated for C_22_H_16_N_3_O_2_S [M − H]^−^ 386.0963, found 386.0968.

*(E,Z)-1-((2-Dihydroxy-4-methoxyphenyl)(phenyl)methylene)-2-(4-phenylthiazol-2-yl)hydrazine* (**3f**). Yield 68%, orange solid, m.p. 254–256 °C. IR-ATR (cm^−1^) 3413, 3117, 3049, 2951, 1596, 1492, 1442, 696. ^1^H-NMR (300 MHz, DMSO-*d*_6_) δ 7.79 (d, 2H, *J =* 7.1 Hz), 7.54–7.51 (m, 2H), 7.44–7.32 (m, 6H), 7.37 (s, 1H), 7.00 (d, 1H, ^3^*J =* 8.2 Hz), 6.61–6.57 (m, 2H), 3.80 (s, 3H). ^13^C-NMR (75 MHz, DMSO-*d*_6_) δ 169.01, 161.96, 156.68, 149.41, 148.29, 137.78, 133.54, 131.29, 129.54, 129.16, 128.82, 128.55, 127.20, 126.18, 111.91, 106.27, 104.88, 102.37, 55.60. HRMS (ESI) calculated for C_23_H_20_N_3_O_2_S [M + H]^+^ 402.1276, found 402.1270.

*(E,Z)-1-((4-Nitrophenyl)(p-tolyl)methylene)-2-(4-phenylthiazol-2-yl)hydrazine* (**3g**). Yield 83%, orange solid, m.p. 212–217 °C. IR-ATR (cm^−1^) 3023, 2911, 1600, 1511, 1481, 1443, 1339, 696. ^1^H-NMR (300 MHz, DMSO-*d*_6_) δ 8.37 (d, 1H, ^3^*J =* 8.7 Hz), 8.21 (d, 1H, ^3^*J =* 8.6 Hz), 7.79 (d, 2H, ^3^*J =* 7.2 Hz), 7.65 (d, 2H, ^3^*J =* 8.8 Hz), 7.38–7.36 (m, 2H), 7.34–7.32 (m, 2H), 7.23–7.18 (m, 3H), 2.40 (s, 3H). ^13^C-NMR (75 MHz, DMSO-*d*_6_) δ 169.25, 148.37, 147.49, 144.16, 139.58, 134.51, 131.11, 130.53, 129.67, 129.16, 129.08, 127.81, 126.91, 125.94, 124.20, 104.98, 21.50. HRMS (ESI) calculated for C_23_H_17_N_4_O_2_S [M − H]^−^ 413.1072, found 413.1077.

*(E,Z)-1-((4-Chlorophenyl)(p-tolyl)methylene)-2-(4-phenylthiazol-2-yl)hydrazine* (**3h**). Yield 75%, yellow solid, m.p. 172–179 °C. IR-ATR (cm^−1^) 3049, 2917, 1607, 1486, 1443, 754. ^1^H-NMR (300 MHz, DMSO-*d*_6_) δ 7.80 (d, 2H, ^3^*J =* 8.0 Hz), 7.62 (d, 1H, ^3^*J =* 8.6 Hz), 7.46 (s, 2H), 7.41–7.35 (m, 4H), 7.34–7.26 (m, 3H), 7.24–7.19 (m, 2H), 2.41 (s, 3H). ^13^C-NMR (75 MHz, DMSO-*d*_6_) δ 169.34, 149.32, 139.39, 136.78, 134.82, 132.45, 131.31, 130.40, 129.81, 129.59, 129.09, 128.87, 128.29, 127.14, 126.03, 104.49, 21.52. HRMS (ESI) calculated for C_23_H_19_ClN_3_S [M + H]^+^ 404.0988, found 404.0981.

*(E,Z)-1-((4-Methoxyphenyl)(p-tolyl)methylene)-2-(4-phenylthiazol-2-yl)hydrazine* (**3i**). Yield 77%, white solid, m.p. 279–284 °C. IR-ATR (cm^−1^) 3436, 3045, 1607, 1464, 1444, 1251, 1026, 686. ^1^H-NMR (300 MHz, CDCl_3_) δ 11.82 (s, 1H), 7.76–7.71 (m, 2H), 7.57–7.51 (m, 2H), 7.47–7.39 (m, 5H), 7.25–7.13 (m, 3H), 6.89 (d, 2H, ^3^*J =* 9.0 Hz), 3.84 (s, 3H), 2.49 (s, 3H). ^13^C-NMR (75 MHz, CDCl_3_) δ 169.43, 161.95, 159.10, 141.25, 133.53, 130.86, 130.28, 130.12, 129.52, 128.34, 127.44, 125.79, 122.74, 115.75, 113.91, 100.89, 55.45, 21.70. HRMS (ESI) calculated for C_24_H_22_N_3_OS [M + H]^+^ 400.1484, found 400.1476.

*(E,Z)-1-((4-Phenyl)-2-(4-phenylthiazol-2-yl)hydrazine)benzoic acid* (**3j**). Yield 85%, yellow solid, m.p. > 300 °C. IR-ATR (cm^−1^) 3419, 3120, 3054, 1682, 1601, 1488, 1443, 694. ^1^H-NMR (300 MHz, DMSO-*d*_6_) δ 8.12 (d, 1H, ^3^*J =* 8.5 Hz), 7.95 (d, 1H, ^3^*J =* 8.7 Hz), 7.83–7.80 (m, 2H), 7.62–7.55 (m, 3H), 7.49–7.45 (m, 2H), 7.40–7.37 (m, 3H), 7.35–7.32 (m, 2H), 7.30–7.25 (m, 3H). ^13^C-NMR (75 MHz, DMSO-*d*_6_) δ 169.52, 167.47, 148.36, 141.93, 138.19, 137.53, 134.76, 133.16, 131.82, 130.62, 129.96, 129.80, 129.32, 129.05, 128.10, 127.05, 125.96, 104.71. HRMS (ESI) calculated for C_23_H_18_N_3_O_2_S [M + H]^+^ 400.1120, found 400.1112.

### 3.6. Biological Assays

#### 3.6.1. Animals

Adult male Swiss mice weighing 20–30 g were obtained from the Central Animal Facility of the Federal University of Alfenas and were housed under controlled light (12-12 h light-dark cycle; lights on at 06:00 a.m.) with access to water and food ad libitum. The animals were allowed to habituate to the housing facilities for at least one week before the experiments began. All experiments were conducted in accordance with the Declaration of Helsinki on the welfare of experimental animals and with the approval of the Ethics Committee of the Federal University of Alfenas (protocol number 034/2016).

#### 3.6.2. In Vivo Anti-Inflammatory Assay-Croton Oil Ear Edema

The method described using croton oil for ear edema assay in mice was used in this work with groups of eight animals, according the literature [34]. The compounds (1.26 µM/ear) and vehicle (control animals) were applied topically for 30 min after the application of 20 µL croton oil (5% *v*/*v* in acetone) in the inner surface of each left ear. Indomethacin (1.26 µM/ear, inhibit edema), ketoprofen (1.26 µM/ear, inhibit edema), and dexamethasone (thin layer/ear, inhibit both edema and neutrophil recruitment) were used as positive controls. The vehicle of indomethacin was 20% distilled water in glycerin; ketoprofen was 20% acetone in glycerin. The different vehicles were evaluated and no significant difference was found in terms of their effects. The edema was measured 6 h after starting the experiment as the weight difference between 6 mm plugs taken from the left and right ears. The assay always initiated at the same time of day. The data were expressed as mean ± SEM and analyzed by one-way ANOVA following the use of Dunnett’s multiple comparison tests. The inhibition percentages were calculated according to the following formula: [(mean of values of negative control group − mean of values of treated group)/mean of values of negative control group] × 100.

#### 3.6.3. Neutrophil Recruitment 

The left-ear fragments obtained in the assay described in the previous section (Section 3.6.2) were kept in 200 µL of NaEDTA/NaCl. The enzymatic reaction was performed with 50 µL substrate reagent (tetramethylbenzidine and hydrogen peroxide) according the kit instructions for BD-OptEIA^TM^ (BD Biosciences, San Diego, CA, USA). After 10 min, the reaction ended with 2.5 M H_2_SO_4_. The absorbance was measured at 450 nm, and the results were expressed as MPO activity (determined by standard curve of neutrophil number X MPO activity) [34]. The mean ± SEM was analyzed by one-way ANOVA following Dunnett’s multiple comparison tests. The inhibition percentages are calculated according to the same formula given in the previous sections.

### 3.7. Molecular Docking Studies

The molecular docking studies were performed using Autodock Vina 1.1.2. The crystal structure coordinates of COX-1 (PDB ID: 2OYU) and COX-2 (PDB ID: 3NT1) were obtained from the Protein Data Bank (PDB) web site. The structures of all evaluated ligands and standard drugs were optimized with a fast, Dreiding-like force field by BIOVIA Discovery Studio v16.1.0.15350 [46]. COX isoenzymes were prepared by deleting crystalized water molecules and ligands and removing the repeating chain when presented. AutoDockTools 1.5.2 (Scripps Research Institute, San Diego, CA, USA) [47] was used to prepare AutoDock PDBQT format files of macromolecules and ligands. For COX isoenzymes, the dimensions of the docking grid were 18 Å × 18 Å × 18 Å and the center point coordinates were 20.524 × 50.052 × 11.247 for COX-1 and −40.957 × −51.293 × −22.318 for COX-2. The docking studies were performed using an exhaustiveness of 8 (default value) and generating a maximum of 10 binding modes. Figures were generated using BIOVIA Discovery Studio v16.1.0.15350 (Dassault Systèmes Biovia, San Diego, CA, USA) [46].

### 3.8. Structure-Activity Relationship

#### 3.8.1. Datasets

The investigation of qualitative structure-activity correlated to the type of substituents, position, and classification (anti-inflammatory activity) were carried out using the softwares SIMCA-P v 13.0.3 (Umetrics, Umea, Sweden) and Weka 3.8.0 (The University of Waikato, Hamilton, New Zealand). The dataset was analyzed in a binary matrix mode containing sample ID (names), sample groups (statistical groups), percentage of edema reduction (as *Y* variables) followed by the position and type of substituents on the carbonyl and aromatic rings (*X* variable). The existence of a group in a determined position was considered as 1 (one) and the absence, as 0 (zero). The other dataset was built including the descriptors calculated on PaDEL and the anti-inflammatory activity. The *Y* variables were the anti-edematogeninc activity: G1 (****)-standard-like activity, G2 (***)-high activity, G3 (**)-intermediate activity, G4 (*)-low activity, and G5 (ns)-non-significant; groups previously classified through ANOVA followed by Dunnett’s test. The *X* variables were the substituents (a) at the carbonyl position (carbonyl, thiosemicarbazone, 4-phenyl-2-hydrazinothiazole) and (b) at their respective positions of the benzophenone aromatic ring (C2, C4 and/or C4′ as –OH, –CH_3_, –OCH_3_, –COOH, –NO_2_, and/or Cl) were considered as the *X* variables.

#### 3.8.2. Descriptors Calculation on PaDEL

The open source software PaDEL v2.2.1 (National University of Singapore, Singapore) was used in order to evaluate the molecular descriptors to the method [48]. The adopted PaDEL molecular descriptors were: apol (sum of the atomic polarizabilities including implicit hydrogens); CrippenLogP; ETA_BetaP_ns (a measure of electron-richness of the molecule relative to molecular size); ETA_BetaP_s (a measure of electronegative atom count of the molecule relative to molecular size); ETA_dAlpha_B (A measure of count of hydrogen bond acceptor atoms and/or polar surface area); FMF (Complexity of a molecule); Lipoaffinity (lipoaffinity index); maxHBa (maximum E-States for (strong) hydrogen bond acceptors); maxHBd (maximum E-States for (strong) hydrogen bond donors); Mi (mean of the first ionization potential); MLogP (MannholdLogP); MV (mean atomic van der Waals volumes scaled on carbon atom); naAromAtom (number of aromatic atoms); nAromBond (number of aromatic bonds); nBondsD (number of double bonds); nFRing (number of fused rings); nHBAcc (Number of hydrogen bond acceptors (using CDK H bond acceptor count descriptor algorithm); nHBAcc_Lipinski (number of hydrogen bond acceptors using Lipinski’s definition); nHBDon number of hydrogen bond donors (using CDK H bond donor count descriptor algorithm); nHBDon_Lipinski (number of hydrogen bond donors using Lipinski’s definition); nRing (number of rings); nRotB (number of rotable bonds); Sp (sum of atomic polarizability scaled on carbon atoms); Spe (sum of atomic pauling eletronegativities); SV (sum of atomic van der Waals volumes scaled on carbon atom); TopoPSA (topological polar surface area); VABC (Van der Waals volume calculated using the method proposed in Zhao [49]); XLogP (LogP).

#### 3.8.3. Multivariate Statistical Analysis

The datasets were analyzed by the supervised (O2PLS-DA^TM^, (Umetrics, Umea, Sweden) multivariate method on the SIMCA-P+ v13.0.3 software (Umetrics, Umea, Sweden), while using no data scaling. The method was best fitted using four components (2 Predictive X-Y and 2 Orthogonal X (OPLS); *n* = 30; R^2^X (cum) of 0.554; confidence level on parameters: 0.05). The analysis were supervised by anti-inflammatory activity (*Y* variable) (statistical classification by one-way ANOVA followed by Dunnett’s test carried out on the GraphPad Prism version 6.00 for Windows (GraphPad Software, La Jolla, CA, USA). The substituents position on the benzophenone derivatives were the *X* variables. The coefficient plot overview, VIP values, score and loading plots were obtained with the purpose of associating the most important substituents to the anti-inedematogenic activity.

The attributes (substituent groups) correlated with anti-edematogenic activity were additionally evaluated by attribute evaluator CfsSubsetEval (-P1 -E1) and the search method BestFirst (-D1 -N5) on the Weka 3.8.0 software using 10 fold cross-validations.

The evaluation of correlation among descriptors calculated on PaDEL and the anti-inflammatory activity was elaborated through an O2PLS-DA model using 4 components (1 Predictive X-Y and 3 Orthogonal X (OPLS); *n* = 33; RX^2^ (cum): 0.910; confidence level on parameters: 0.05).

## 4. Conclusions

In this work, we described the molecular docking, synthesis, and anti-inflammatory activity of a new series of benzophenone derivatives, inspired by ketoprofen, attached to a thiosemicarbazide and a thiazole nucleus. The molecular docking studies showed a great potential of inhibition of the proposed substances against COX isoenzymes. The derivatives **3e**, **3f**, **3h** and **3j** showed potent edema inhibition (higher than 72%) while the derivatives **2e**, **3a** and **3c** were able to inhibit both edema and neutrophil recruitment. Thus, these compounds with dual anti-inflammatory property showed a mechanism of action which differed from those of usual anti-inflammatory drugs. Compounds with this action mechanism can have higher efficacy and fewer side effects than commercial NSAIDs and corticosteroids. It is important to highlight that the results were confirmed by molecular docking and statistical analysis provided information on the substitution pattern which was responsible for the activity. The presence of 4-phenyl-2-hydrazinothiazole and the absence of OCH_3_, exactly on the C4′ position is highly correlated to the anti-edematogenic activity. Substituents in determined positions can modulate the affinity of these compounds and the enzymes. Features as aromatic bonds, lipoaffinity, polarizabilities, and electrongativities are also strongly correlated to the anti-edematogenic properties of these compounds. Bulky substituents appear to interfere on the inhibition of neutrophil recruitment. Therefore, these results can also be useful toward the design of new potential anti-inflammatory and development of new drugs.

## Figures and Tables

**Table 1:** Docking results of planned compounds and standard drugs in COX-1 and COX-2 active sites.

Compounds	Predicted Binding Energy (kcal/mol)	
	COX-1	COX-2
	PDB ID: 2OYU	PDB ID: 3NT1
	(Regioisomer)	(Regioisomer)
**3a**	−8.1	−8.0
**3b**	−8.2 (*E*)/−8.4 (*Z*)	−8.5 (*E*)/−8.0 (*Z*)
**3c**	−8.1 (*E*)/−7.9 (*Z*)	−7.9 (*E*)/−7.6 (*Z*)
**3d**	−8.2	−7.9
**3e**	−8.0 (*E*)/−7.3 (*Z*)	−7.4 (*E*)/−7.6 (*Z*)
**3f**	−7.9 (*E*)/−7.4 (*Z*)	−7.2 (*E*)/−7.7 (*Z*)
**3g**	−8.2 (*E*)/−9.2 (*Z*)	−8.7 (*E*)/−8.7 (*Z*)
**3h**	−8.6 (*E*)/−8.7 (*Z*)	−8.2 (*E*)/−8.5 (*Z*)
**3i**	−8.2 (*E*)/−8.7 (*Z*)	−8.2 (*E*)/−8.7 (*Z*)
**3j**	−7.8 (*E*)/−8.7 (*Z*)	−9.1 (*E*)/−8.2 (*Z*)
**Indomethacin**	−7.8	−5.7
***S*-Ketoprofen**	−8.8	−8.9

**Table 2:** Docking results of thiosemicarbazones **2a**–**2j** in COX-1 and COX-2 active sites.

Compound	Predicted Binding Energy (kcal/mol)	
	COX-1	COX-2
	PDB ID: 2OYU	PDB ID: 3NT1
	(Regioisomer)	(Regioisomer)
**2a**	−8.1	−7.4
**2b**	−8.6 (*E*)/−8.6 (*Z*)	−8.0 (*E*)/−8.2 (*Z*)
**2c**	−8.1 (*E*)/−8.1 (*Z*)	−7.6 (*E*)/−7.8 (*Z*)
**2d**	−8.1	−6.8
**2e**	−7.7 (*E*)/−7.7 (*Z*)	−7.4 (*E*)/−7.1 (*Z*)
**2f**	−7.9 (*E*)/−8.1 (*Z*)	−7.7 (*E*)/−7.5 (*Z*)
**2g**	−9.0 (*E*)/−9.3 (*Z*)	−8.0 (*E*)/−8.0 (*Z*)
**2h**	−9.0 (*E*)/−9.0 (*Z*)	−7.3 (*E*)/−7.8 (*Z*)
**2i**	−8.8 (*E*)/−9.0 (*Z*)	−7.4 (*E*)/−7.8 (*Z*)
**2j**	−8.6 (*E*)/−8.7 (*Z*)	−8.5 (*E*)/−8.6 (*Z*)

**Table 3:** Inhibition of ear edema and on neutrophil recruitment (%) by the control groups and synthesized compounds.

Compounds	Ear Edema	Neutrophil Recruitment	Compounds	Ear Edema	Neutrophil Recruitment
**2a**	28 **	ns	**3c**	55 ****	66 **
**2b**	42 ****	ns	**3d**	64 ****	ns
**2c**	ns	ns	**3e**	73 ****	ns
**2d**	50 ****	ns	**3f**	74 ****	ns
**2e**	69 ****	52 *	**3g**	51 ****	ns
**2f**	72 ****	ns	**3h**	72 ****	ns
**2g**	74 ****	ns	**3i**	47 ****	ns
**2h**	76 ****	ns	**3j**	75 ****	ns
**2i**	ns	ns	**Indomethacin**	71 ****	ns
**2j**	38 ****	ns	**Ketoprofen**	68 ****	ns
**3a**	48 ****	68 **	**Dexamethasone**	82 ****	54 *
**3b**	69 ****	ns	**Vehicle**	0	0

Statistical differences between the treated groups and the control group (vehicle) were evaluated by ANOVA followed by Dunnett’s test. The asterisks denote the levels of significance in comparison with vehicle (*n* = 8). G1 (**** *p* ≤ 0.0001); G3 (** *p* ≤ 0.01); G4 (* *p* ≤ 0.05), ns = not significant.

**Table 4:** VIP values higher than 1 of the O2PLS model for the PaDEL descriptors. The list is based only on VIPs > 1.

Var ID	VIP	Coeff. G1 (****)	Coeff. G5 (ns)
Sv	1.50867	0.041	−0.022
apol	1.50866	0.041	−0.022
VABC	1.50828	0.041	−0.022
Sp	1.50668	0.041	−0.022
CrippenLogP	1.45515	0.039	−0.021
Spe	1.44239	0.039	−0.021
nRing	1.42547	0.038	−0.021
MLogP	1.30142	0.036	−0.019
LipoaffinityIndex	1.26585	0.034	−0.018
XLogP	1.24645	0.034	−0.018
nRotB	1.07619	0.030	−0.016
nAromBond	1.0697	0.029	−0.016
naAromAtom	1.03784	0.029	−0.015

The asterisks denote the levels of significance in comparison with vehicle (*n* = 8). G1 (**** *p* ≤ 0.0001); G5 (ns = not significant).

## Supplementary Materials

Supplementary materials are available online.
