# Supplementary material for: Design and Synthesis of New Benzophenone Derivatives with In Vivo Anti-Inflammatory Activity through Dual Inhibition of Edema and Neutrophil Recruitment
Source: Molecules. 2018 Jul 26;23(8):1859. doi: 10.3390/molecules23081859 (PMC6222881; doi:10.3390/molecules23081859)
Supplement: Supplementary file 1 [file molecules-23-01859-s001.pdf]

Design and Synthesis of New Benzophenone Derivatives with in Vivo Anti-Inflammatory Activity Through Dual Inhibition of Edema and Neutrophil Recruitment

Jaqueline P. Januario, Thiago B. de Souza, Stefânia N. Lavorato, Tatiane C. S. Maiolini, Olívia S. Domingos, João L. Baldim, Laís R. S. Folquitto, Marisi G. Soares, Daniela A. Chagas-Paula, Danielle F. Dias, Marcelo H. dos Santos.

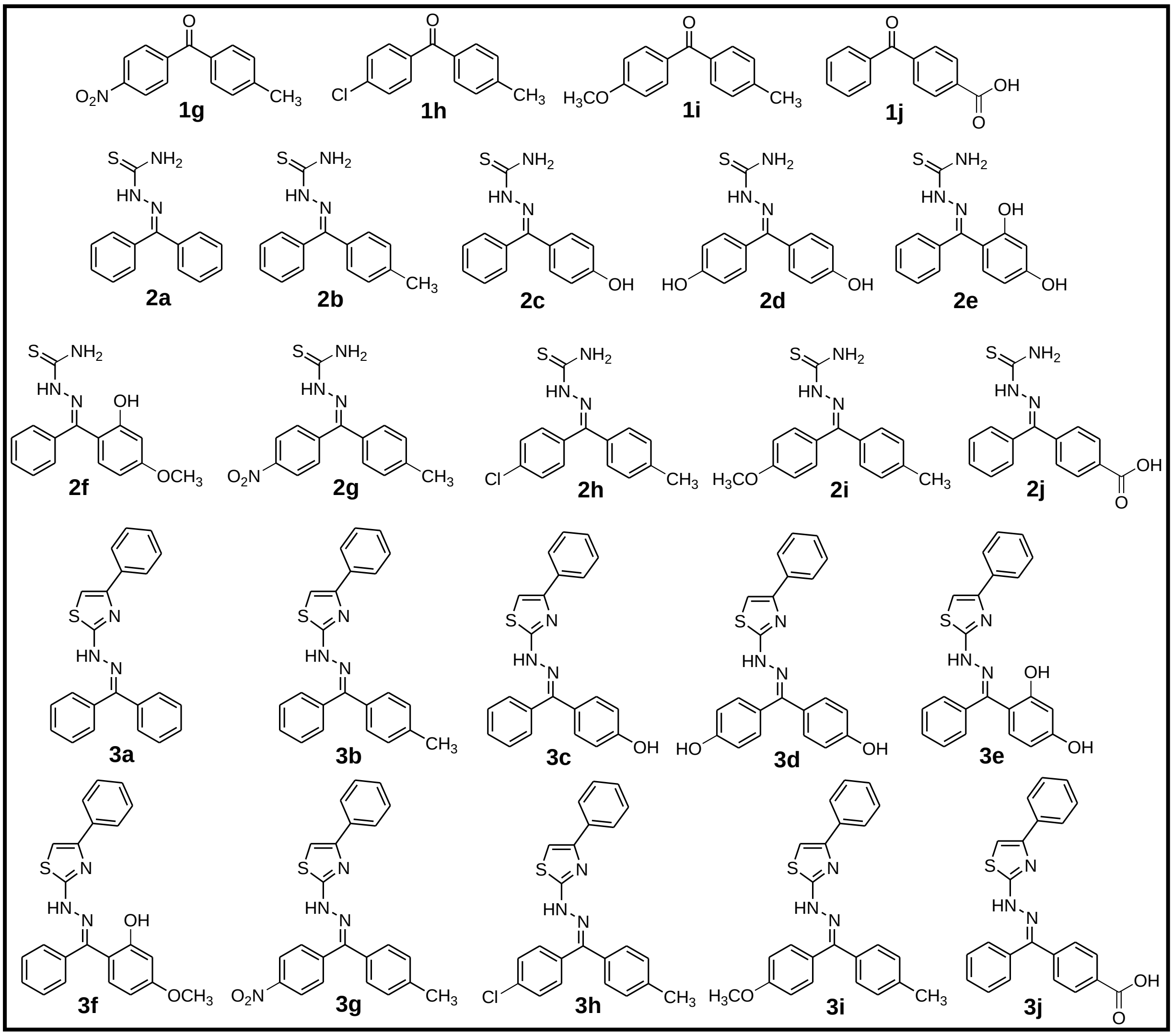

SUPPLEMENTARY MATERIALS

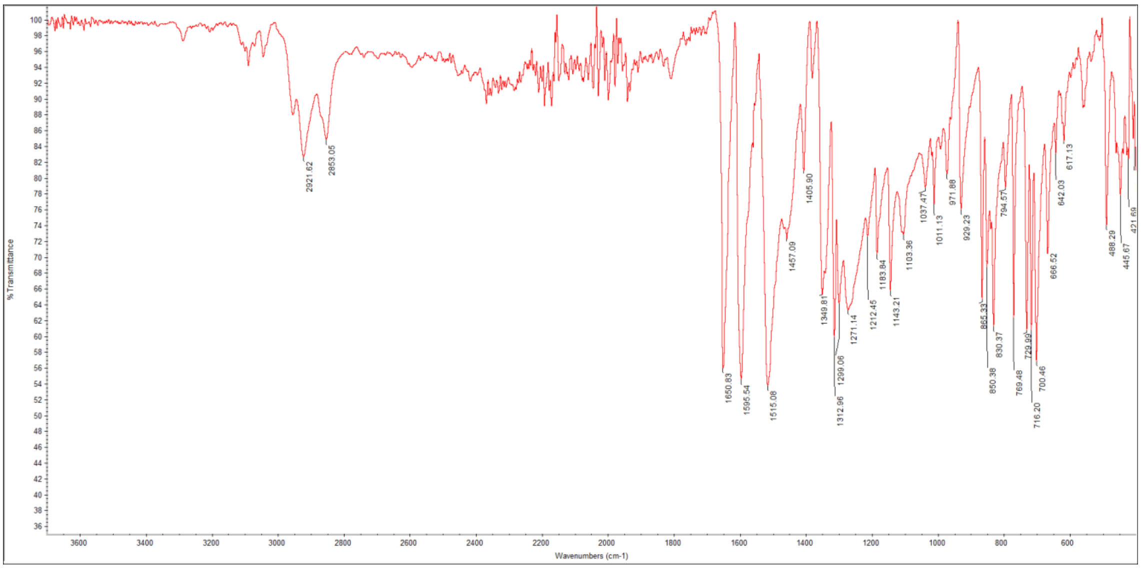

Figure S1: IR spectra of **1g**.

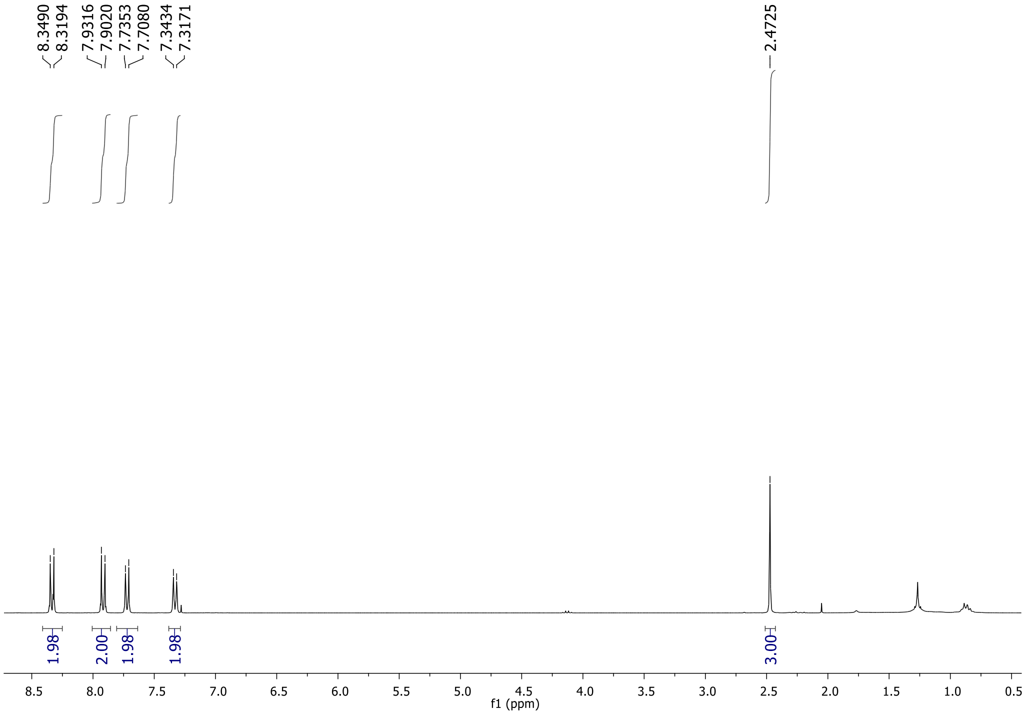

Figure S2: ^{1}H NMR spectra of **1g** ($CDCl_3$, 300 MHz).

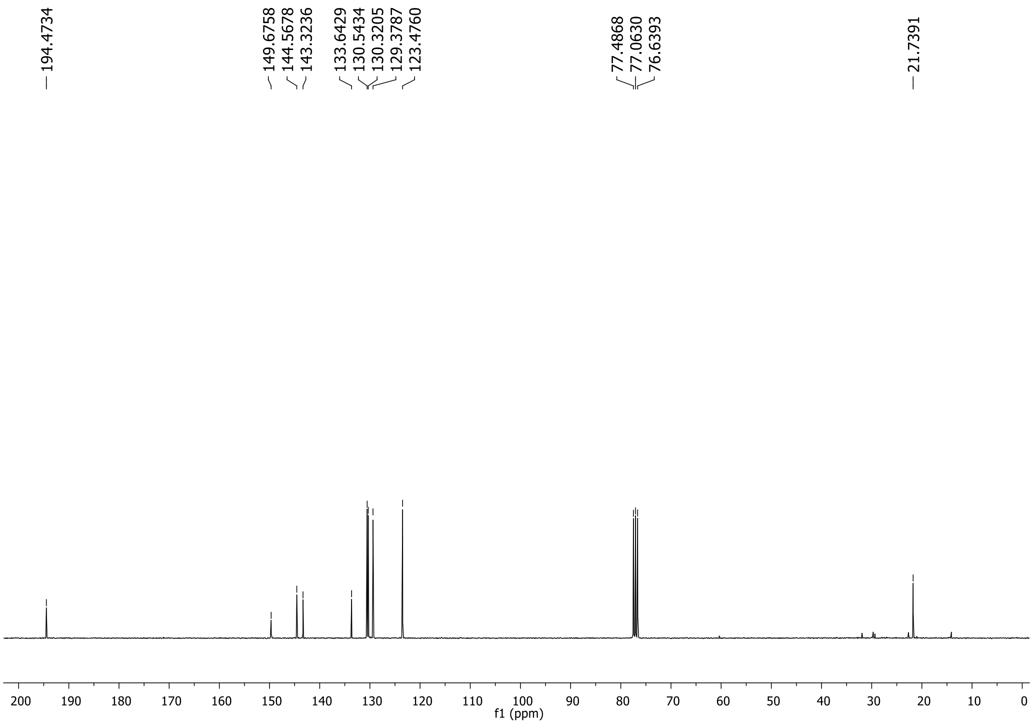

Figure S3: ^{13}C NMR spectra of **1g** ($CDCl_3$, 75 MHz).

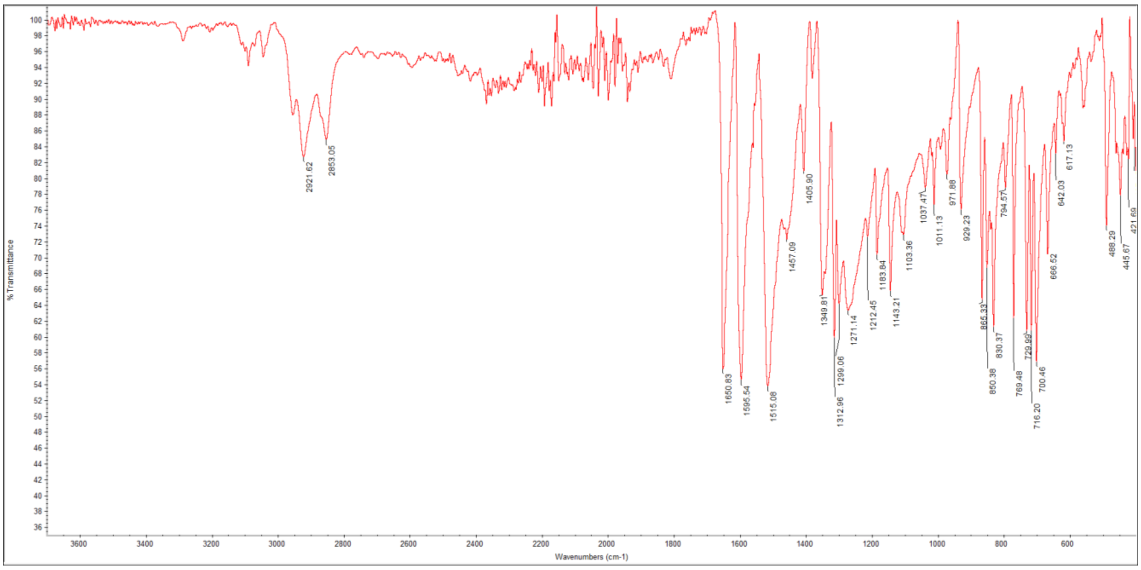

Figure S4: IR spectra of **1h**.

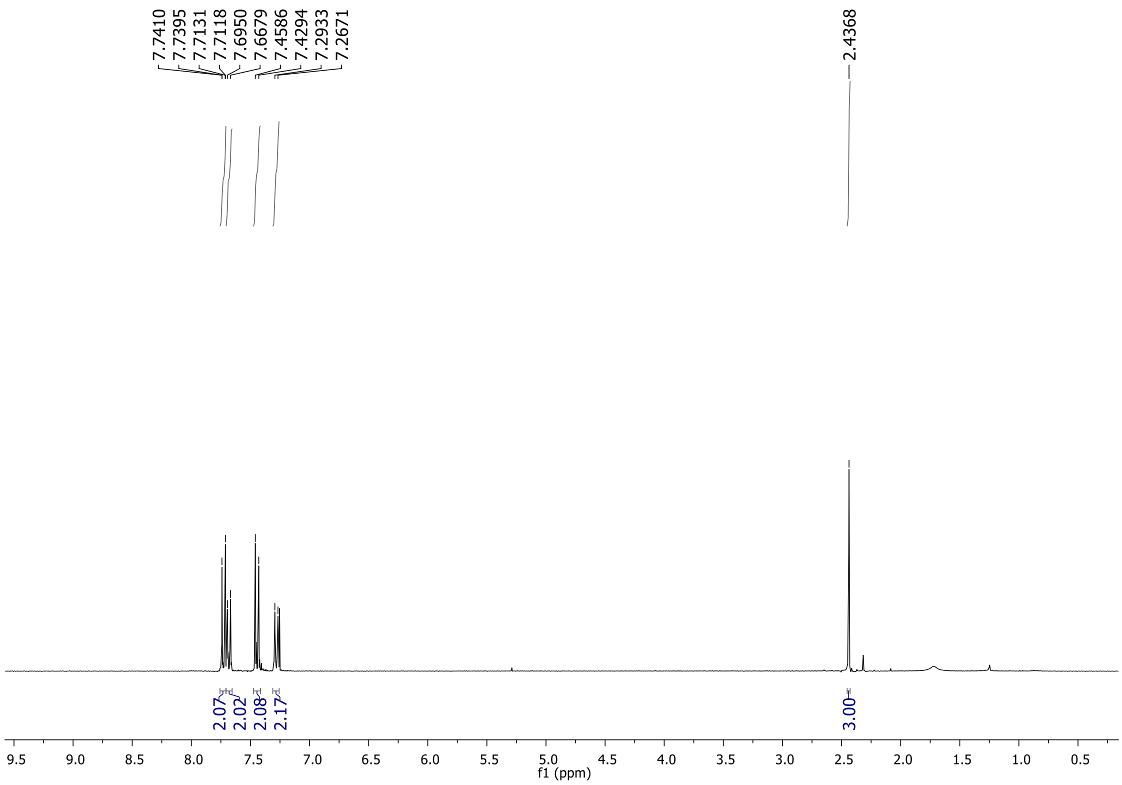

Figure S5: ^{1}H NMR spectra of **1h** ($CDCl_3$, 300 MHz).

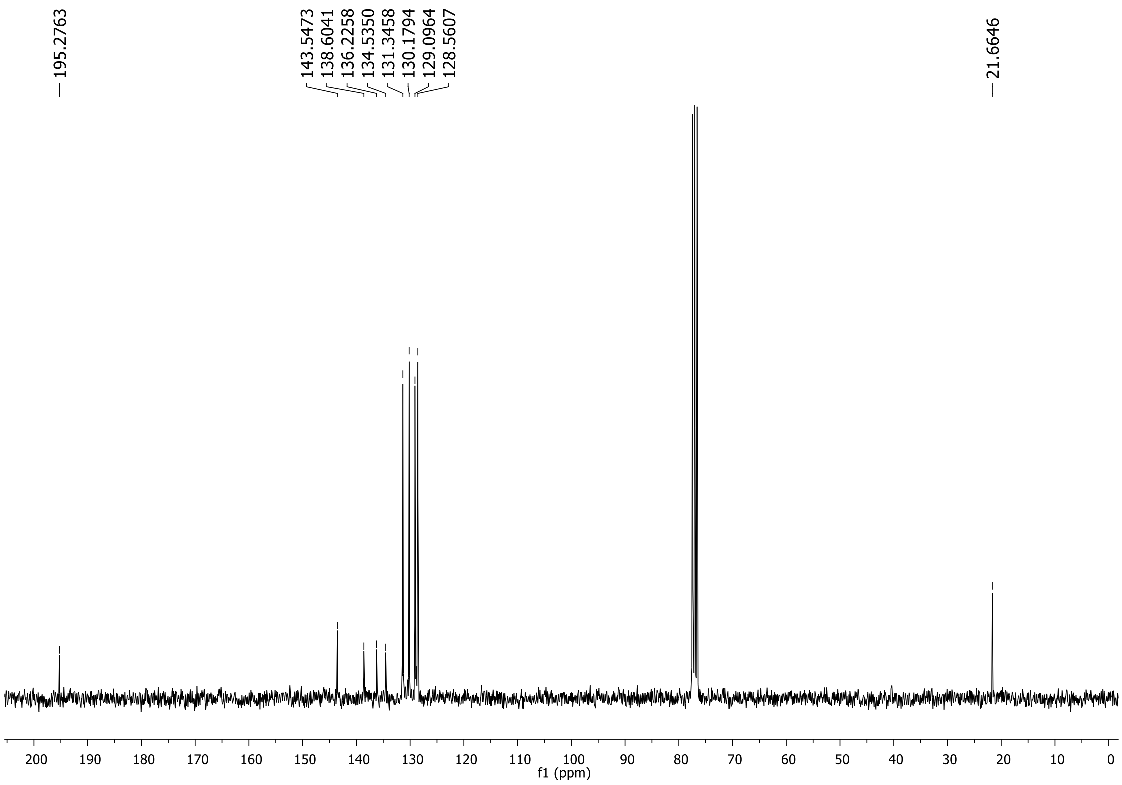

Figure S6: ^{13}C NMR spectra of **1h** ($CDCl_3$, 75 MHz).

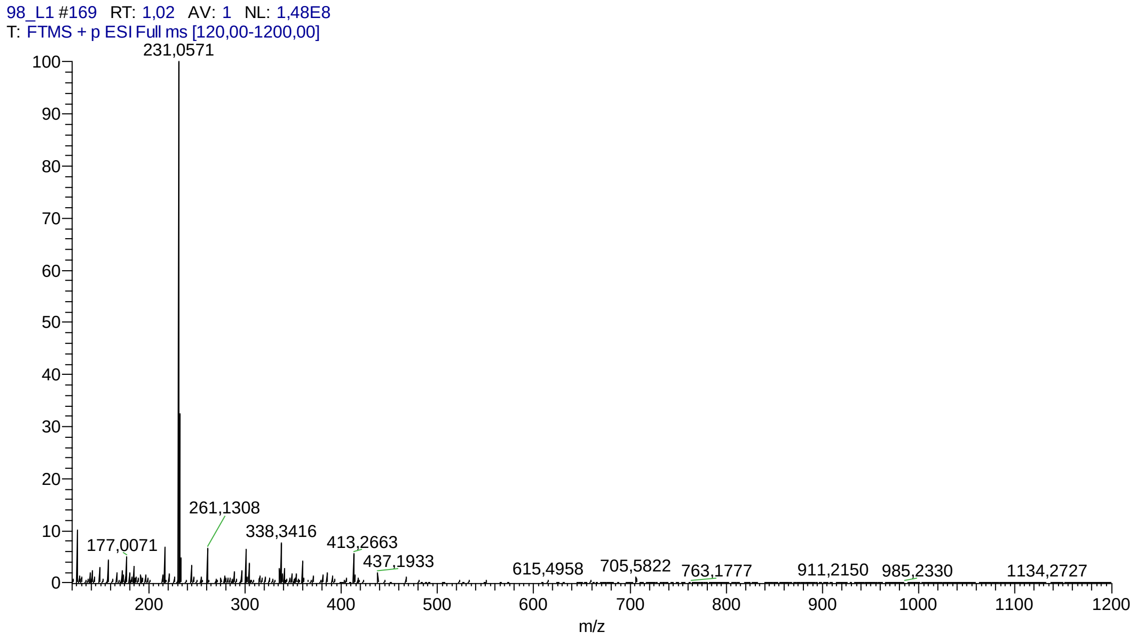

Figure S7: HRMS spectra of **1h**.

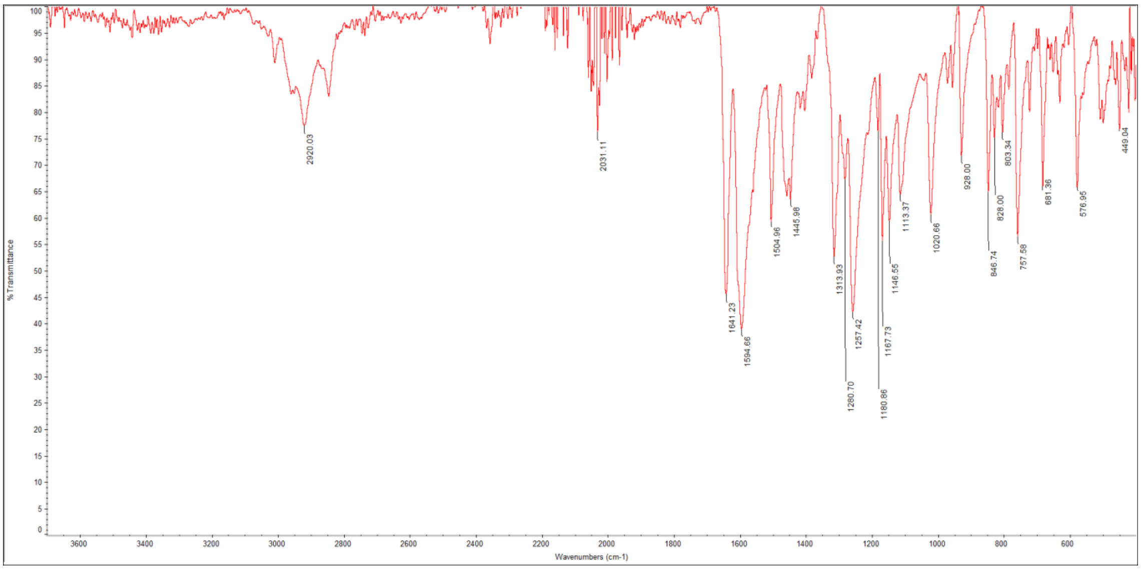

Figure S8: IR spectra of **1i**.

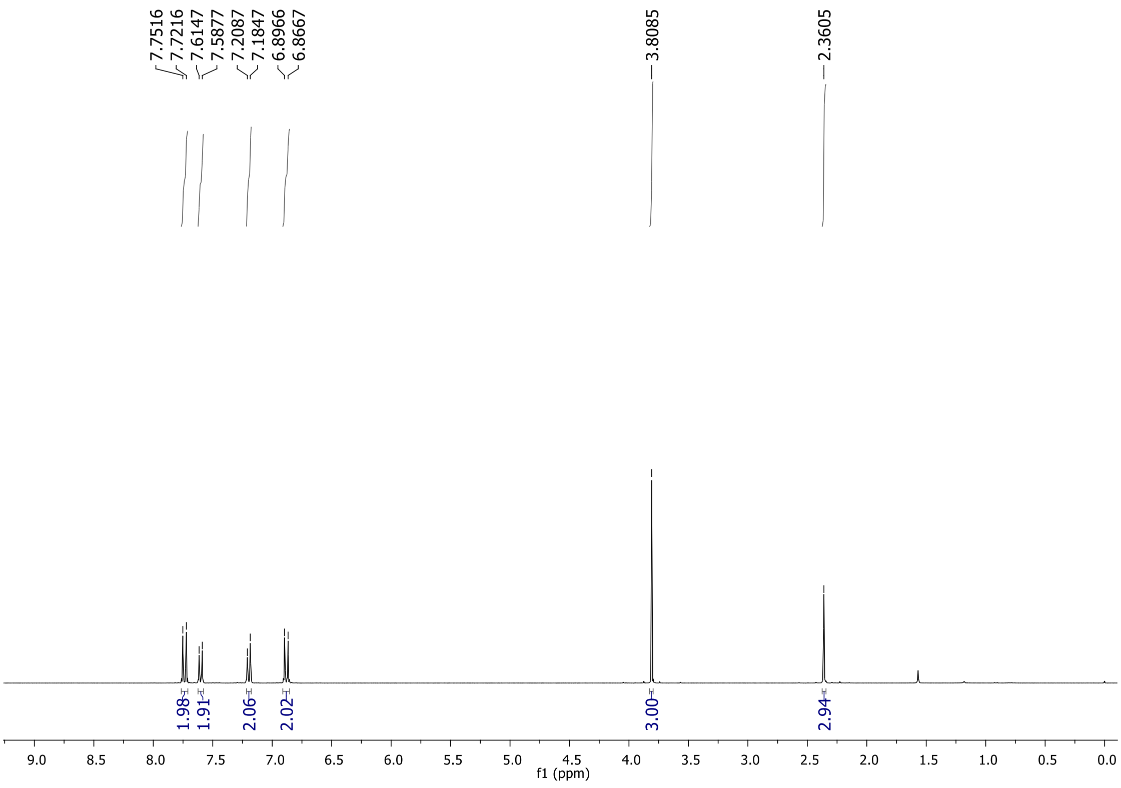

Figure S9: ^{1}H NMR spectra of **1i** ($CDCl_3$, 300 MHz).

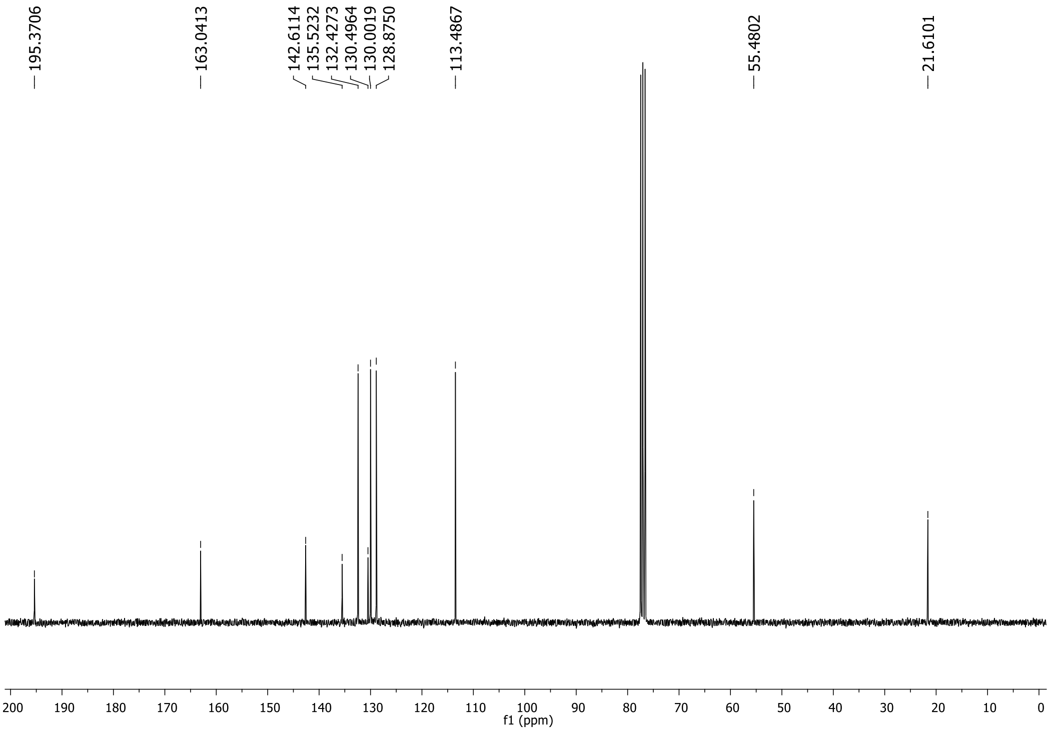

Figure S10: ^{13}C NMR spectra of **1i** ($CDCl_3$, 75 MHz).

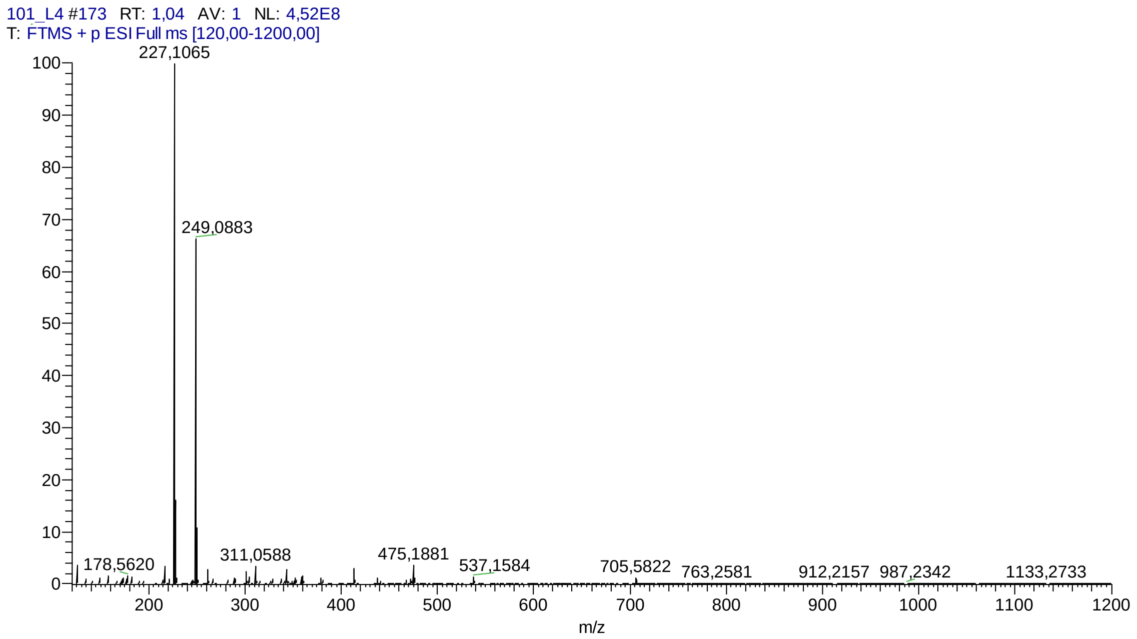

Figure S11: HRMS spectra of **1i**.

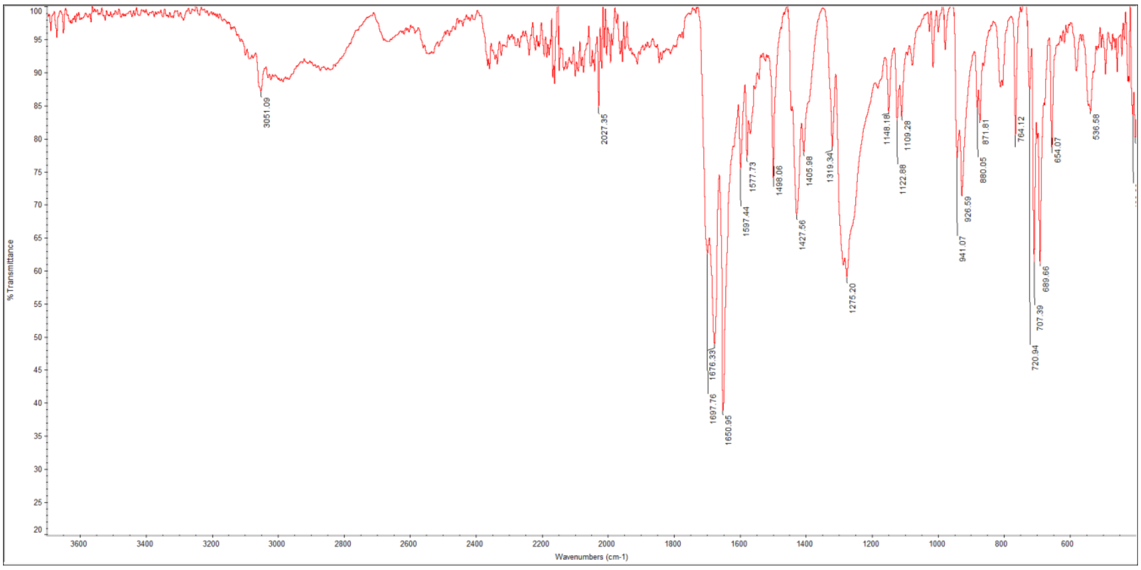

Figure S12: IR spectra of **1j**.

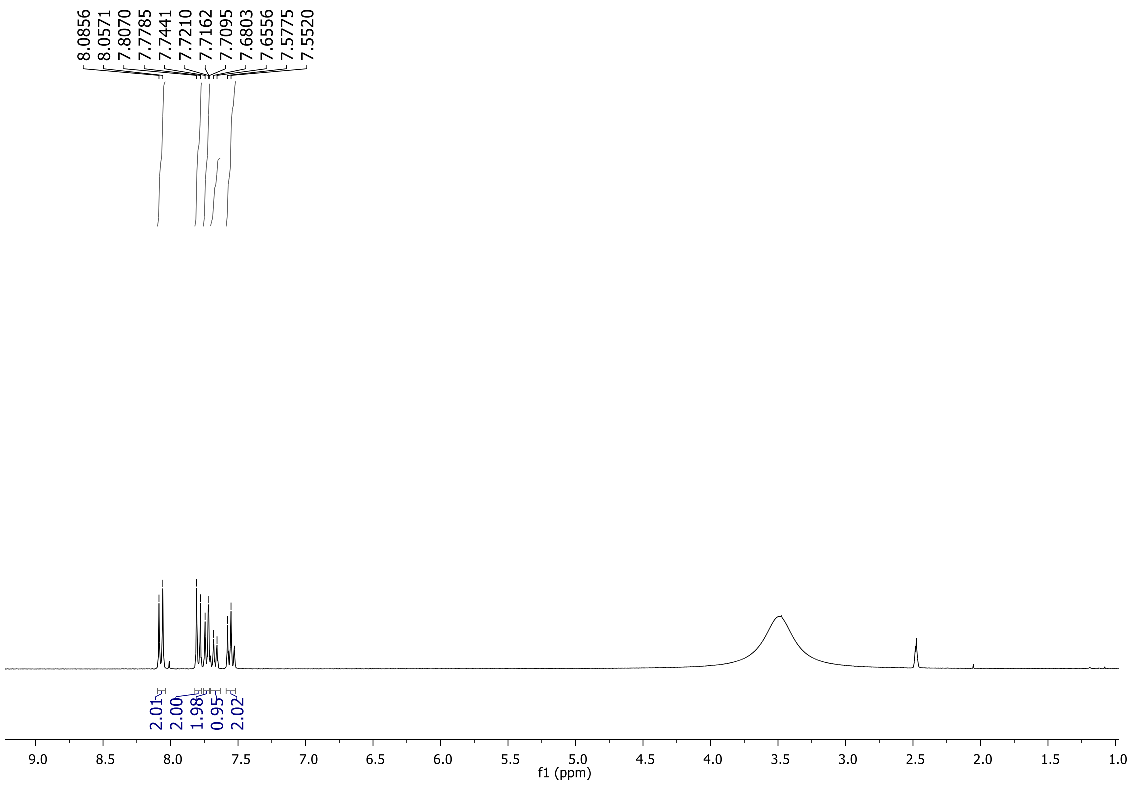

Figure S13: ^{1}H NMR spectra of **1j** (DMSO-d_6, 300 MHz).

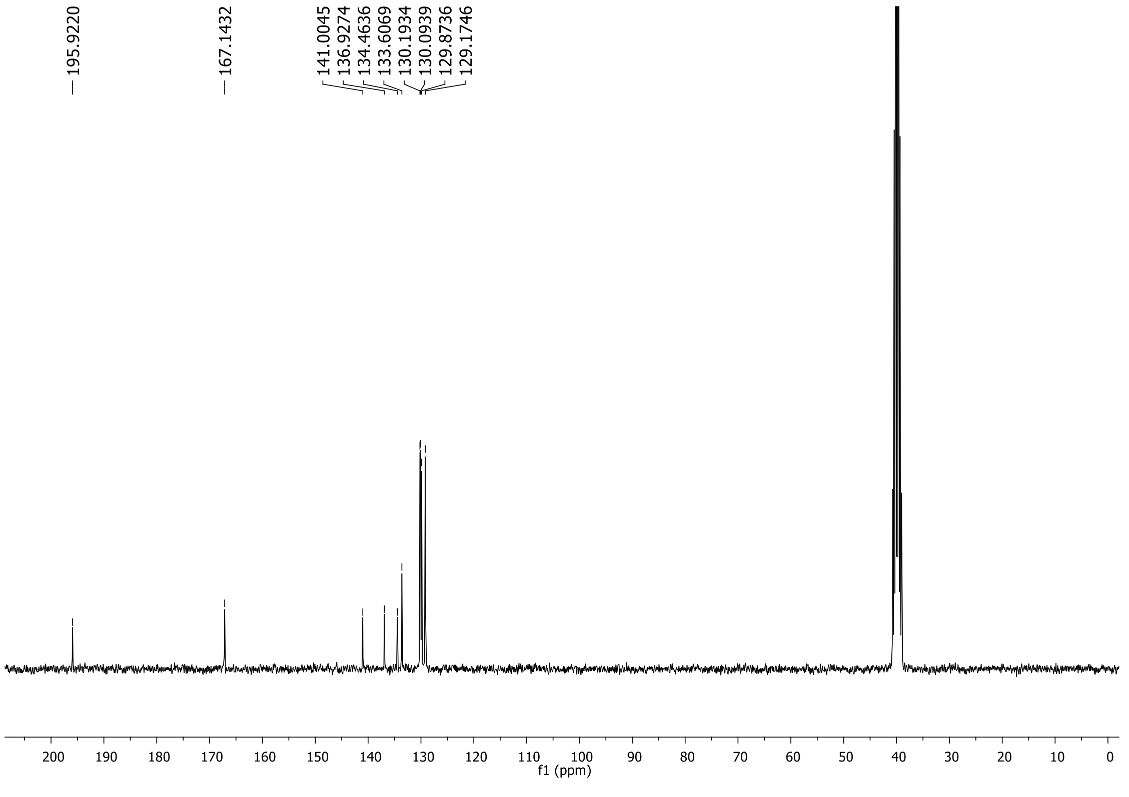

Figure S14: ^{13}C NMR spectra of **1j** (DMSO-d_6, 75 MHz).

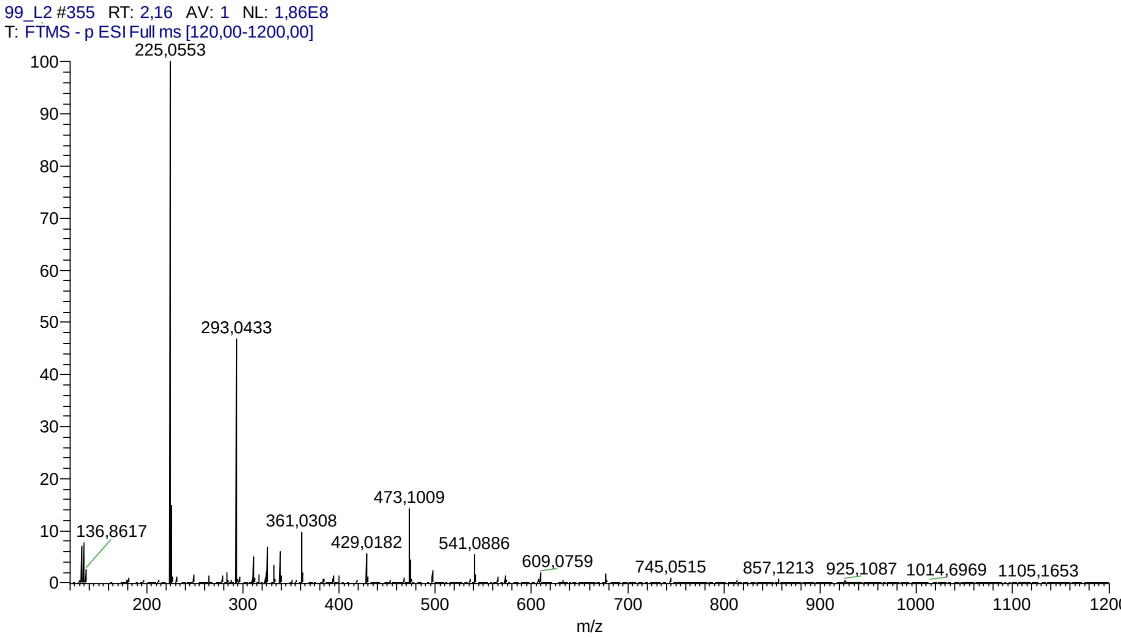

Figure S15: HRMS spectra of **1j**.

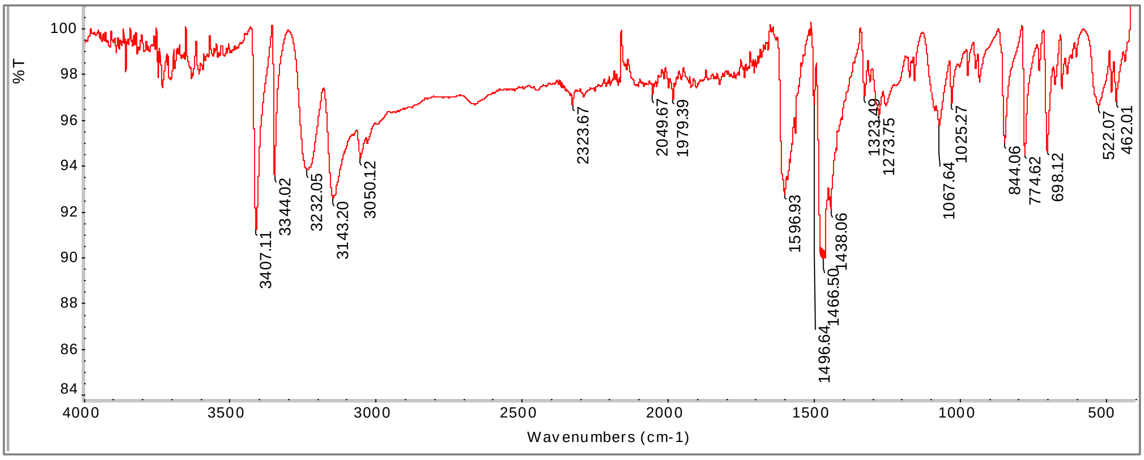

Figure S16: IR spectra of **2a**.

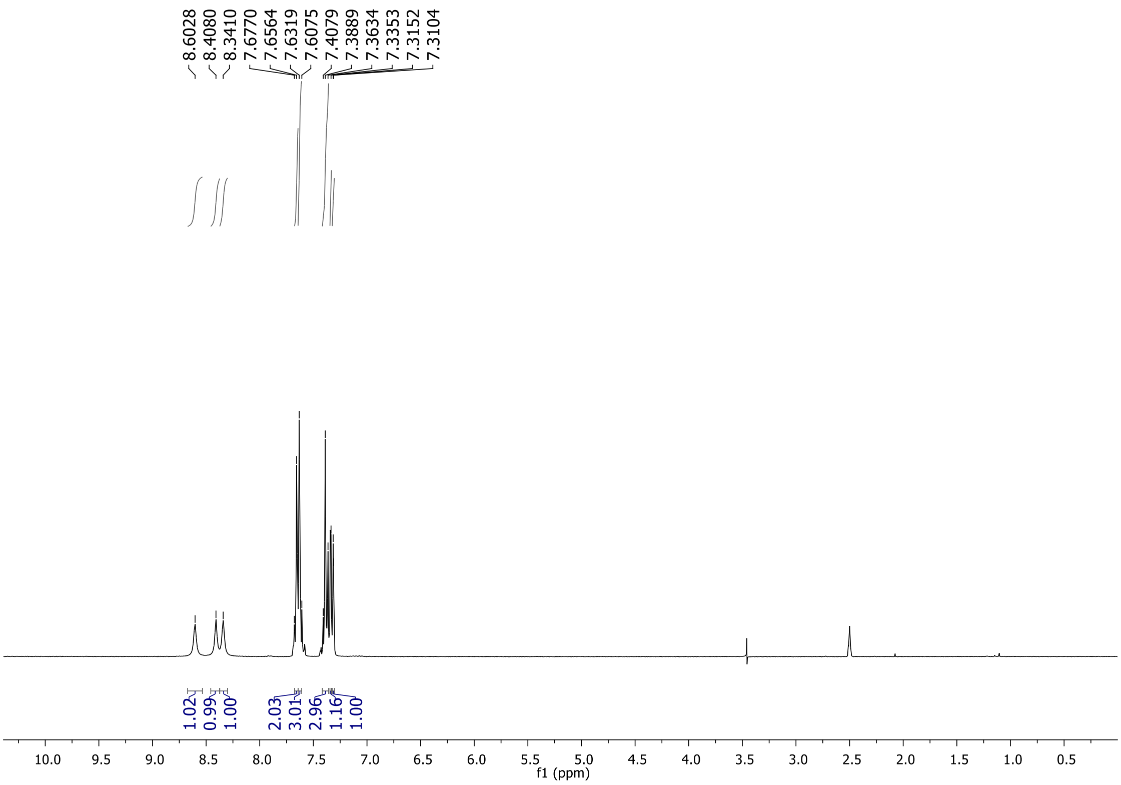

Figure S17: ^{1}H NMR spectra of **2a** (DMSO-d_6, 300 MHz).

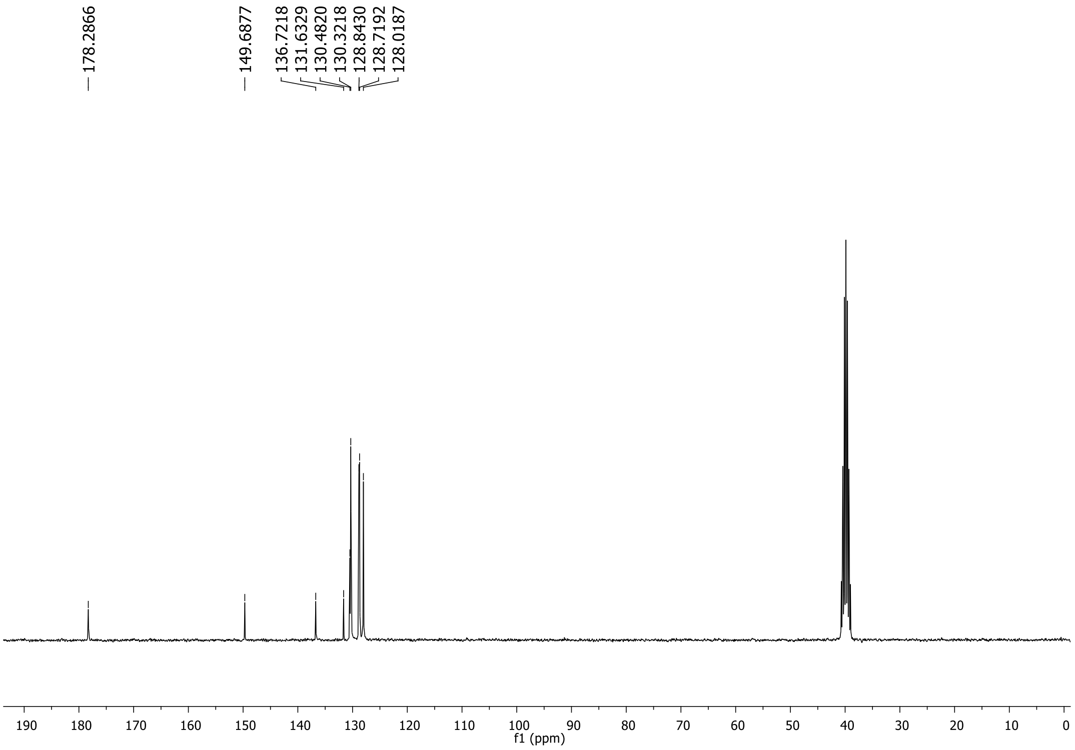

Figure S18: ^{13}C NMR spectra of **2a** (DMSO-d_6, 75 MHz).

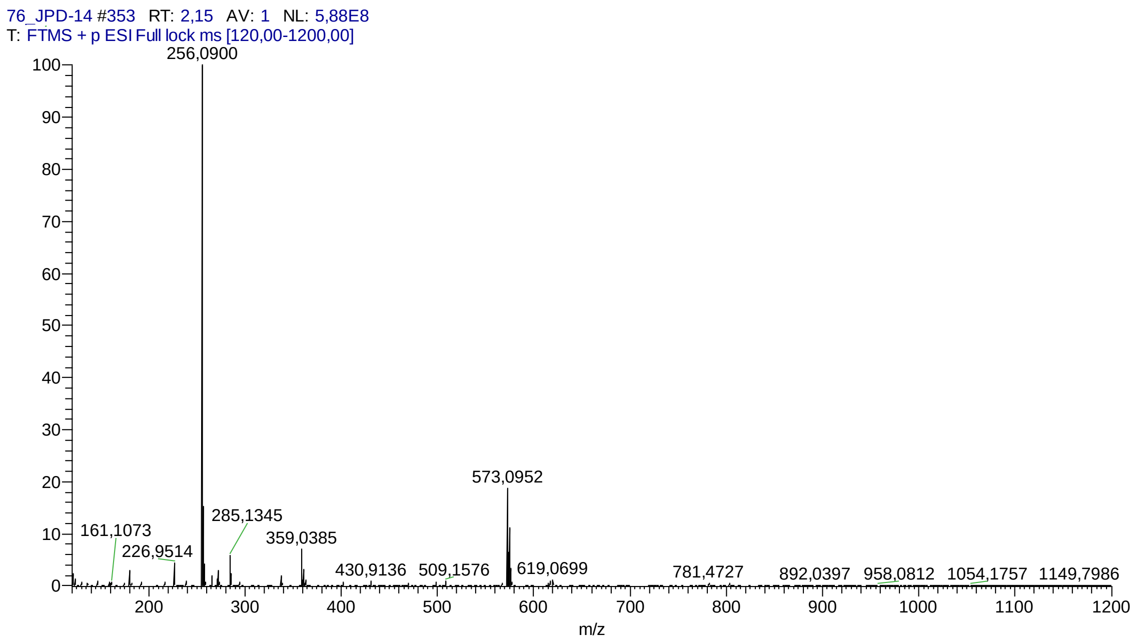

Figure S19: HRMS spectra of **2a**.

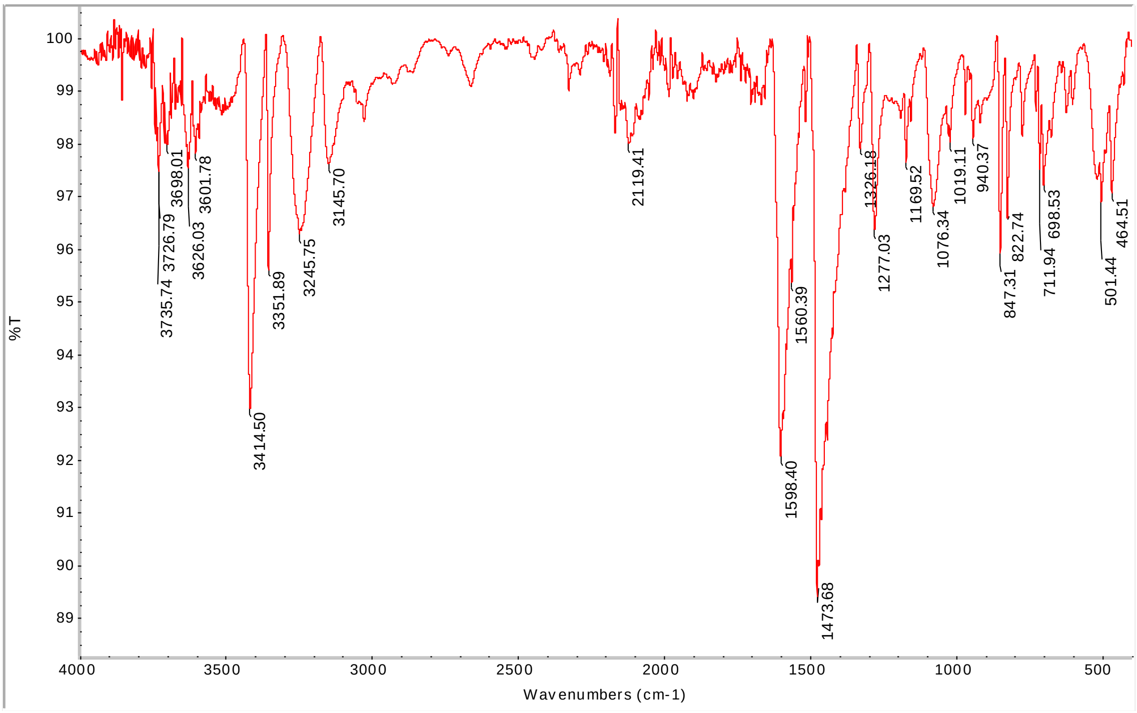

Figure S20: IR spectra of **2b**.

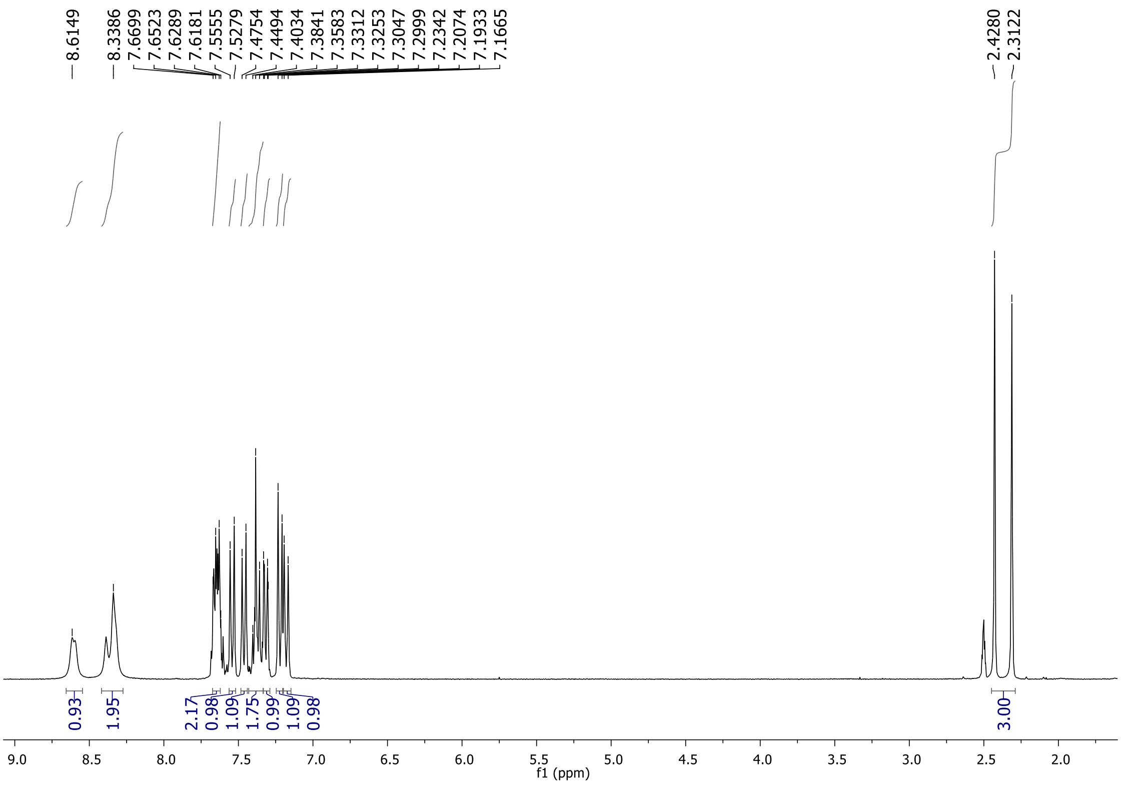

Figure S21: ^{1}H NMR spectra of **2b** (DMSO-d_6, 300 MHz).

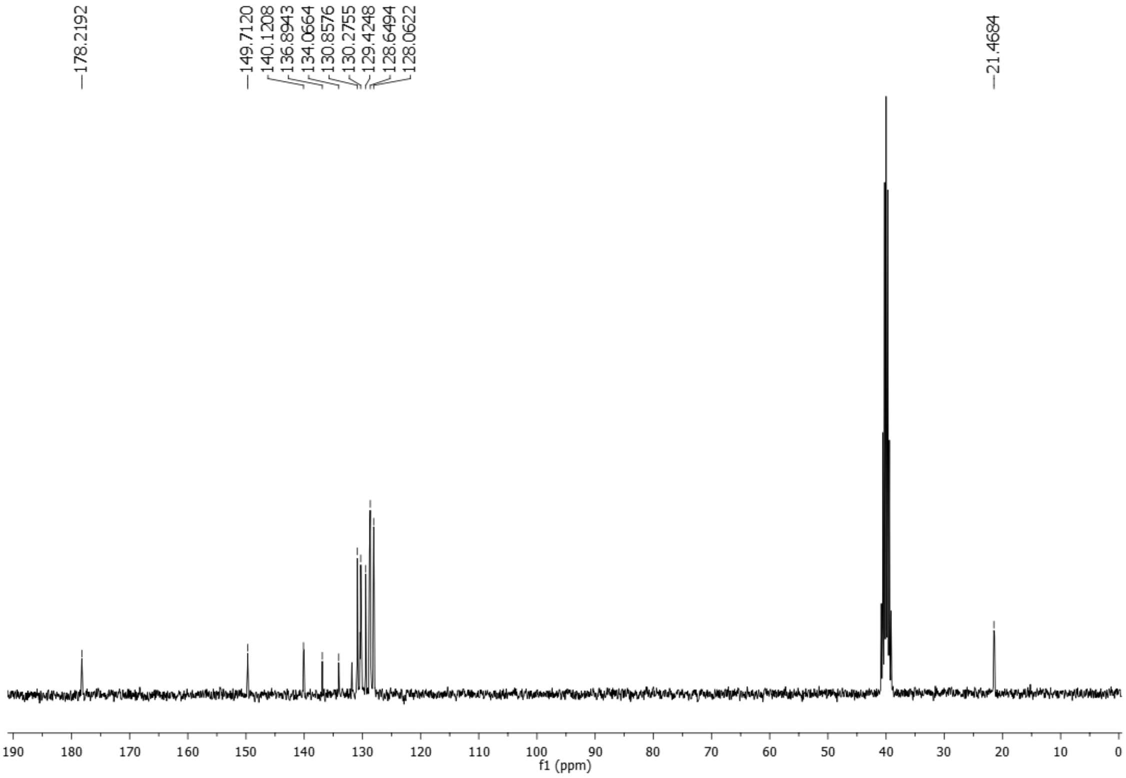

Figure S22: ^{13}C NMR spectra of **2b** (DMSO-d_6, 75 MHz).

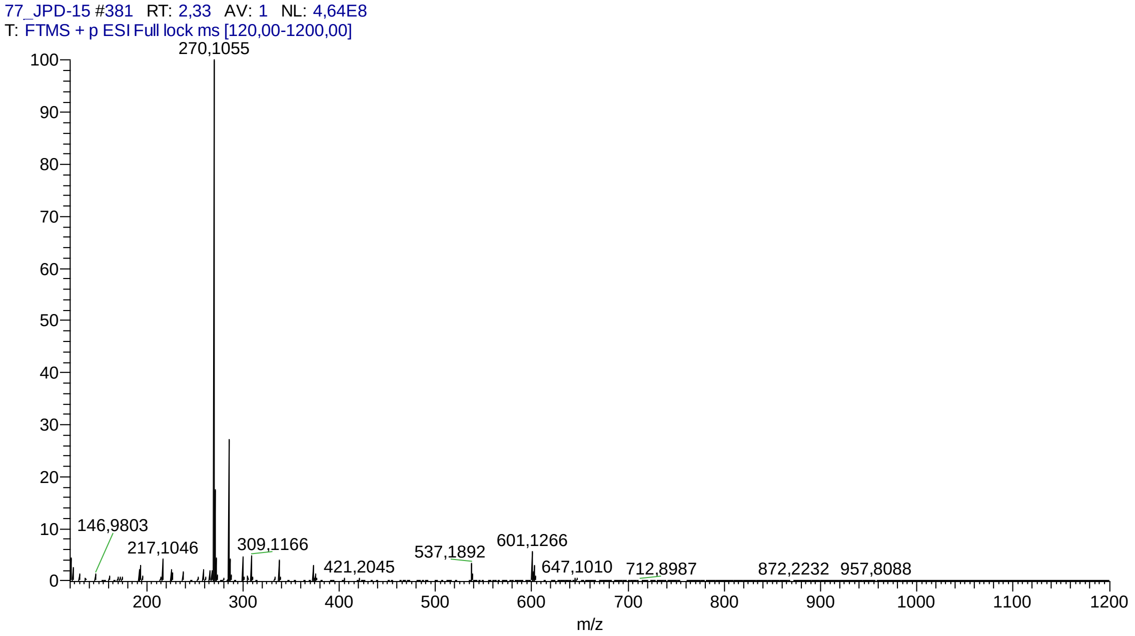

Figure S23: HRMS spectra of **2b**.

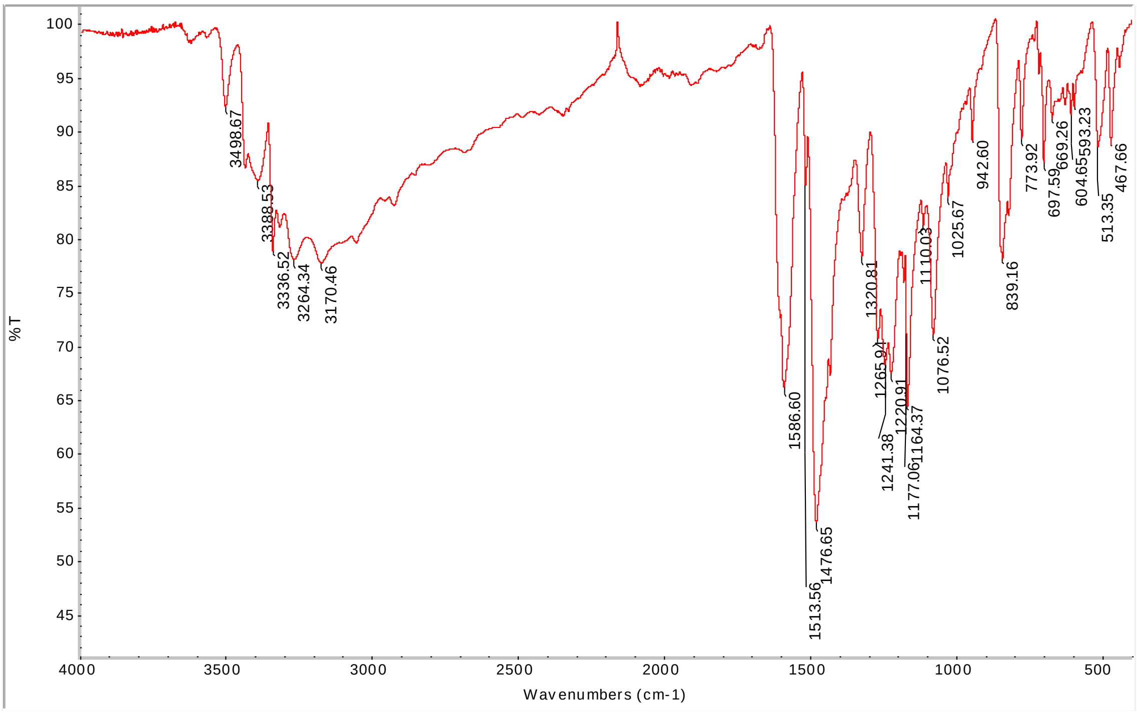

Figure S24: IR spectra of **2c**.

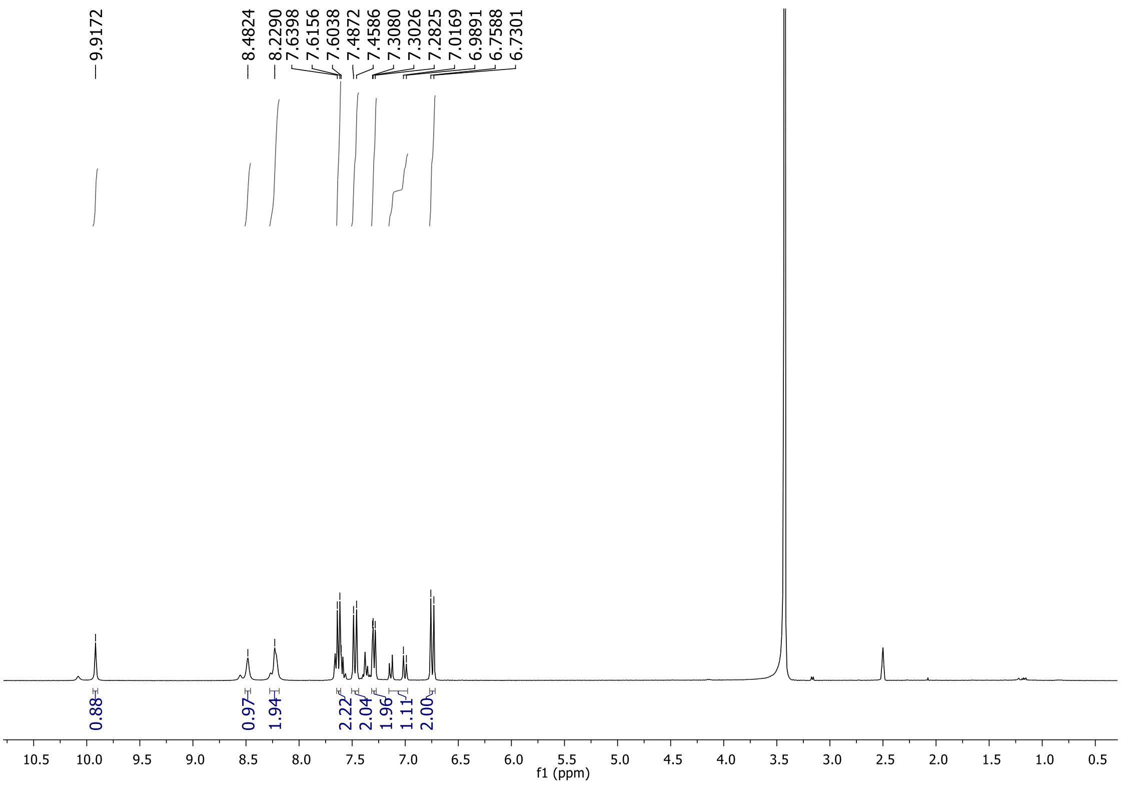

Figure S25: ^{1}H NMR spectra of **2c** (DMSO-d_6, 300 MHz).

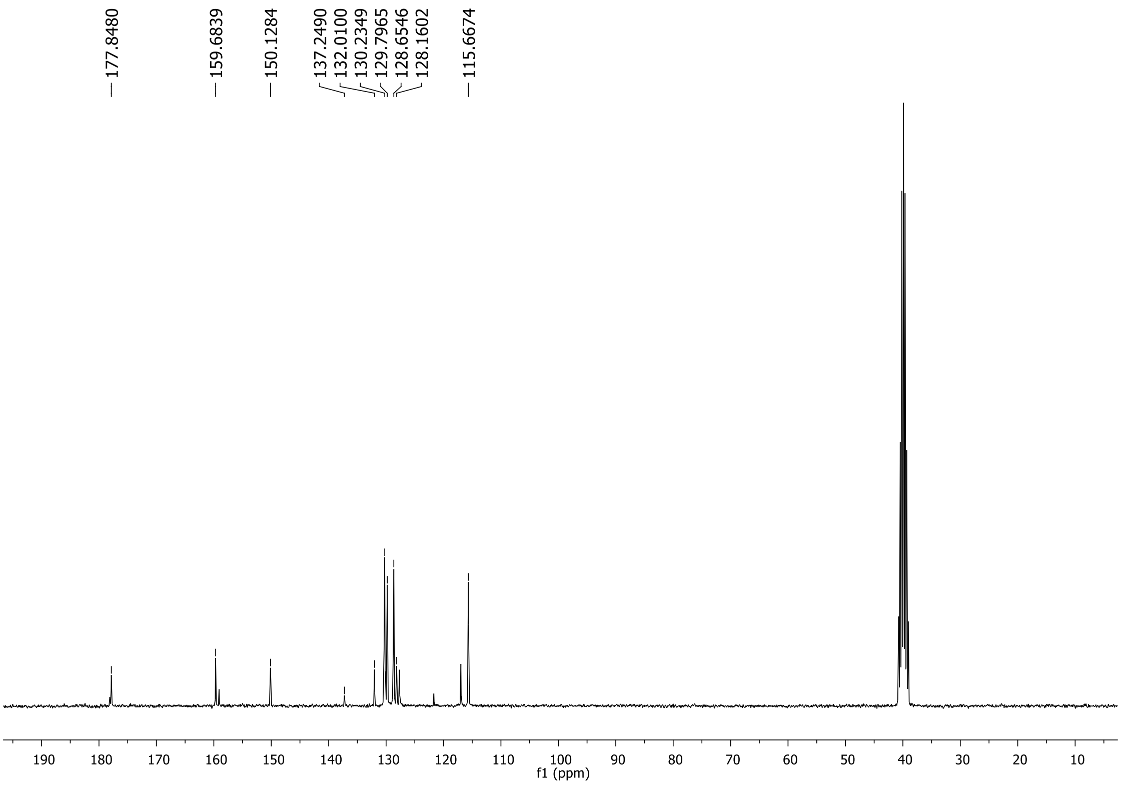

Figure S26: ^{13}C NMR spectra of **2c** (DMSO-d_6, 75 MHz).

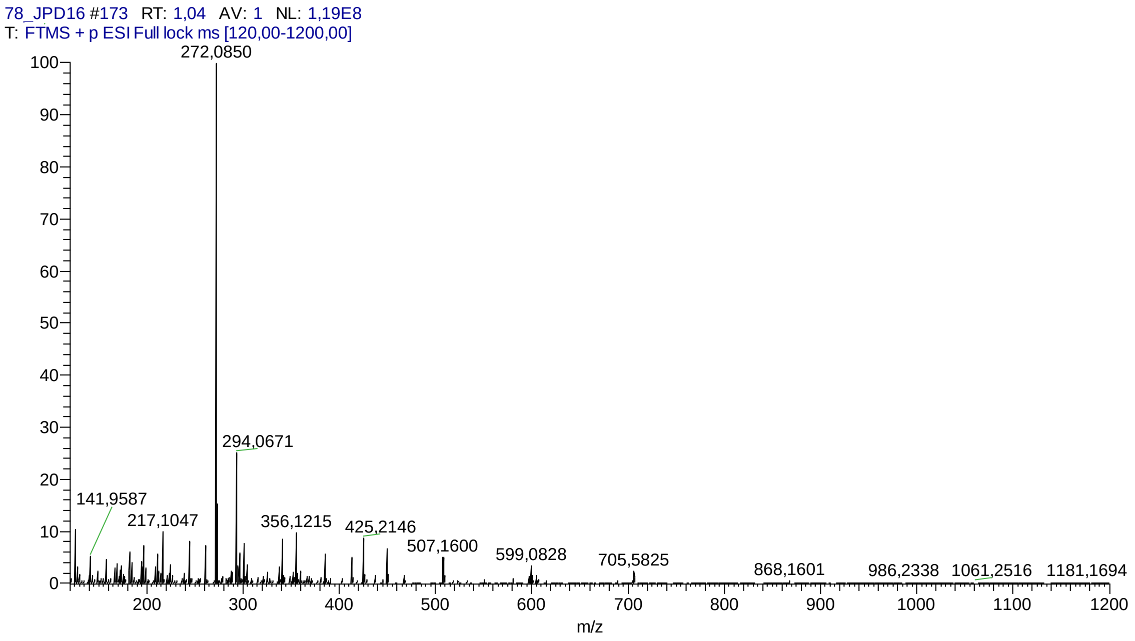

Figure S27: HRMS spectra of **2c**.

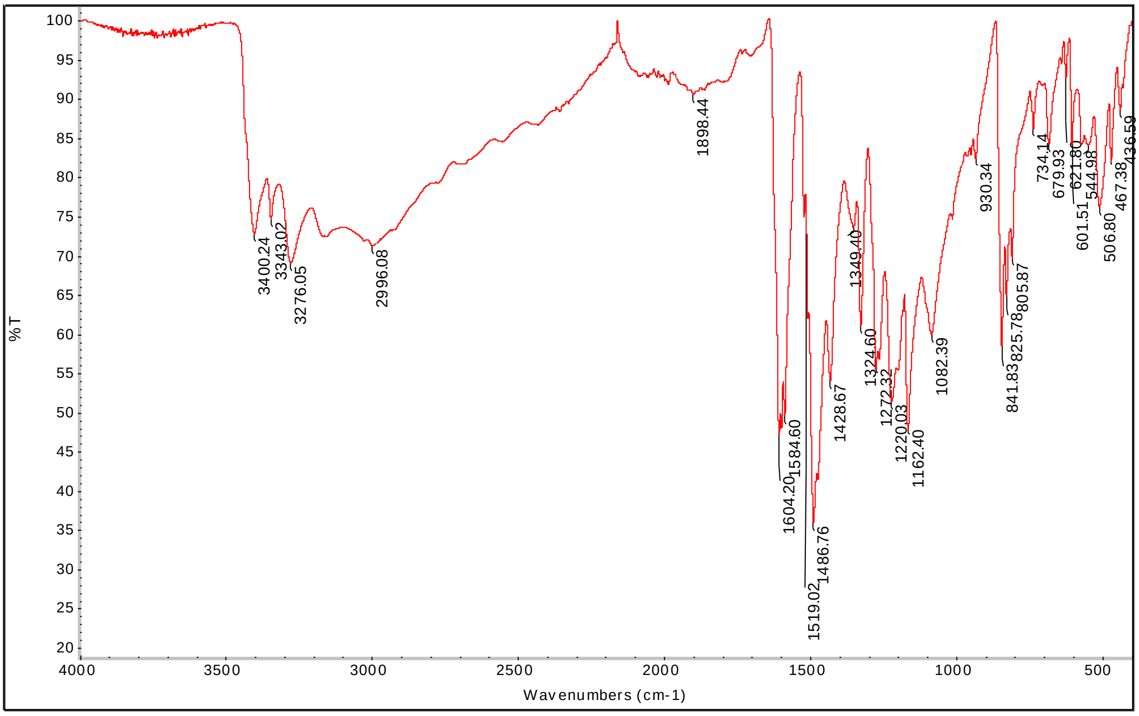

Figure S28: IR spectra of **2d**.

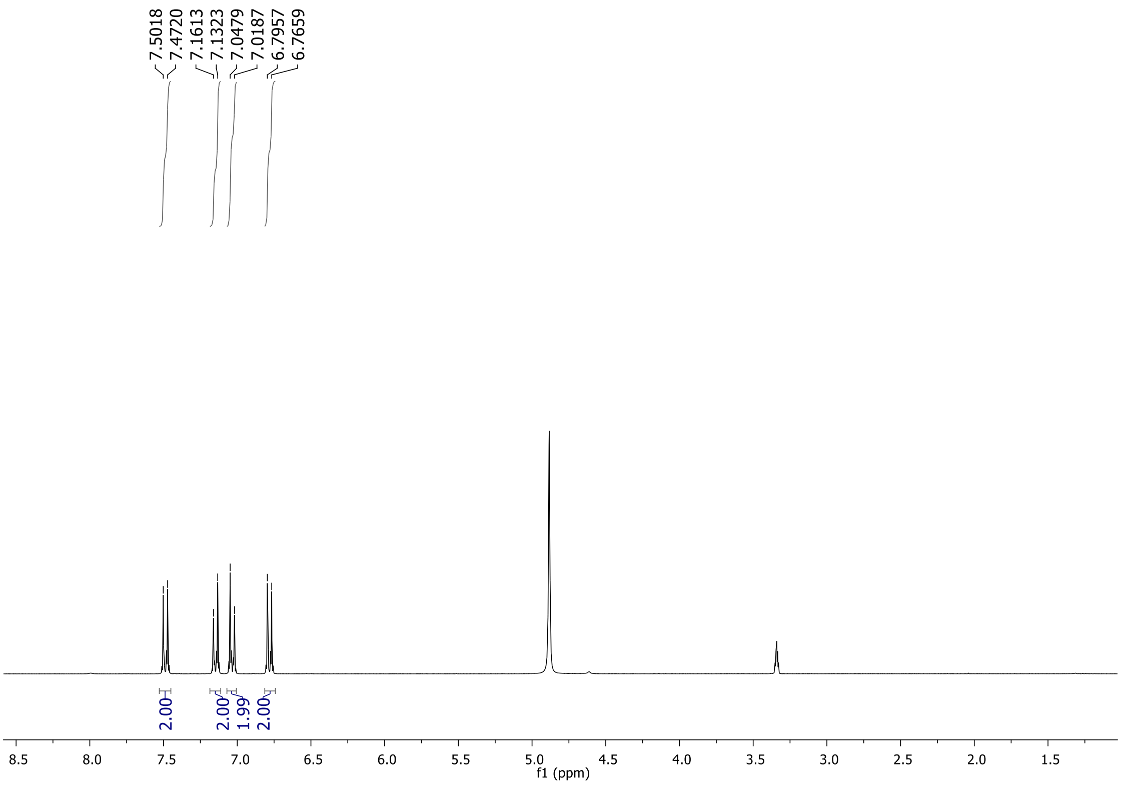

Figure S29: ^{1}H NMR spectra of **2d** (CD_3OD, 300 MHz).

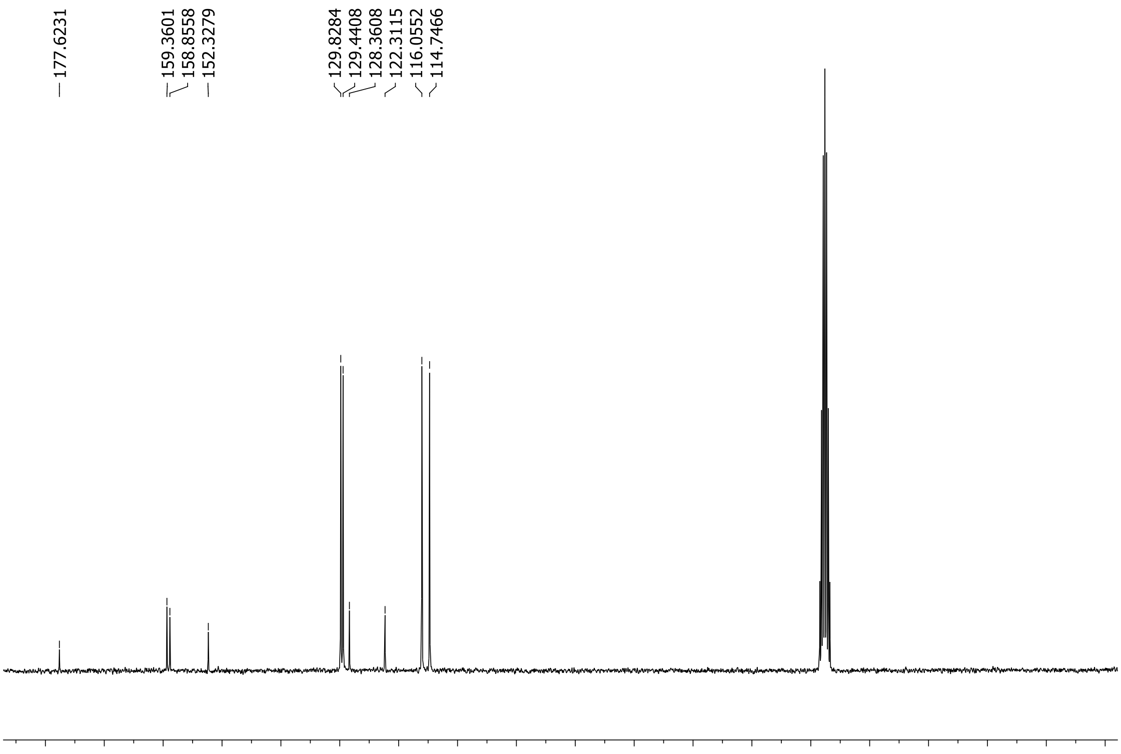

Figure S30: ^{13}C NMR spectra of **2d** (CD_3OD, 75 MHz).

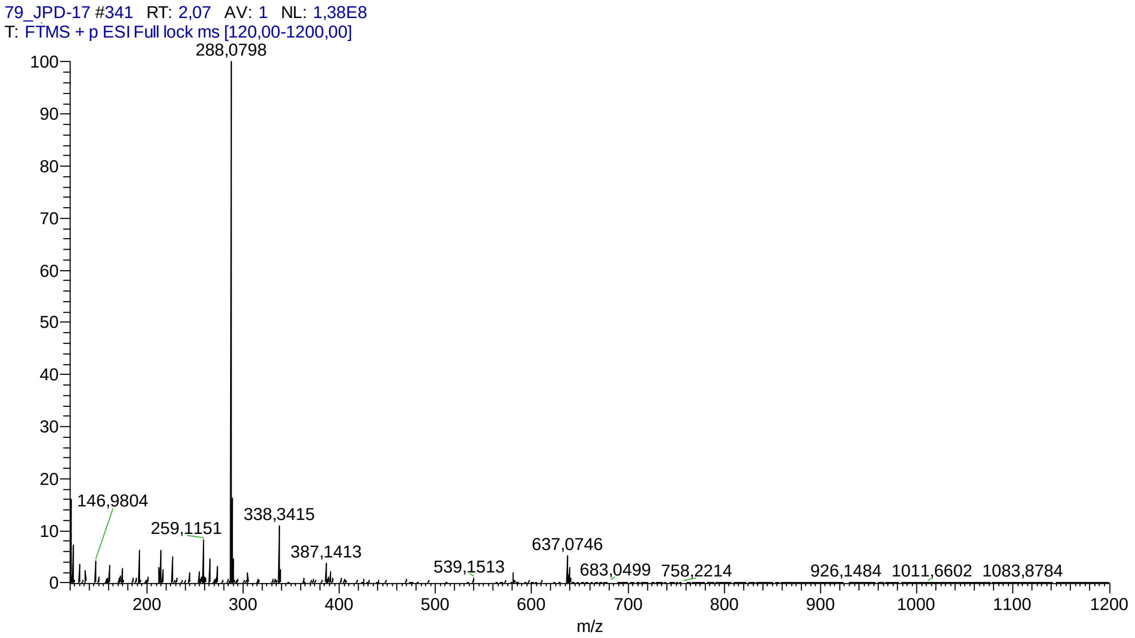

Figure S31: HRMS spectra of **2d**.

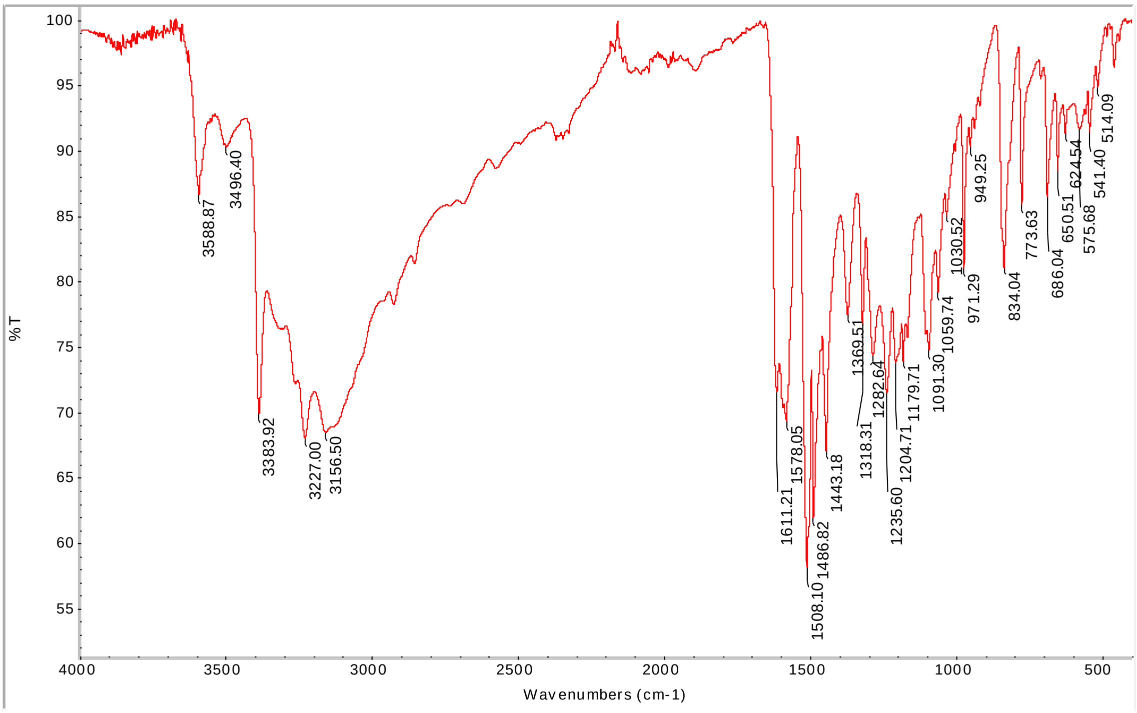

Figure S32: IR spectra of **2e**.

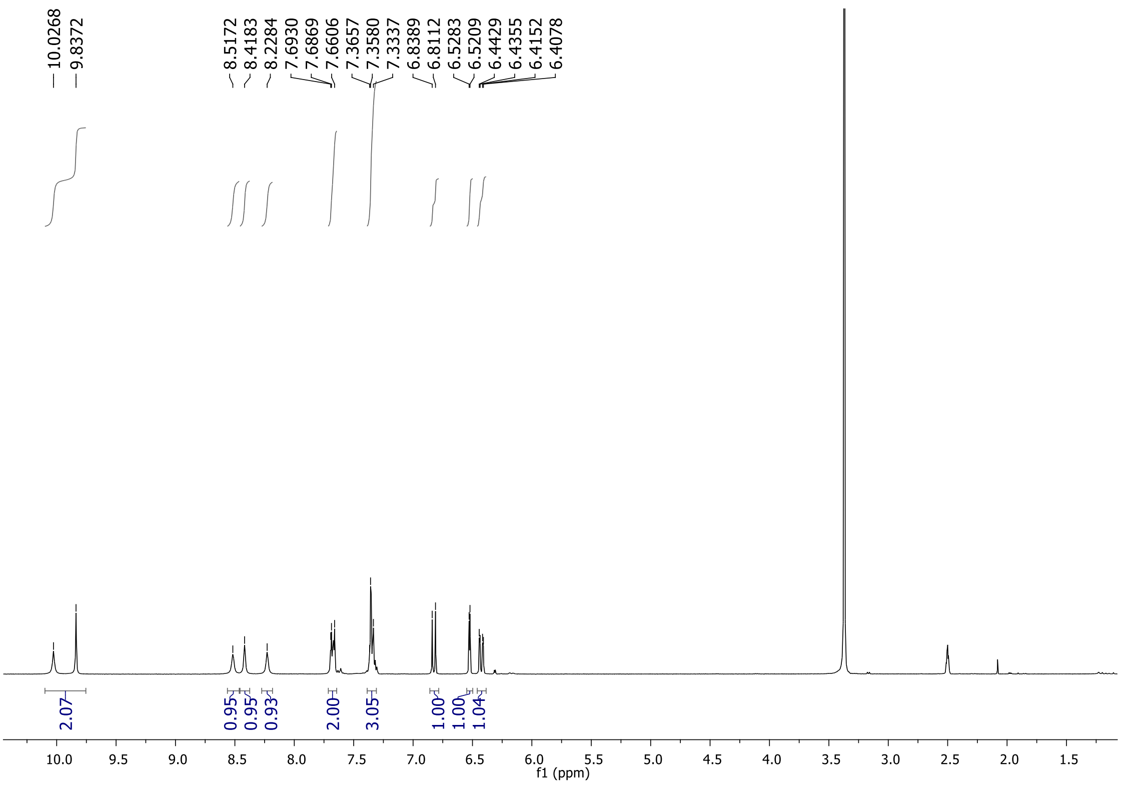

Figure S33: ^{1}H NMR spectra of **2e** (DMSO-d_6, 300 MHz).

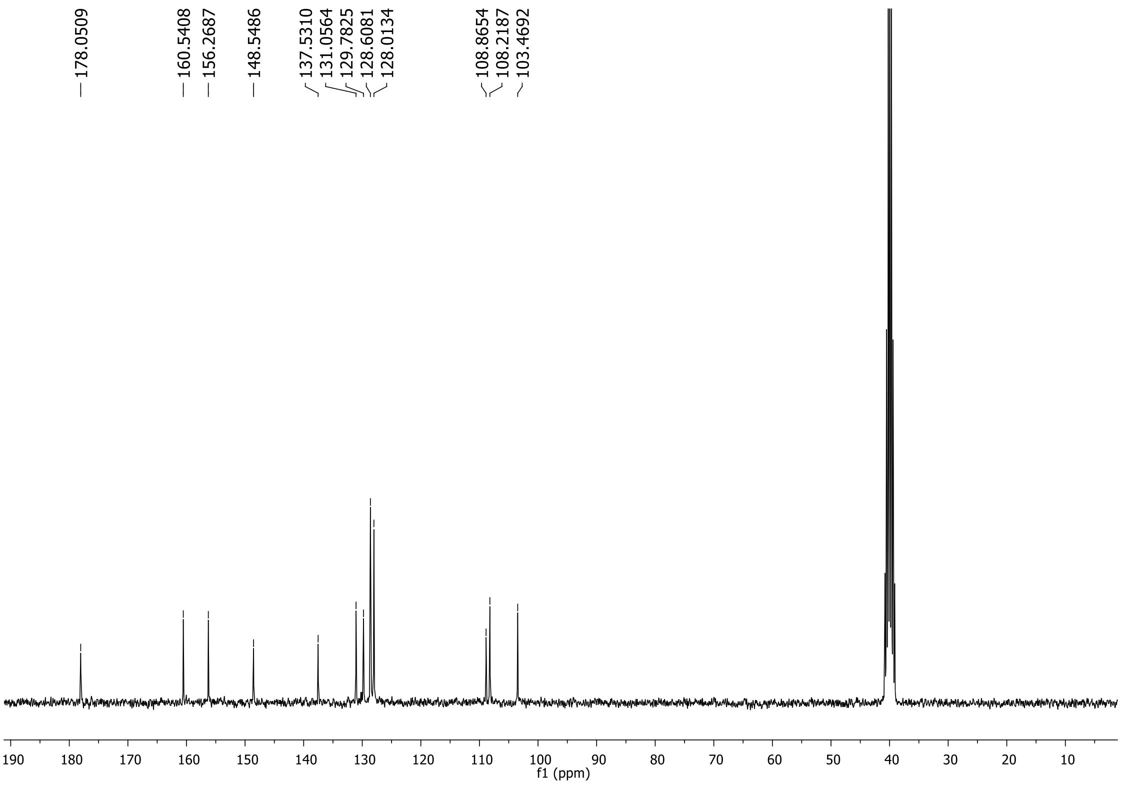

Figure S34: ^{13}C NMR spectra of **2e** (DMSO-d_6, 75 MHz).

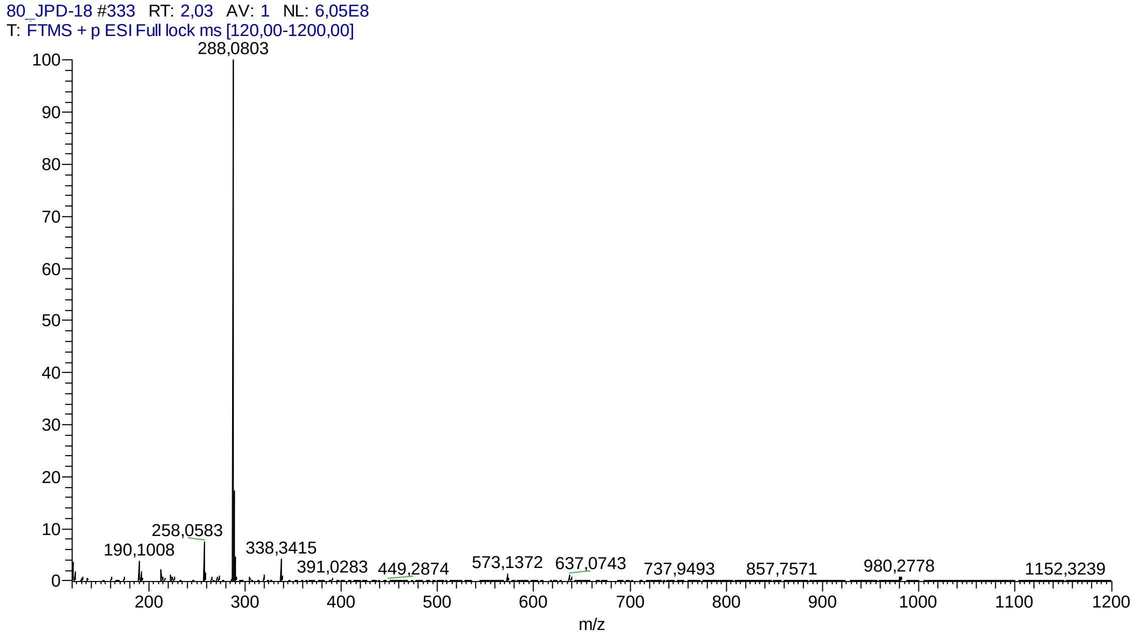

Figure S35: HRMS spectra of **2e**.

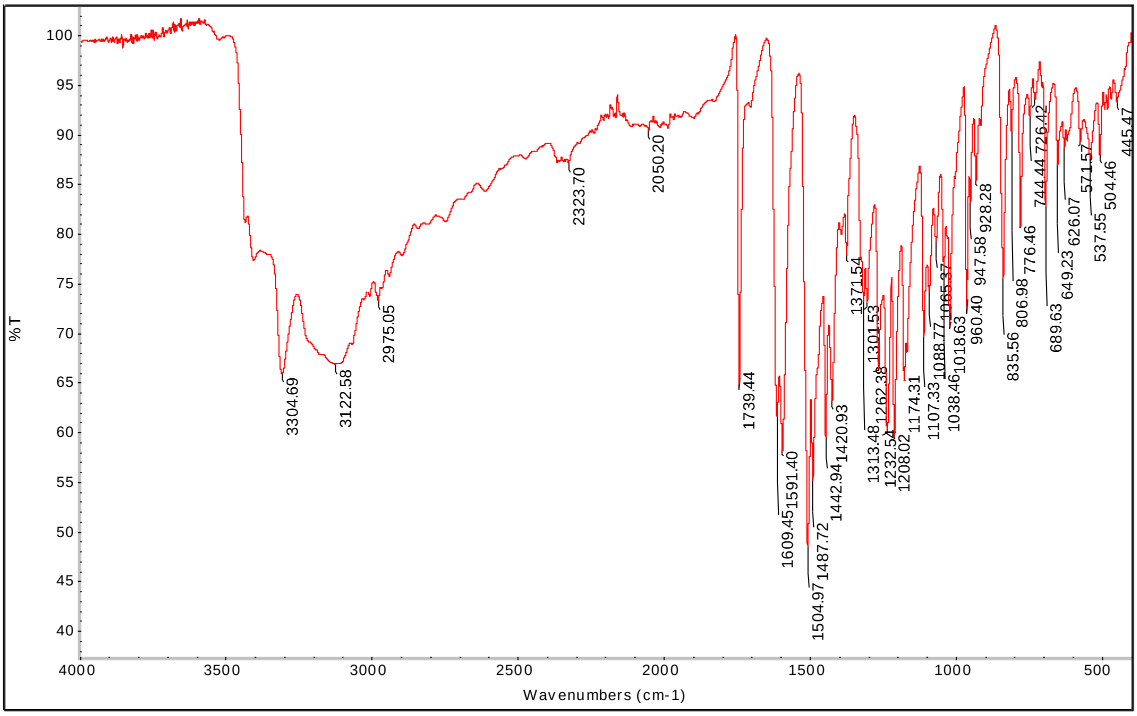

Figure S36: IR spectra of **2f**.

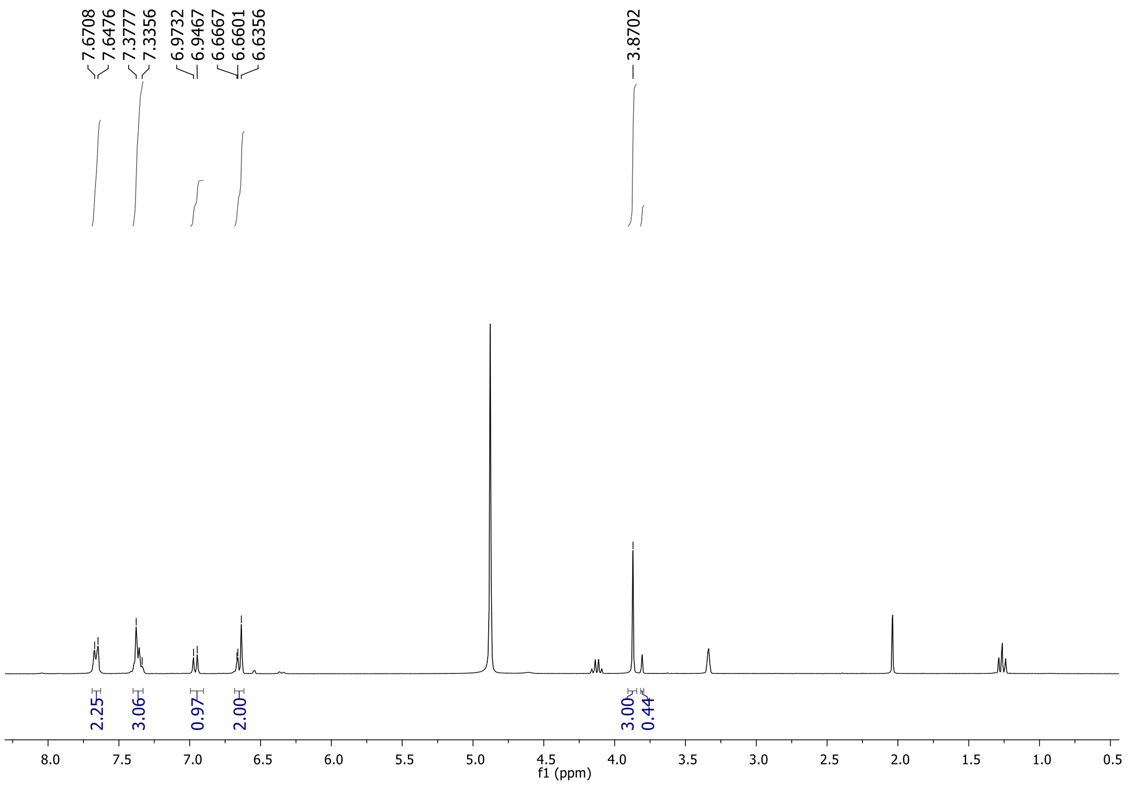

Figure S37: ^{1}H NMR spectra of **2f** (CD_3OD, 300 MHz).

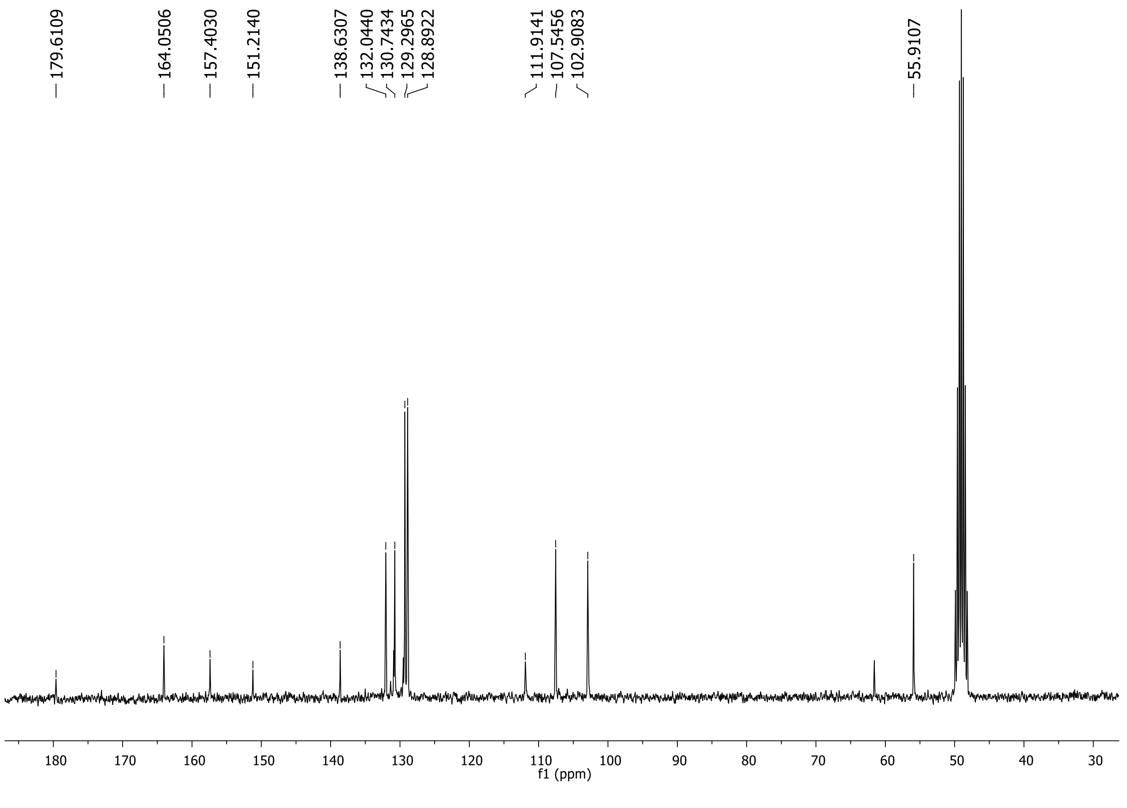

Figure S38: ^{13}C NMR spectra of **2f** (CD_3OD, 75 MHz).

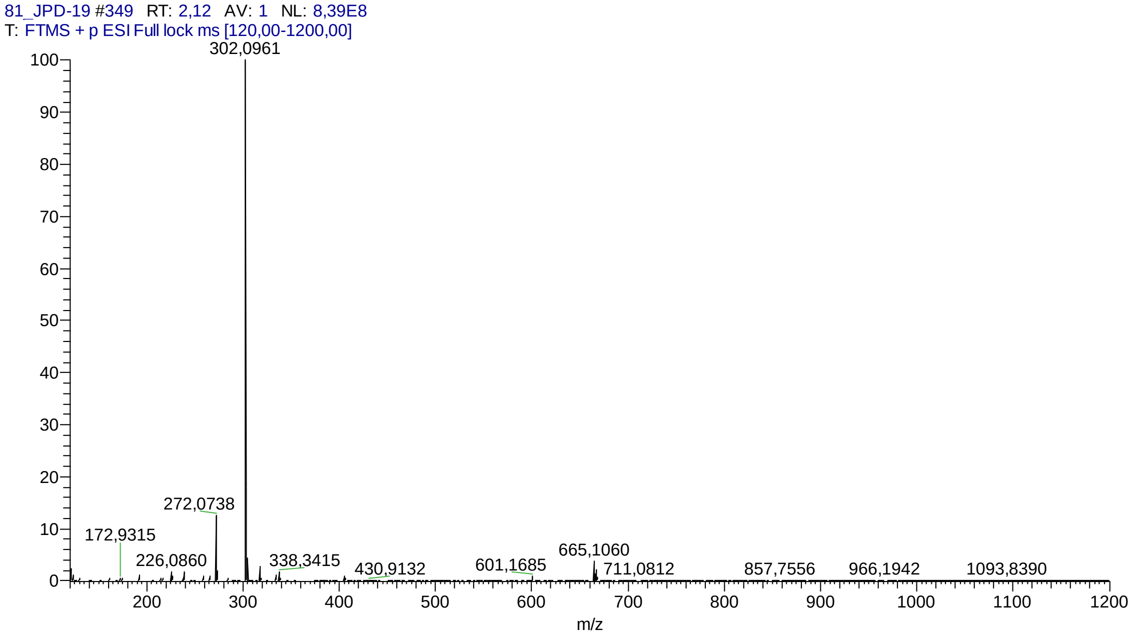

Figure S39: HRMS spectra of **2f**.

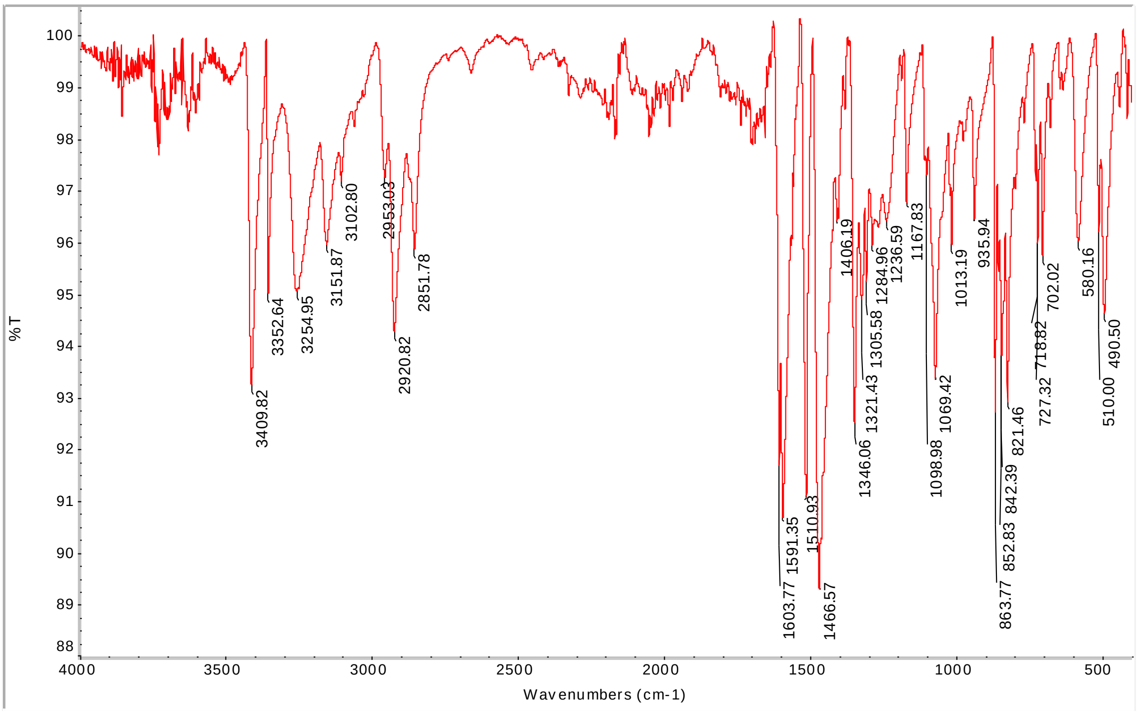

Figure S40: IR spectra of **2g**.

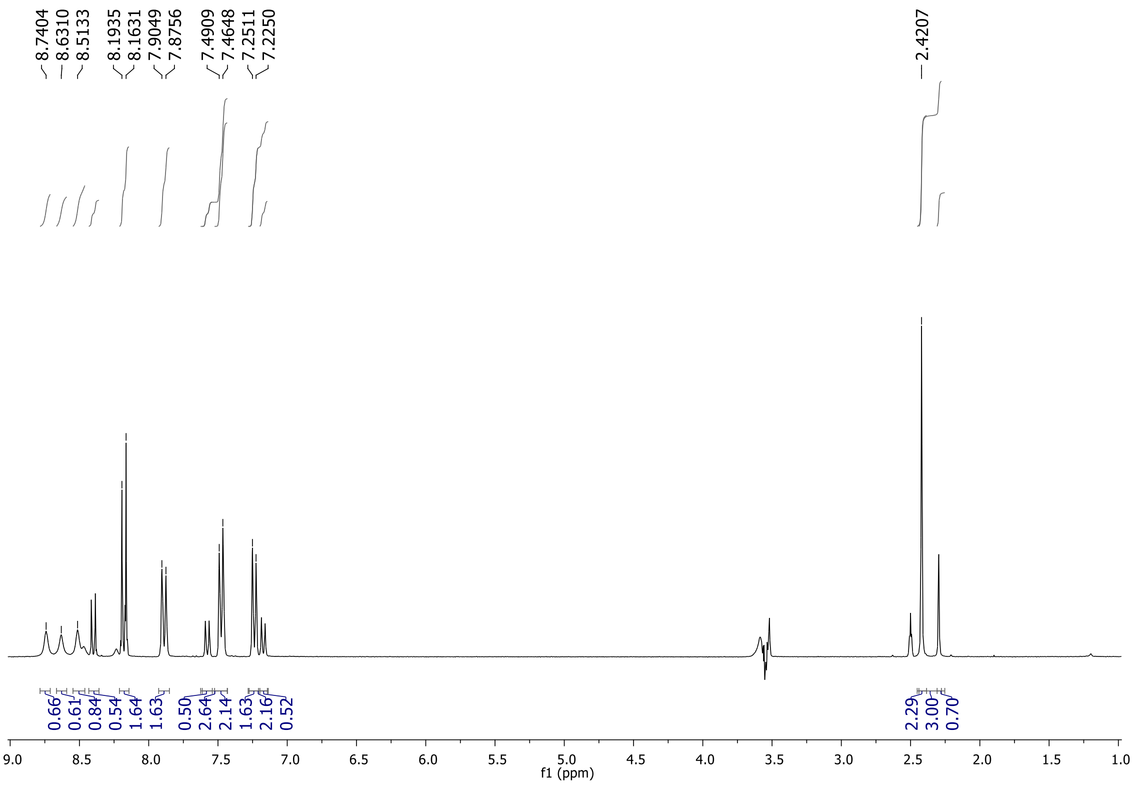

Figure S41: ^{1}H NMR spectra of **2g** (DMSO-d_6, 300 MHz).

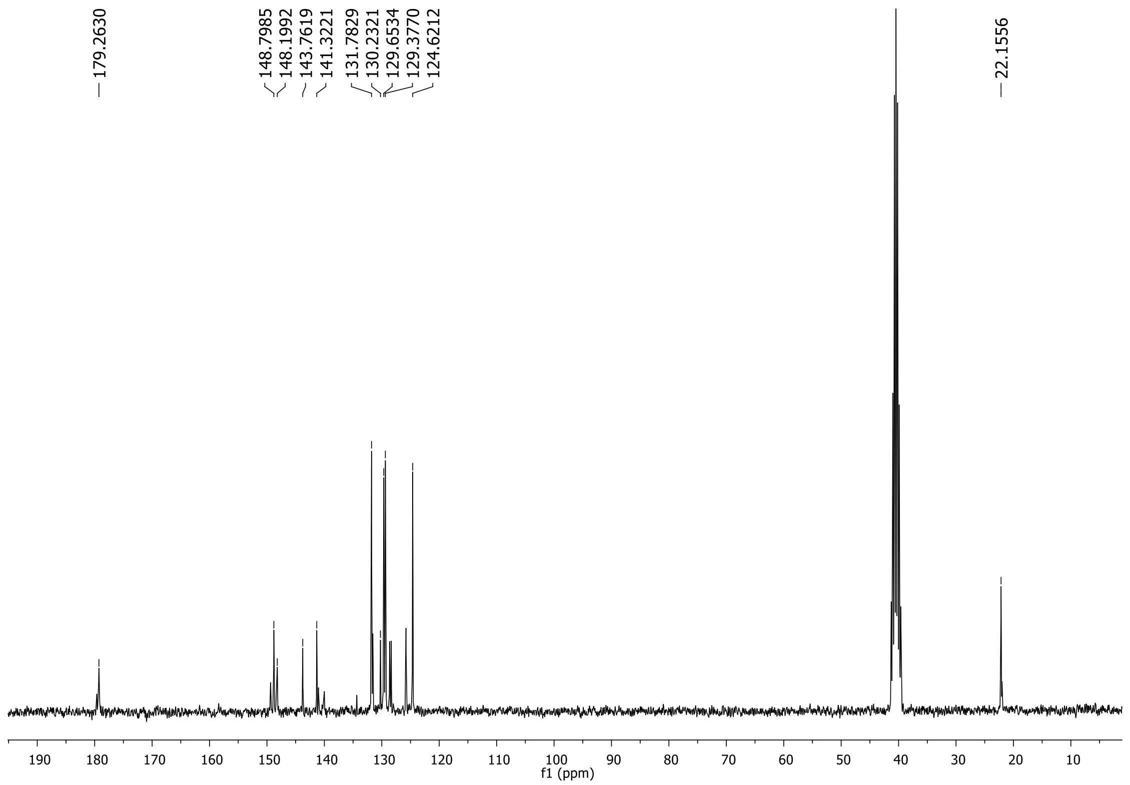

Figure S42: ^{13}C NMR spectra of **2g** (DMSO-d_6, 75 MHz).

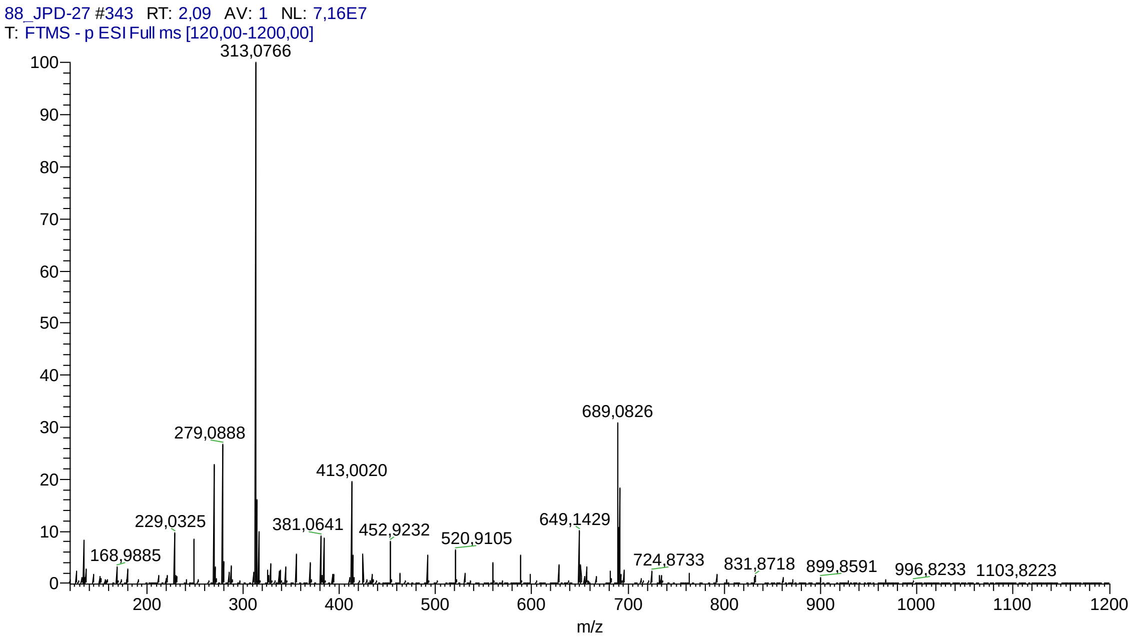

Figure S43: HRMS spectra of **2g**.

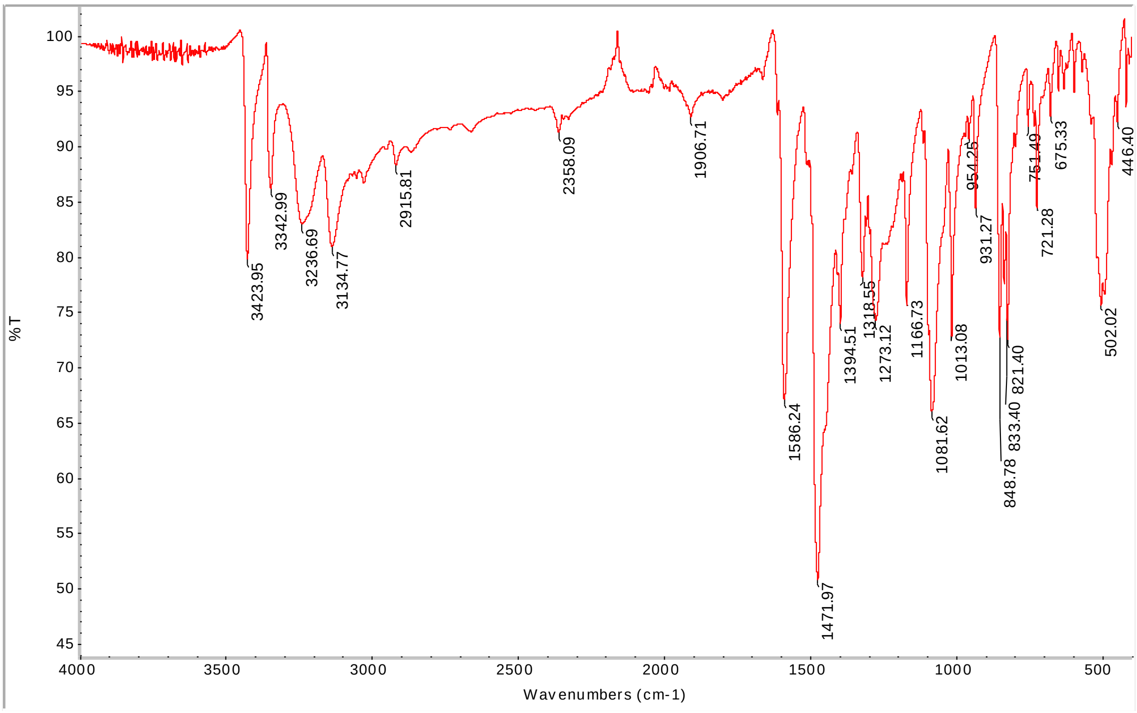

Figure S44: IR spectra of **2h**.

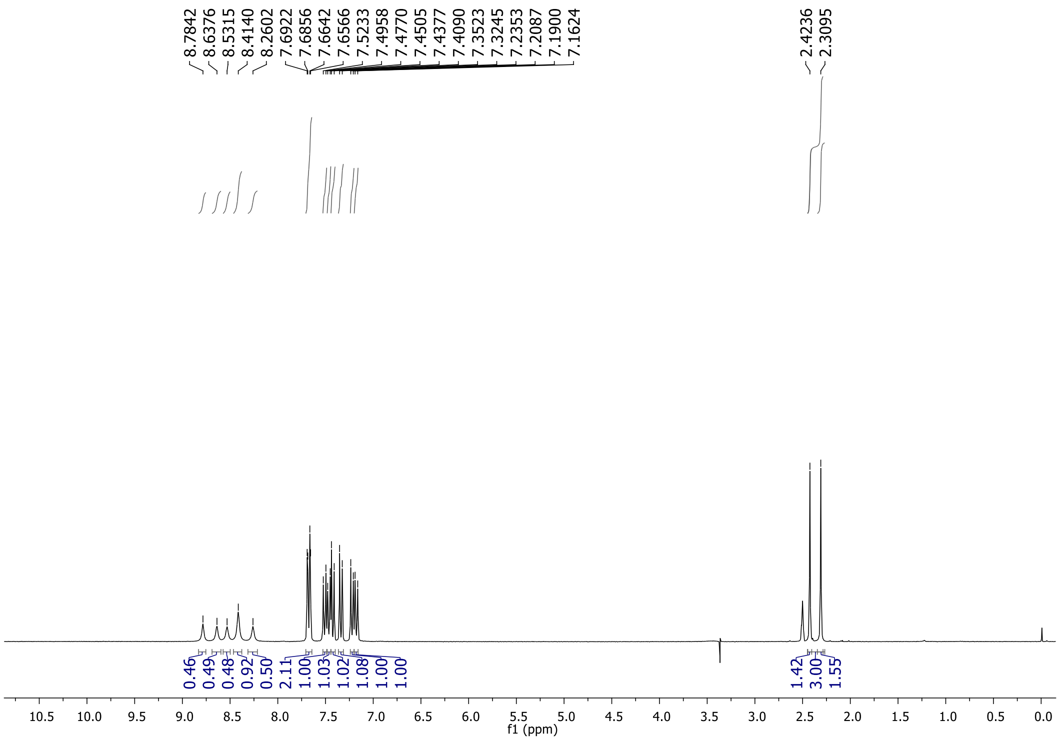

Figure S45: ^{1}H NMR spectra of **2h** (DMSO-d_6, 300 MHz).

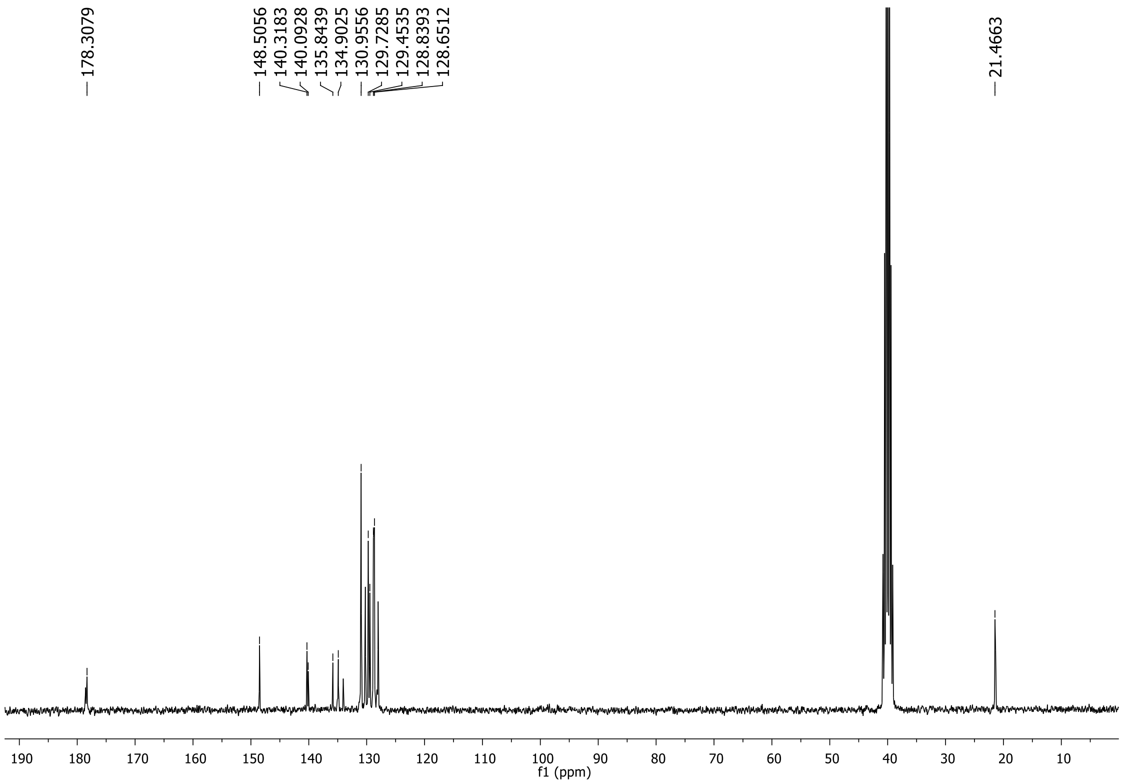

Figure S46: ^{13}C NMR spectra of **2h** (DMSO-d_6, 75 MHz).

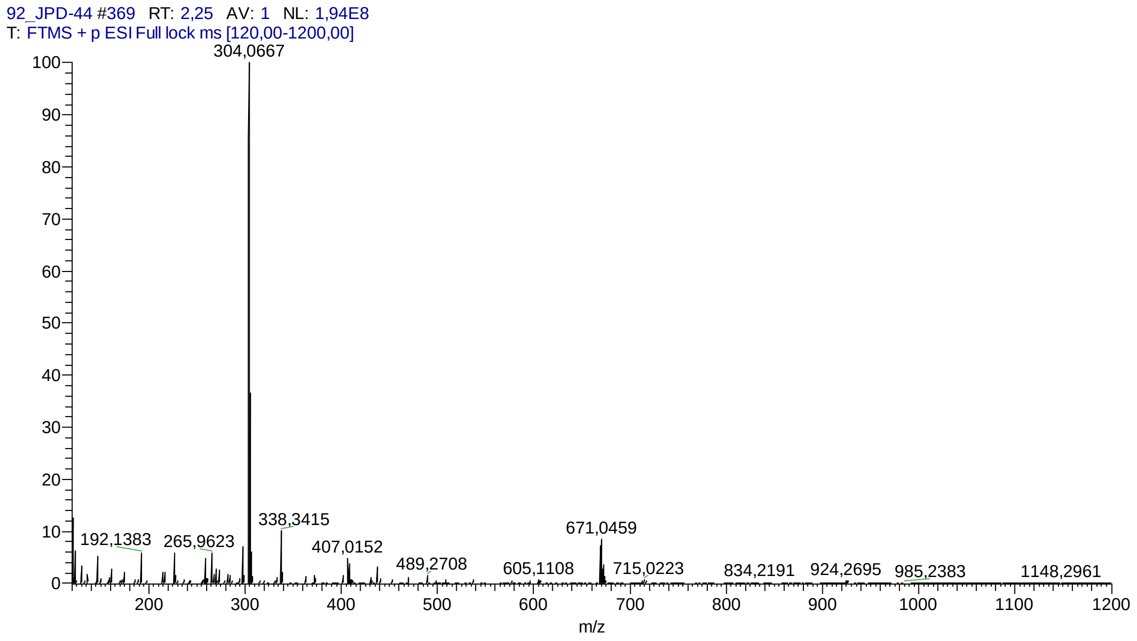

Figure S47: HRMS spectra of **2h**.

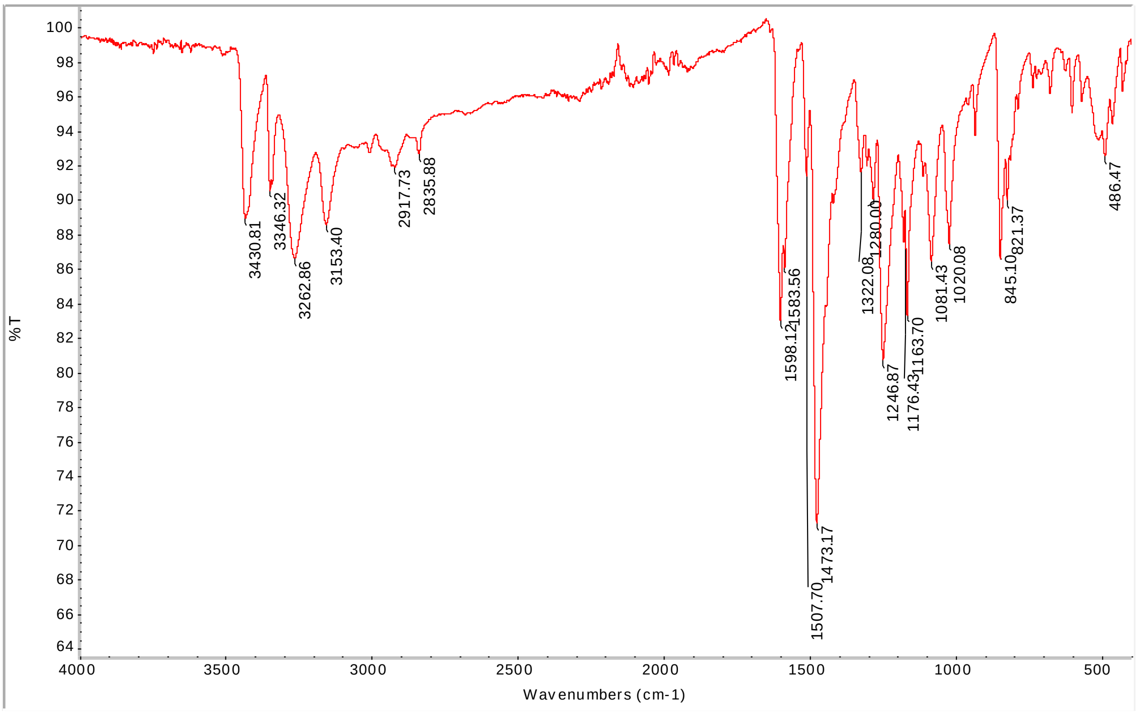

Figure S48: IR spectra of **2i**.

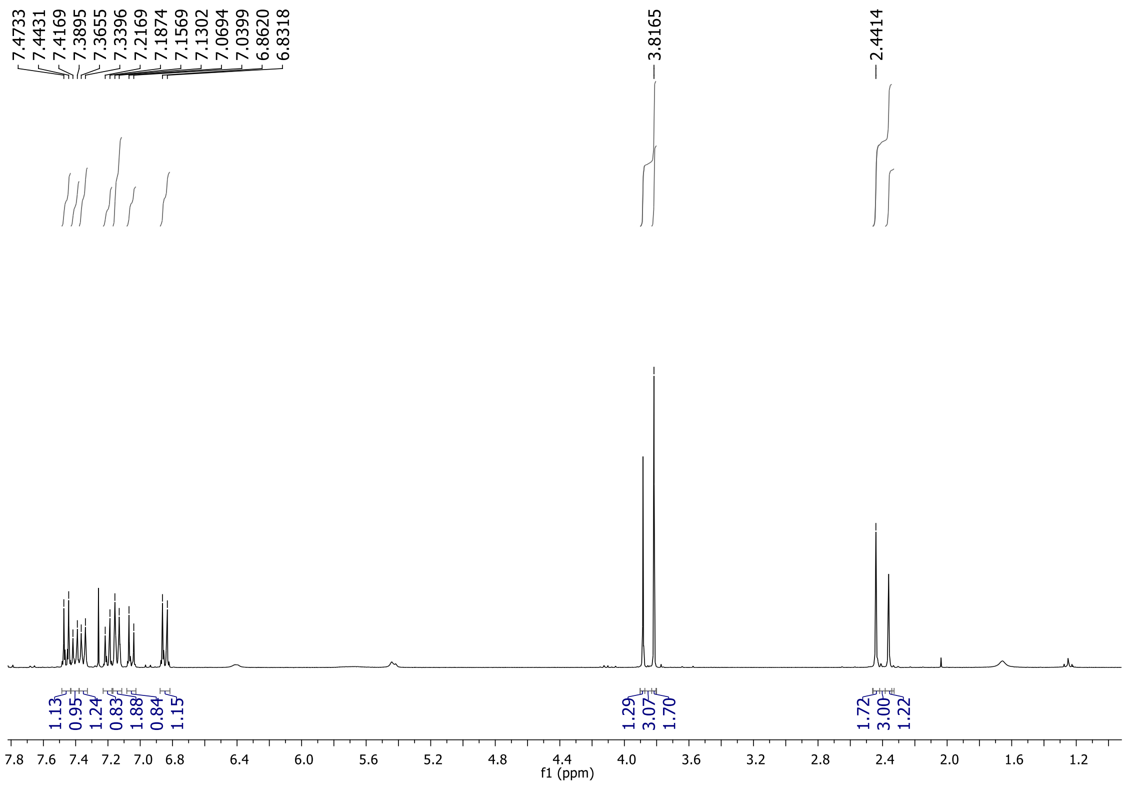

Figure S49: ^{1}H NMR spectra of **2i** ($CDCl_3$, 300 MHz).

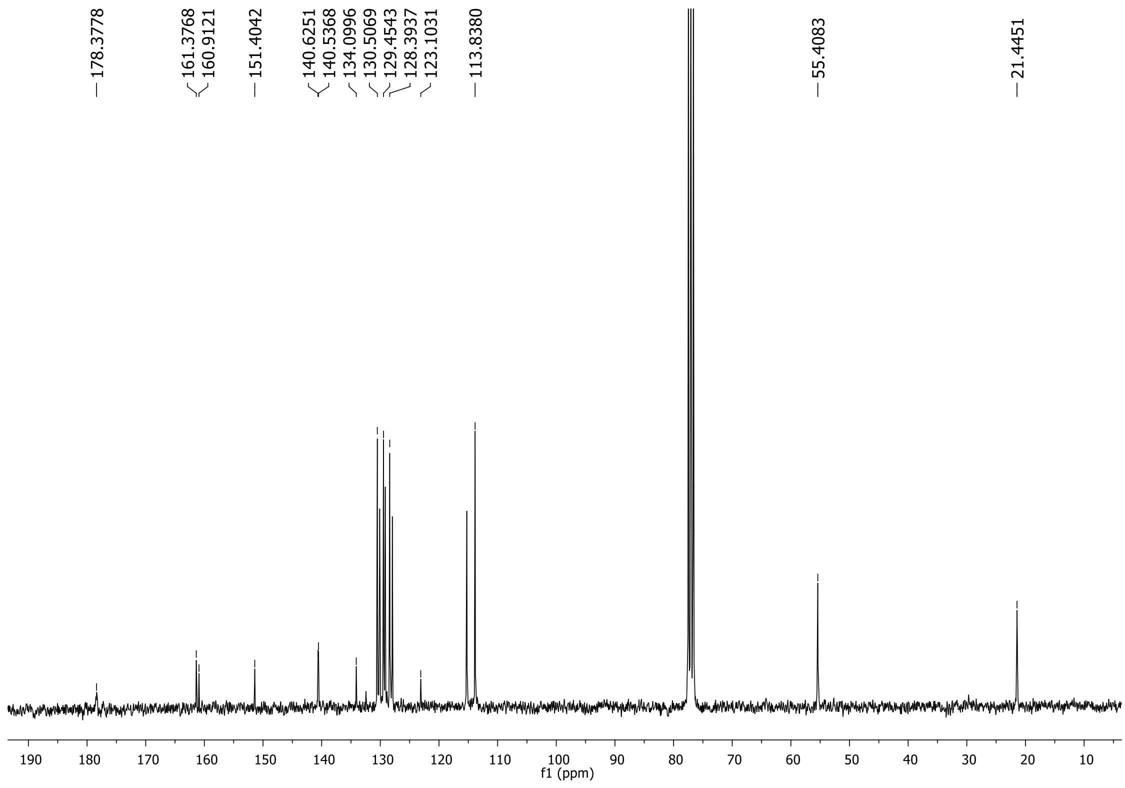

Figure S50: ^{13}C NMR spectra of **2i** ($CDCl_3$, 75 MHz).

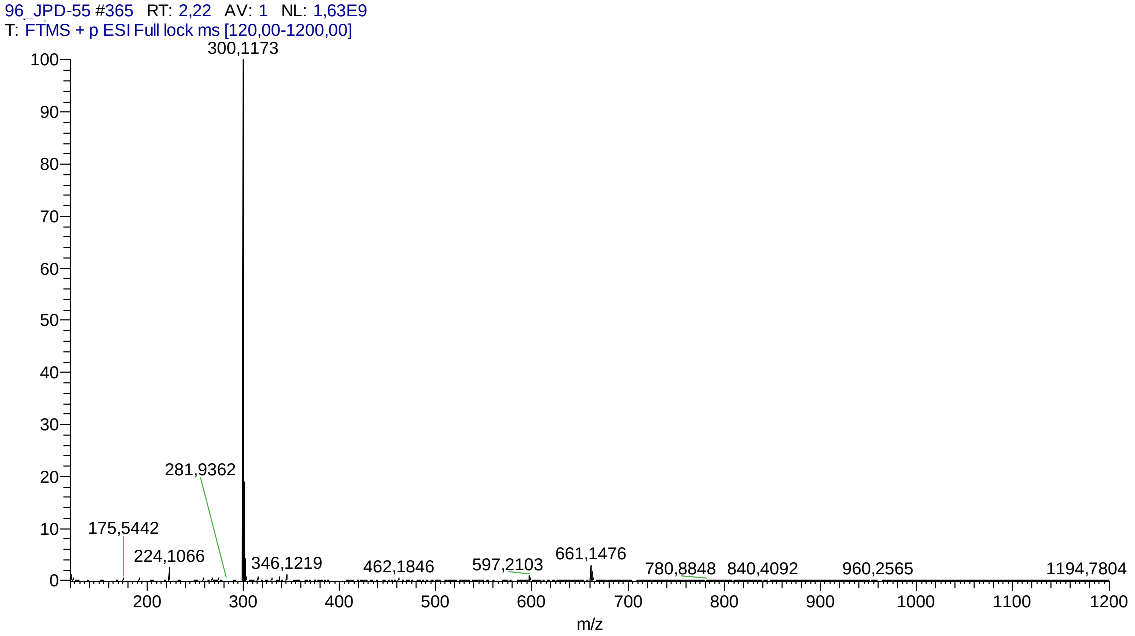

Figure S51: HRMS spectra of **2i**.

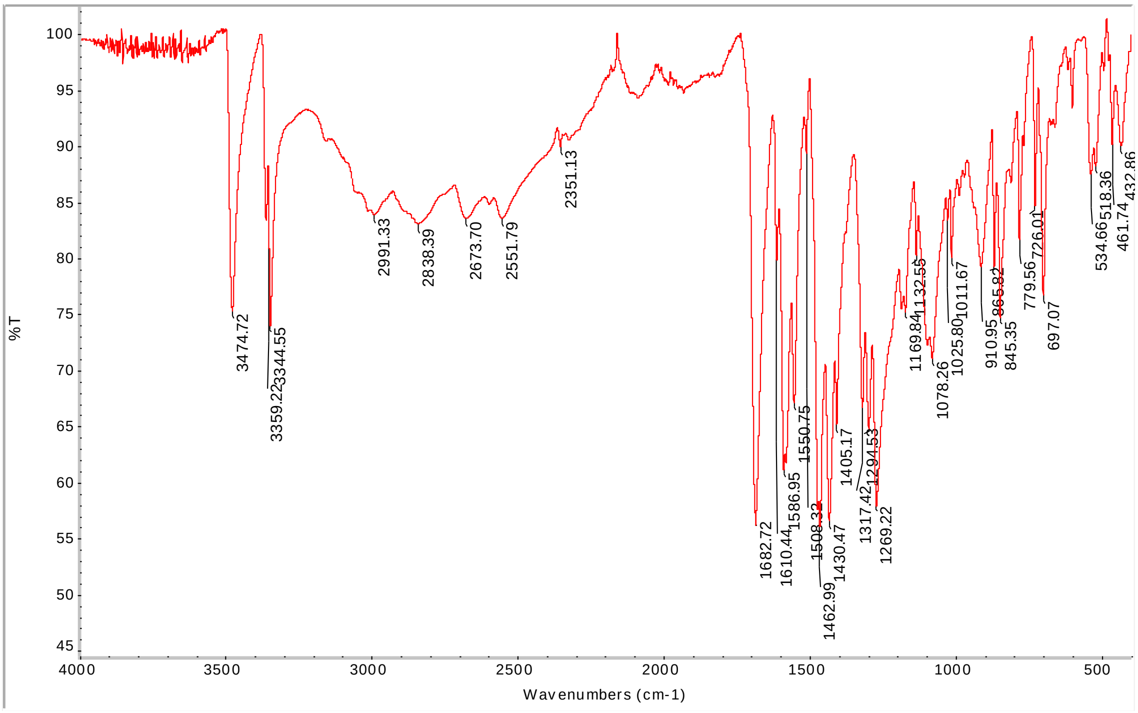

Figure S52: IR spectra of **2j**.

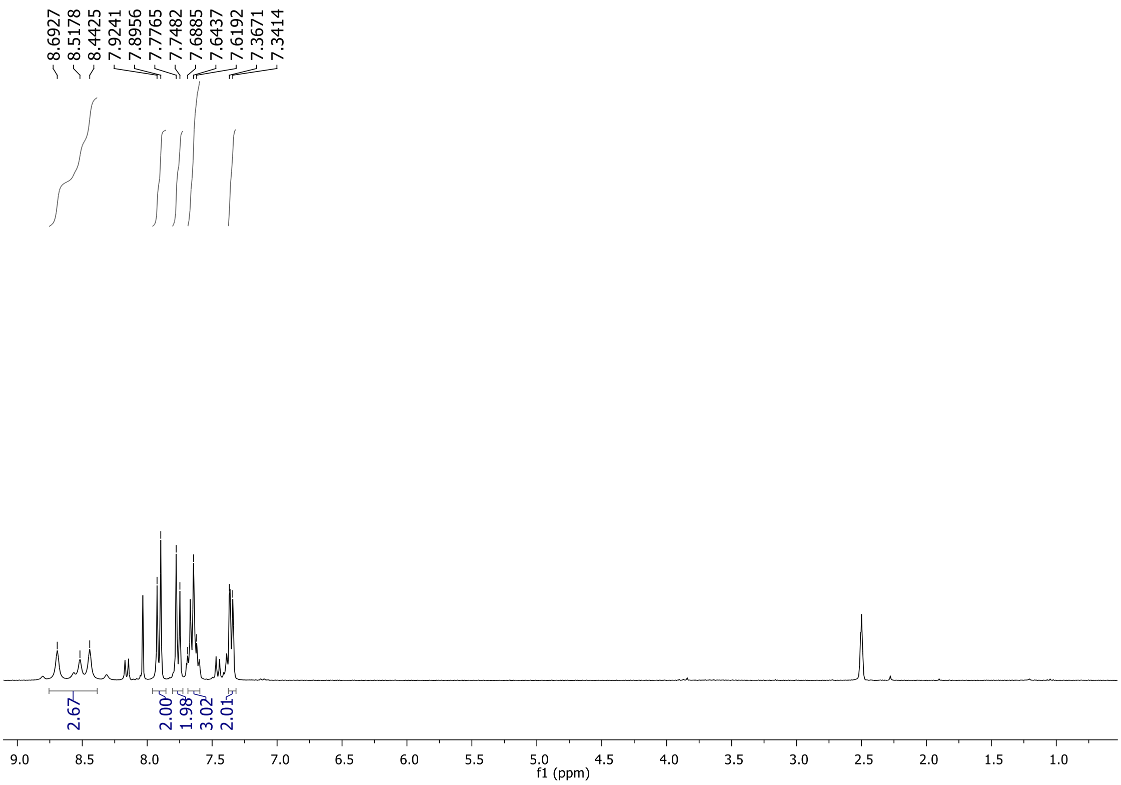

Figure S53: ^{1}H NMR spectra of **2j** (DMSO-d_6, 300 MHz).

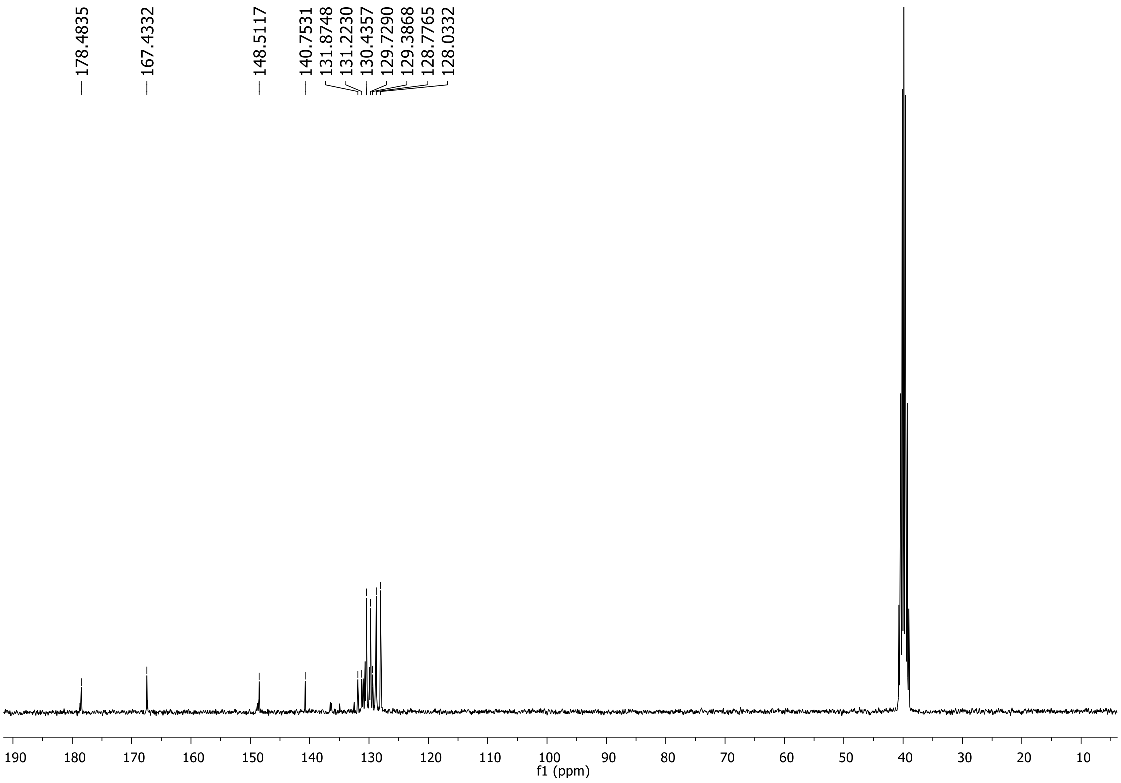

Figure S54: ^{13}C NMR spectra of **2j** (DMSO-d_6, 75 MHz).

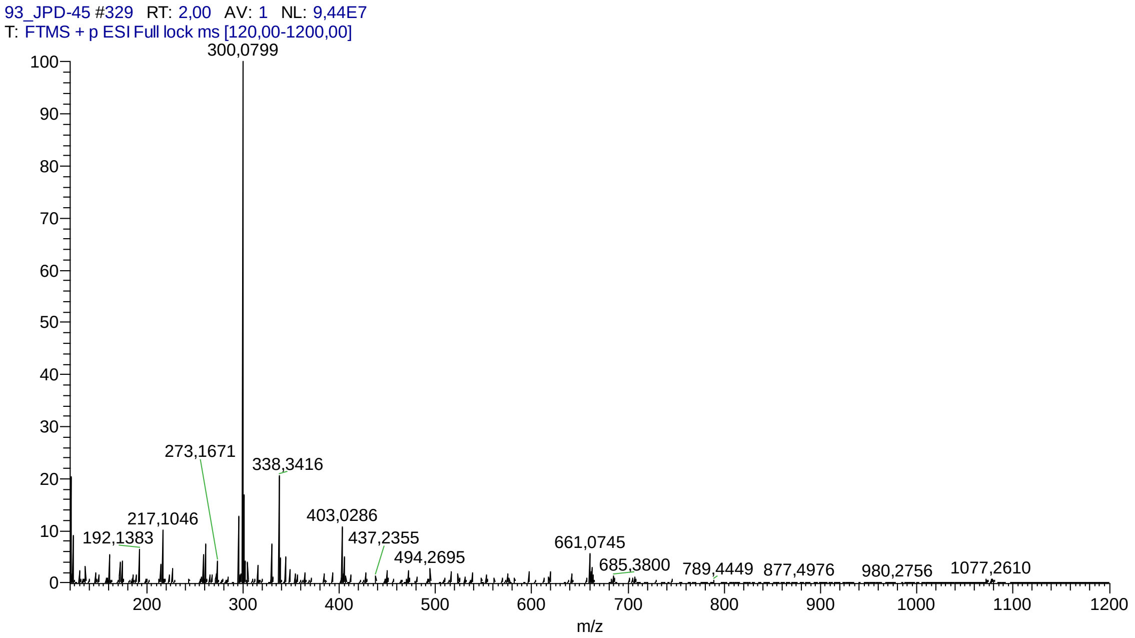

Figure S55: HRMS spectra of **2j**.

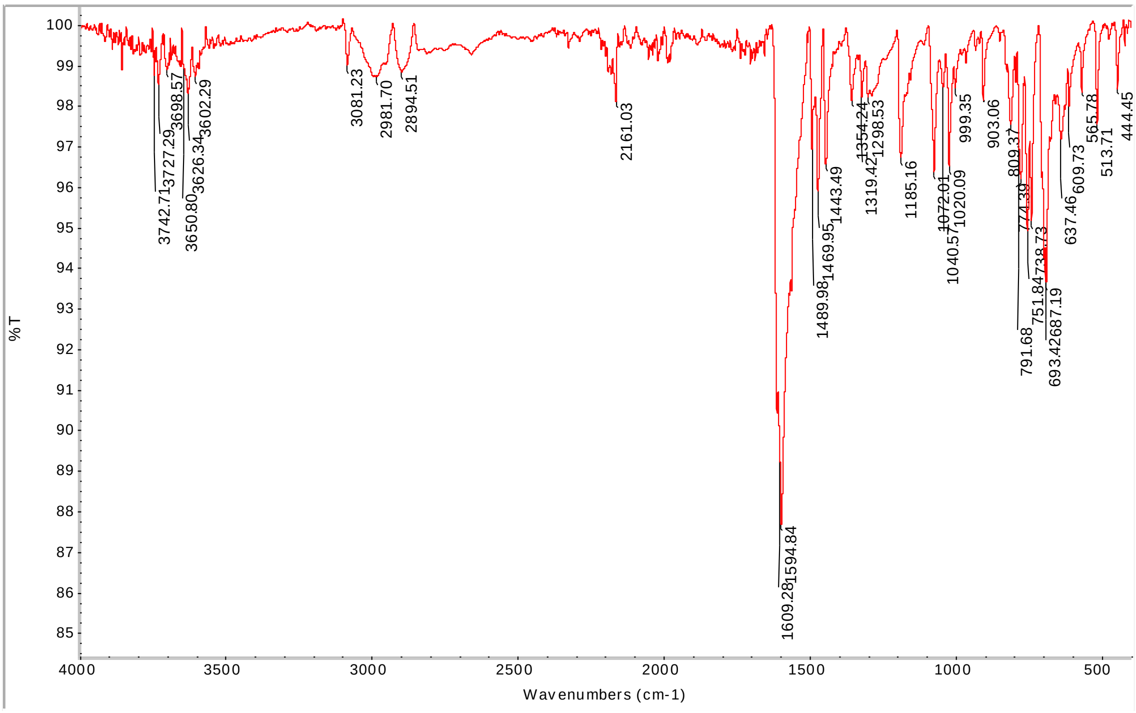

Figure S56: IR spectra of **3a**.

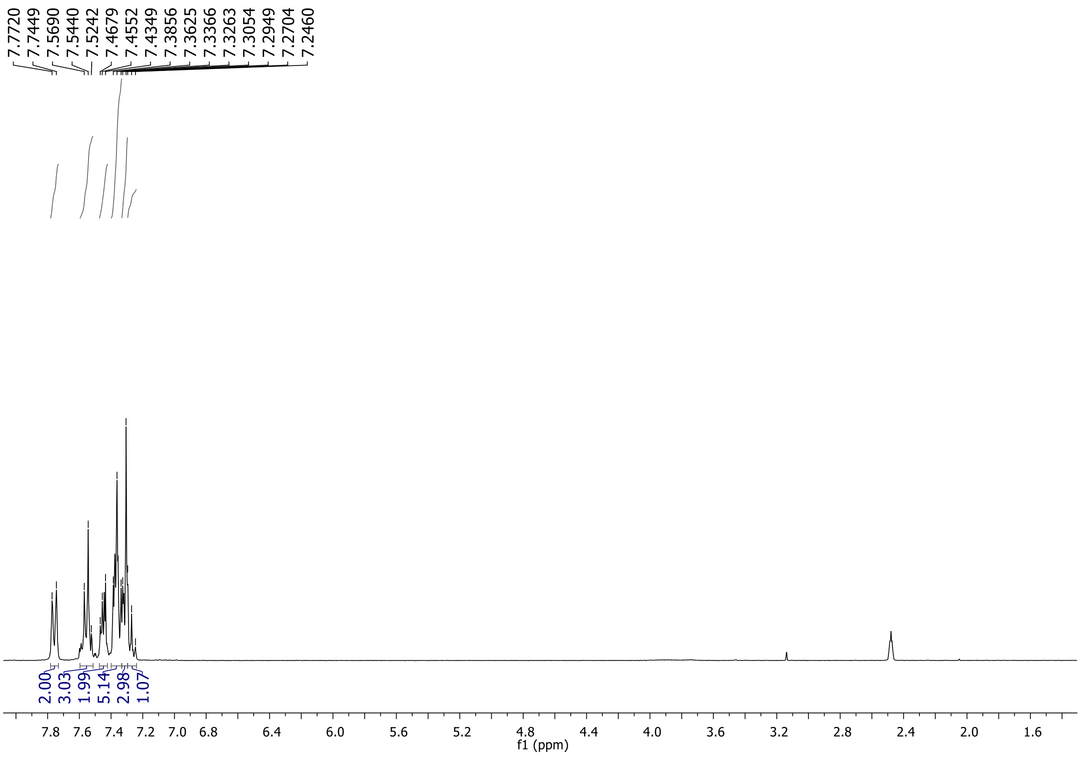

Figure S57: ^{1}H NMR spectra of **3a** (DMSO-d_6, 300 MHz).

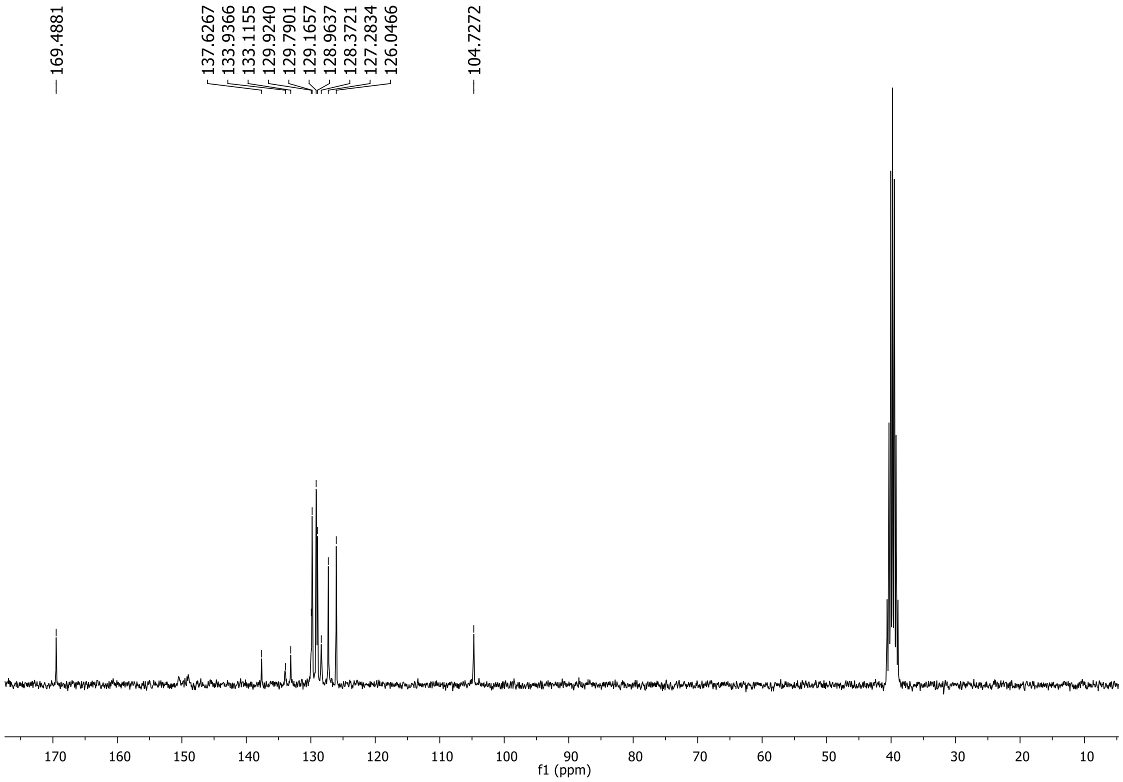

Figure S58: ^{13}C NMR spectra of **3a** (DMSO-d_6, 75 MHz).

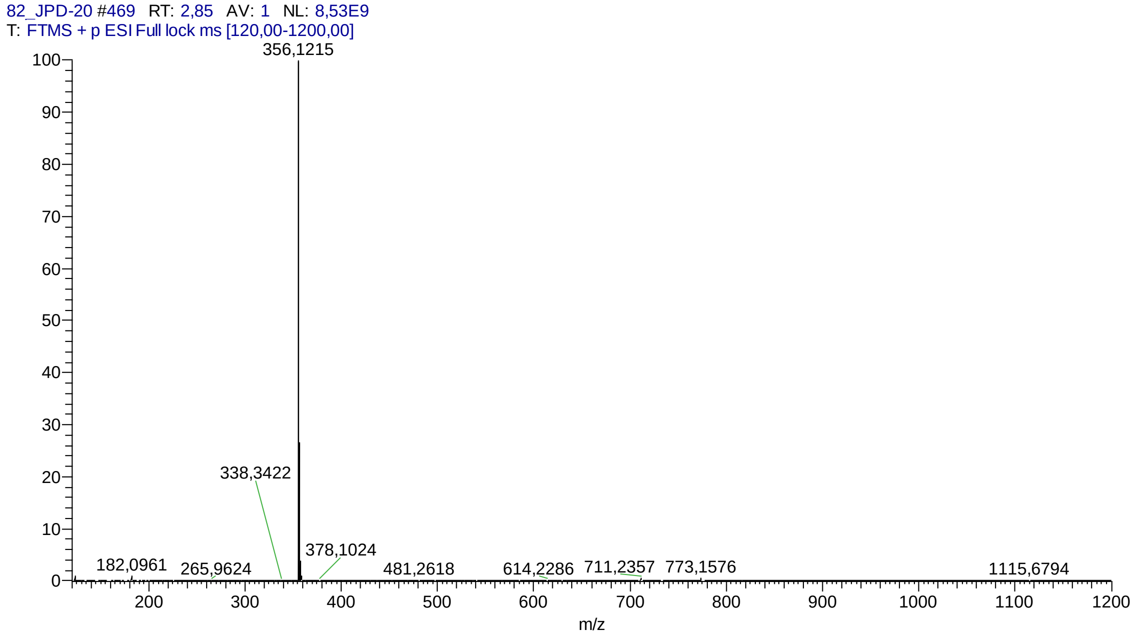

Figure S59: HRMS spectra of **3a**.

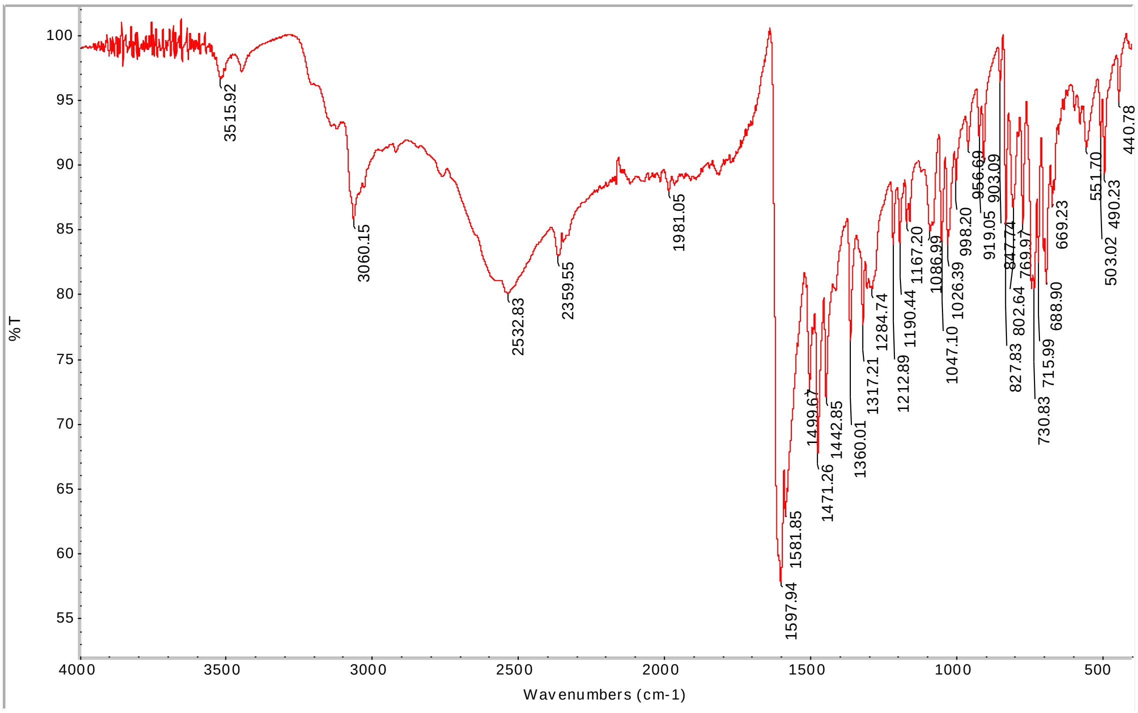

Figure S60: IR spectra of **3b**.

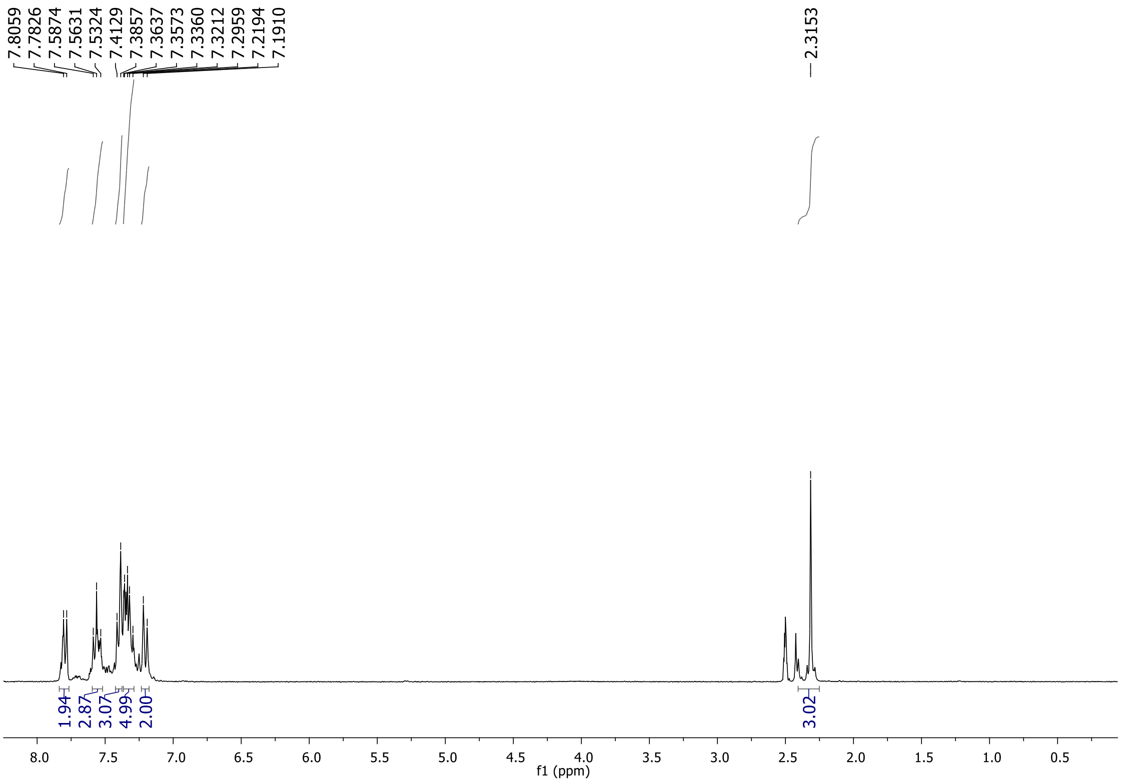

Figure S61: ^{1}H NMR spectra of **3b** (DMSO-d_6, 300 MHz).

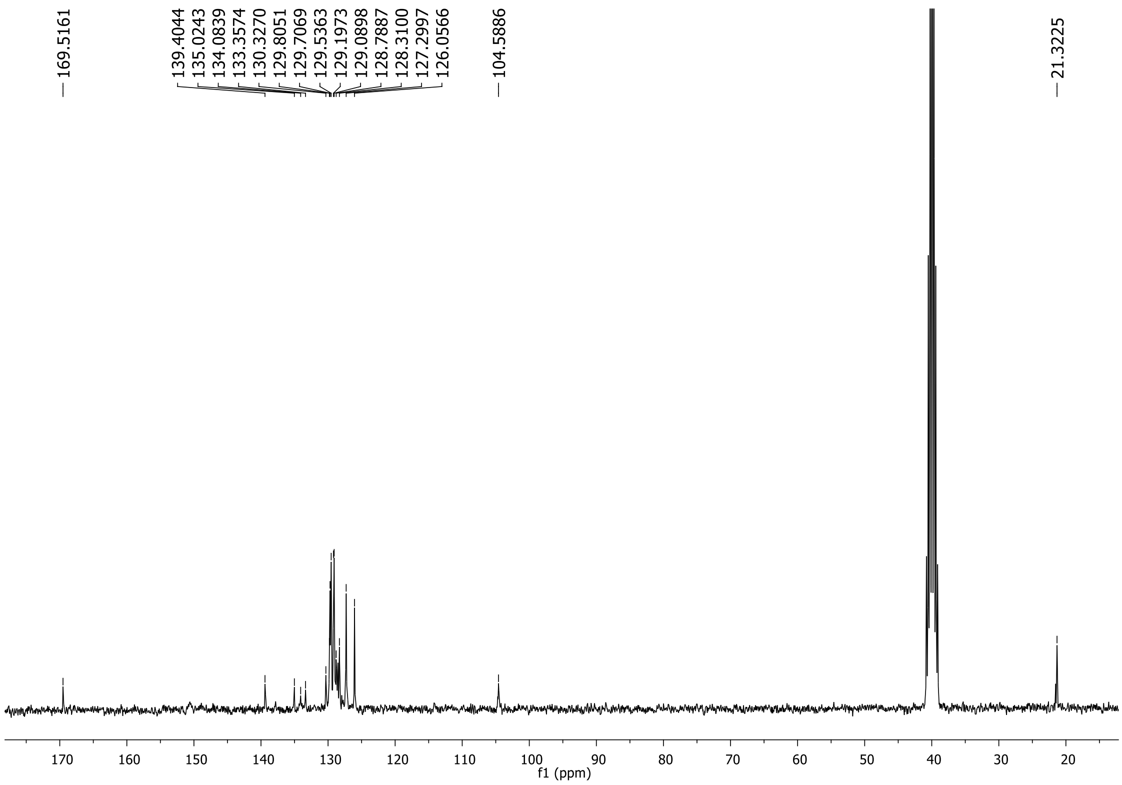

Figure S62: ^{13}C NMR spectra of **3b** (DMSO-d_6, 75 MHz).

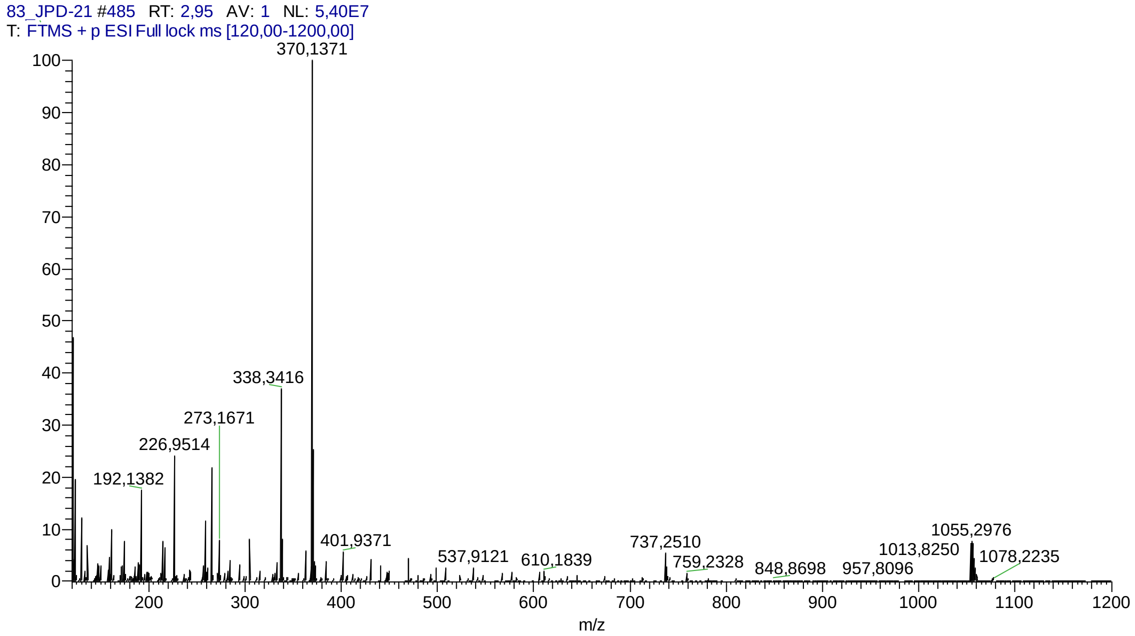

Figure S63: HRMS spectra of **3b**.

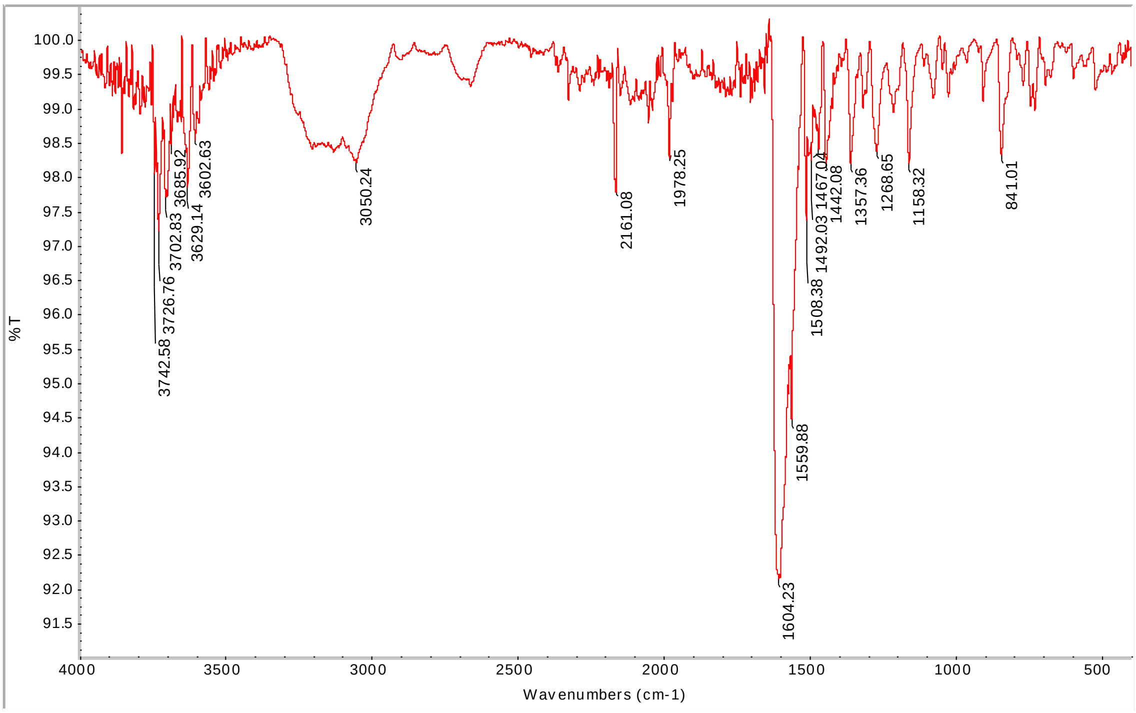

Figure S64: IR spectra of **3c**.

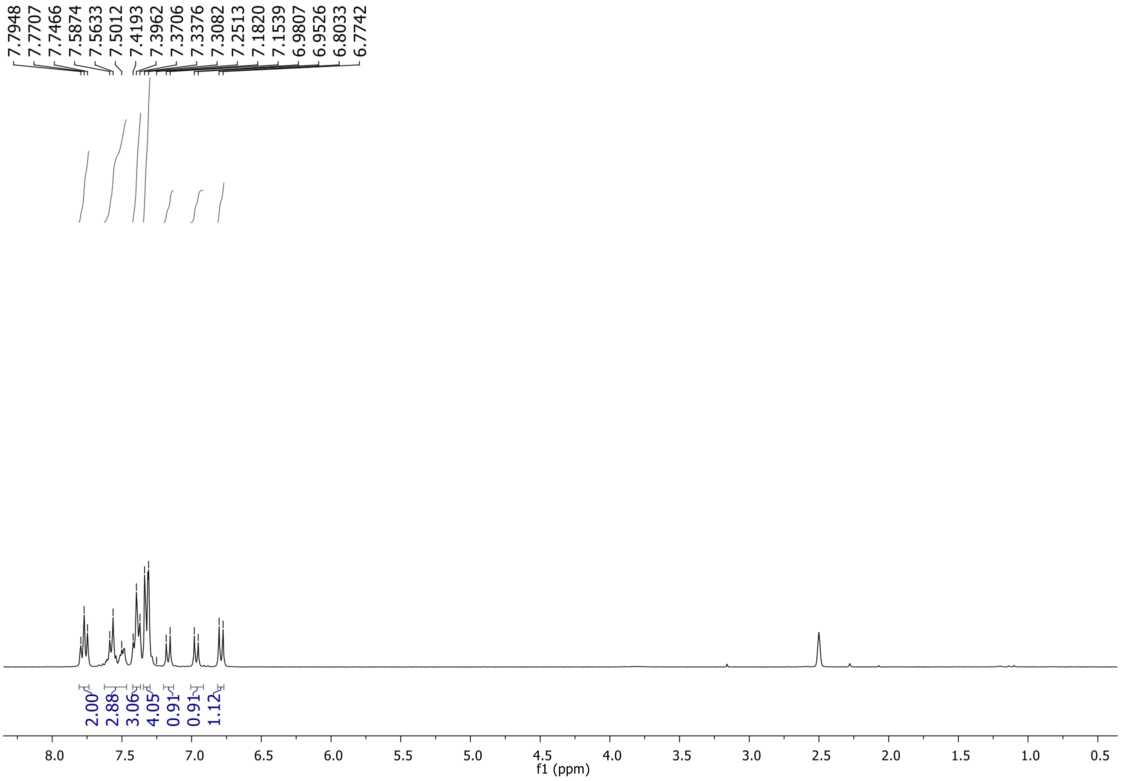

Figure S65: ^{1}H NMR spectra of **3c** (DMSO-d_6, 300 MHz).

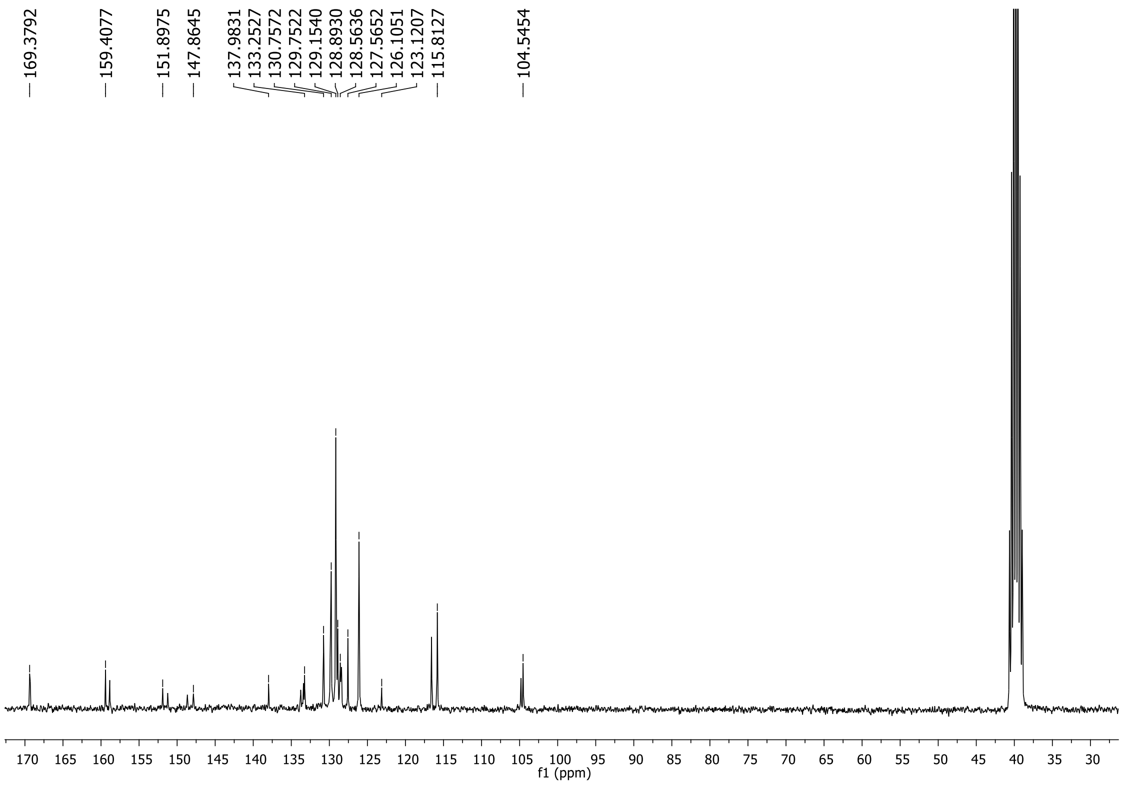

Figure S66: ^{13}C NMR spectra of **3c** (DMSO-d_6, 75 MHz).

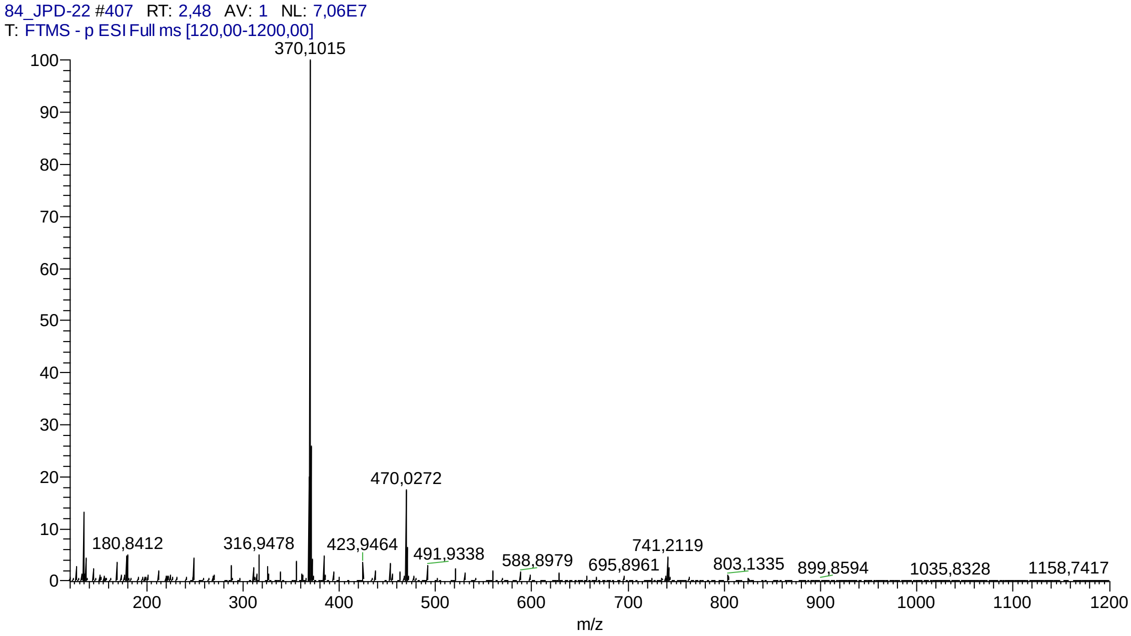

Figure S67: HRMS spectra of **3c**.

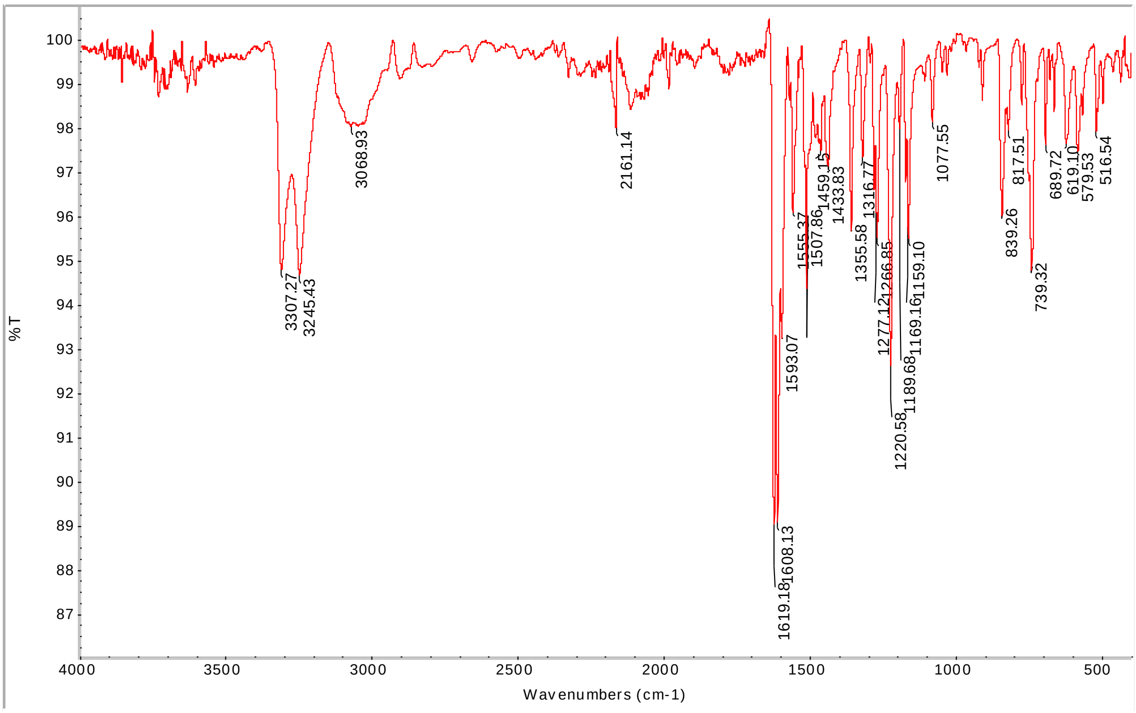

Figure S68: IR spectra of **3d**.

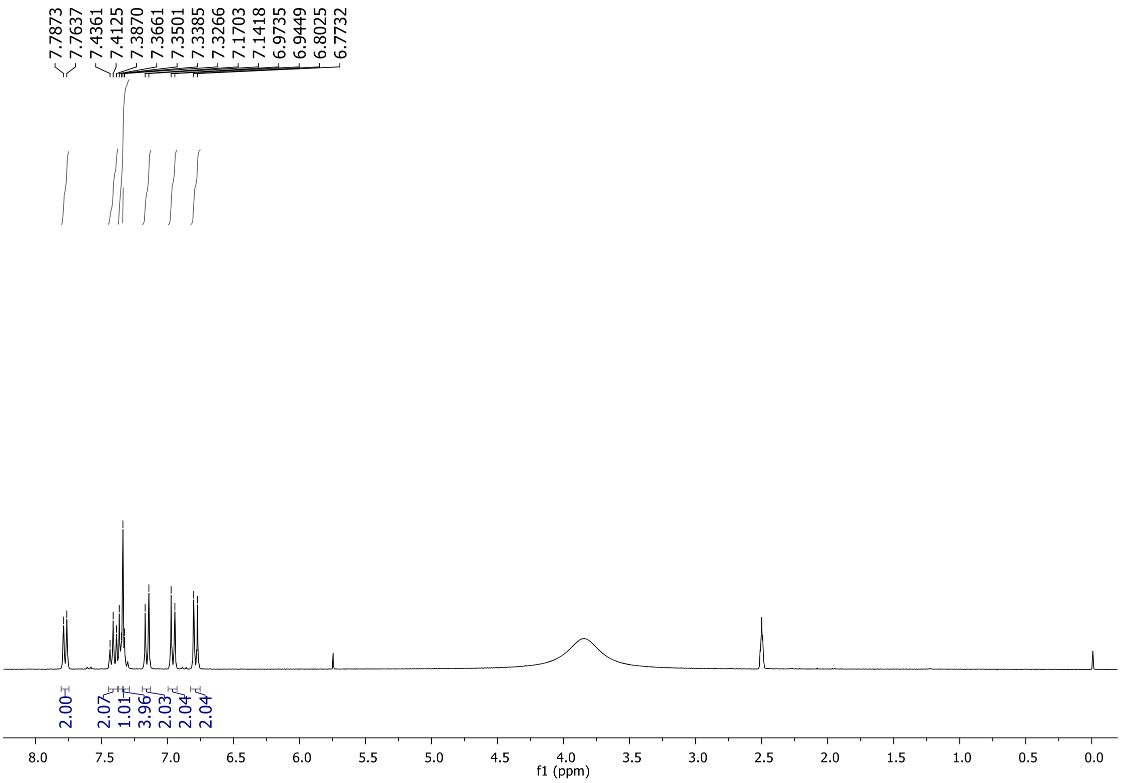

Figure S69: ¹H NMR spectra of **3d** (DMSO-d_6, 300 MHz).

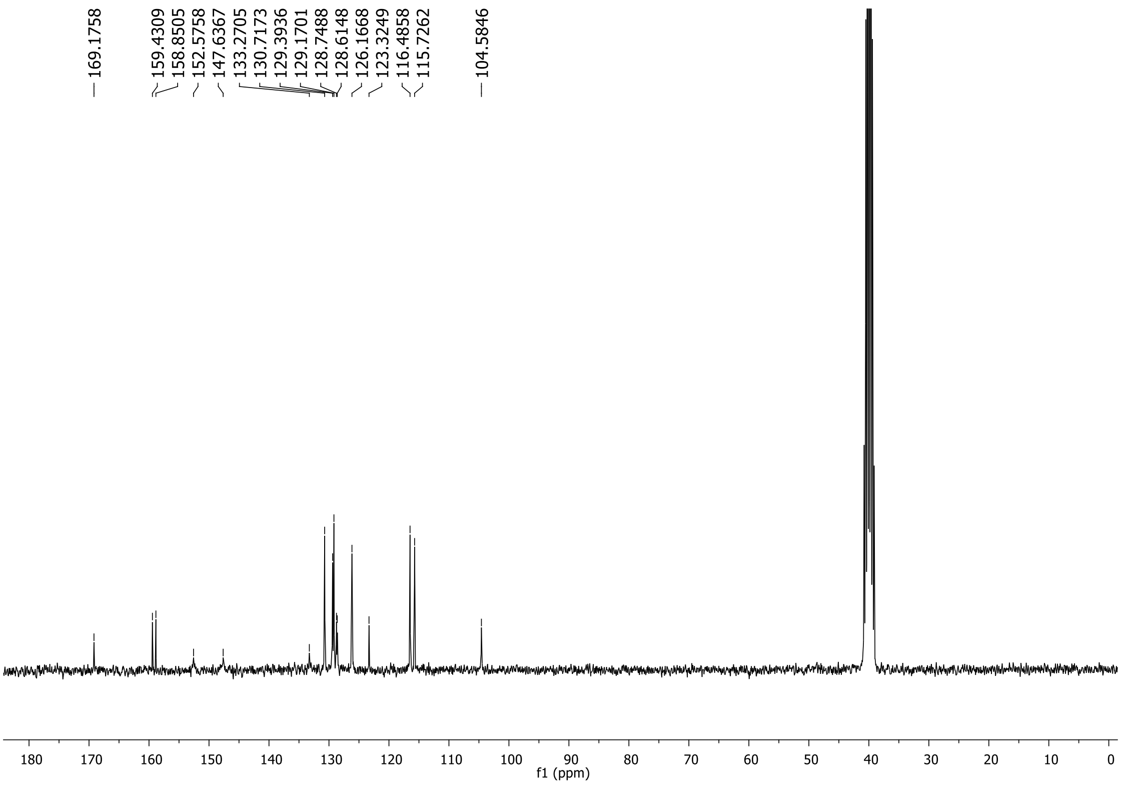

Figure S70: ^{13}C NMR spectra of **3d** (DMSO-d_6, 75 MHz).

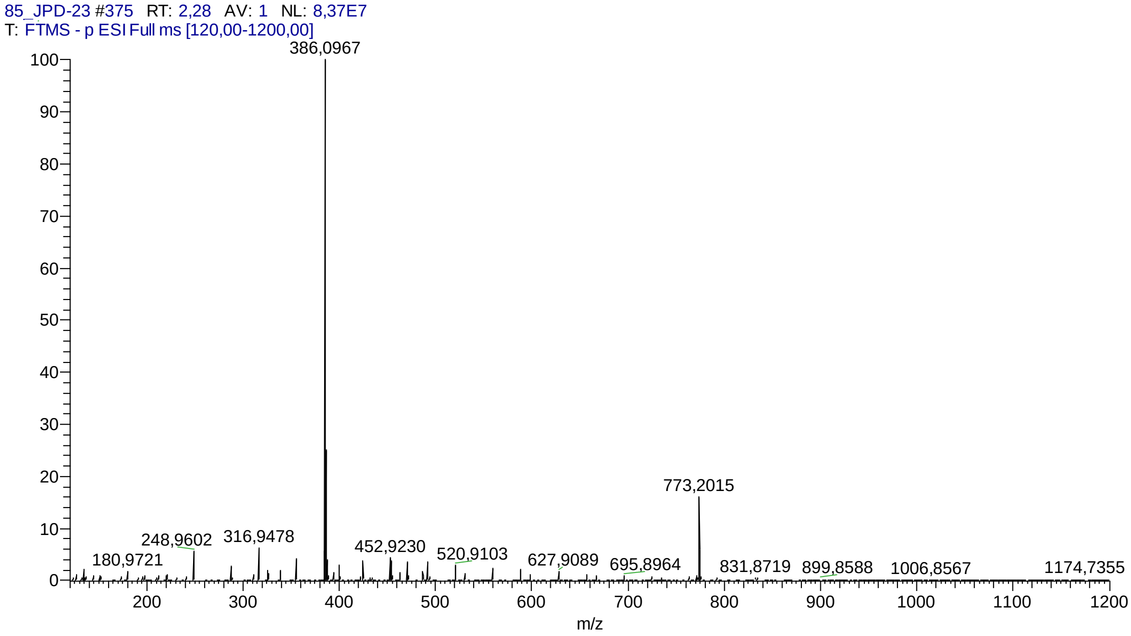

Figure S71: HRMS spectra of **3d**.

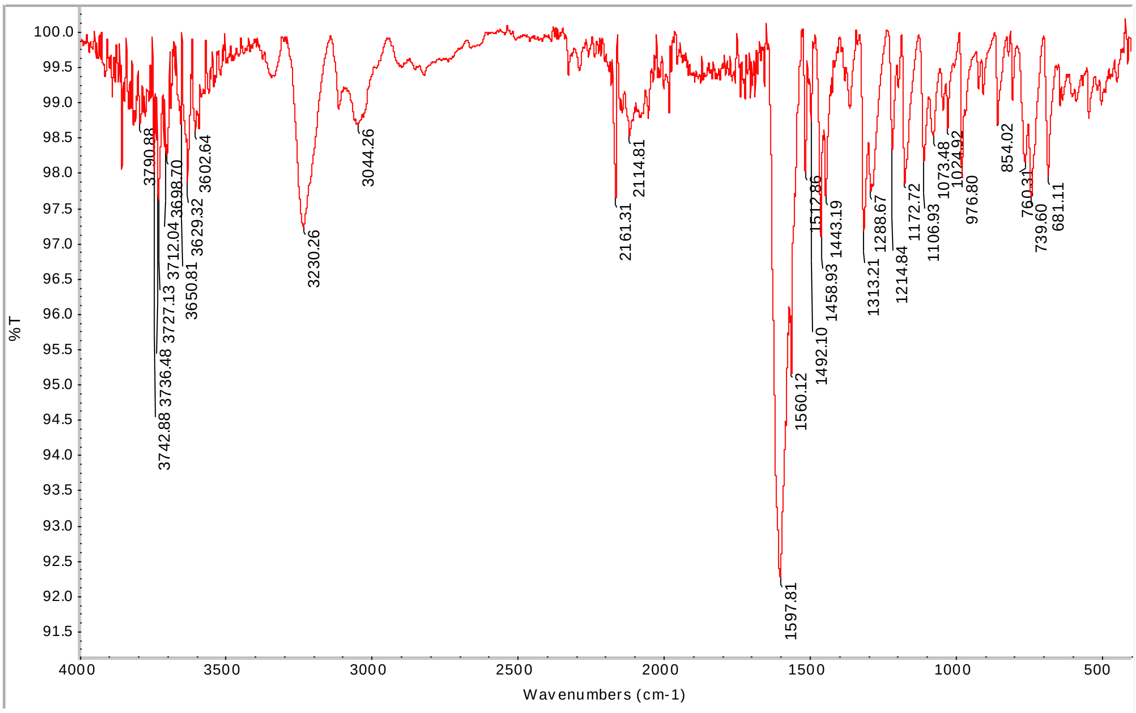

Figure S72: IR spectra of **3e**.

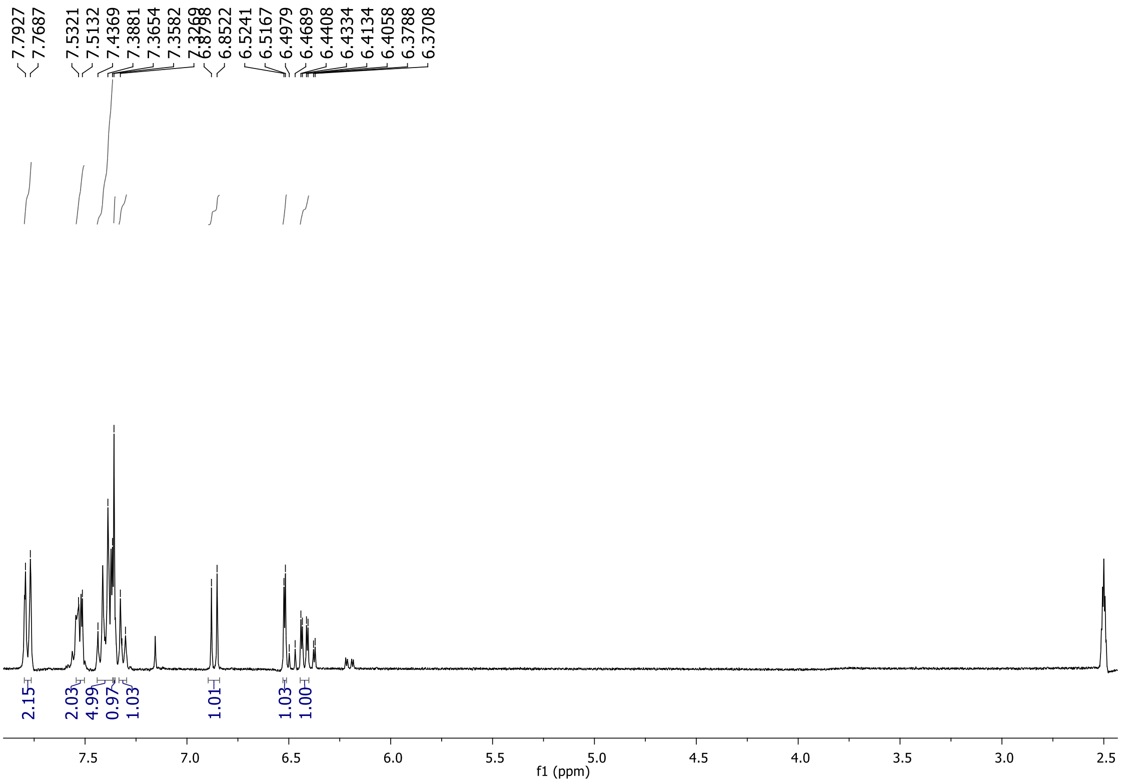

Figure S73: ^{1}H NMR spectra of **3e** (DMSO-d_6, 300 MHz).

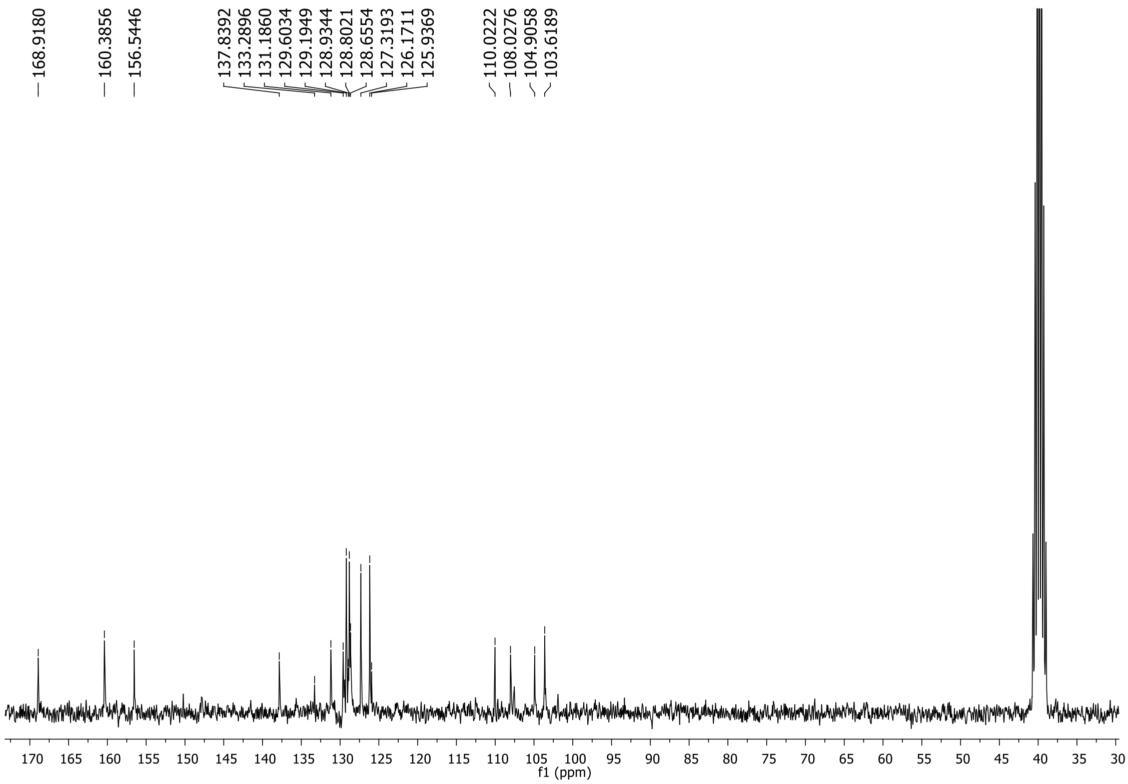

Figure S74: ^{13}C NMR spectra of **3e** (DMSO-d_6, 75 MHz).

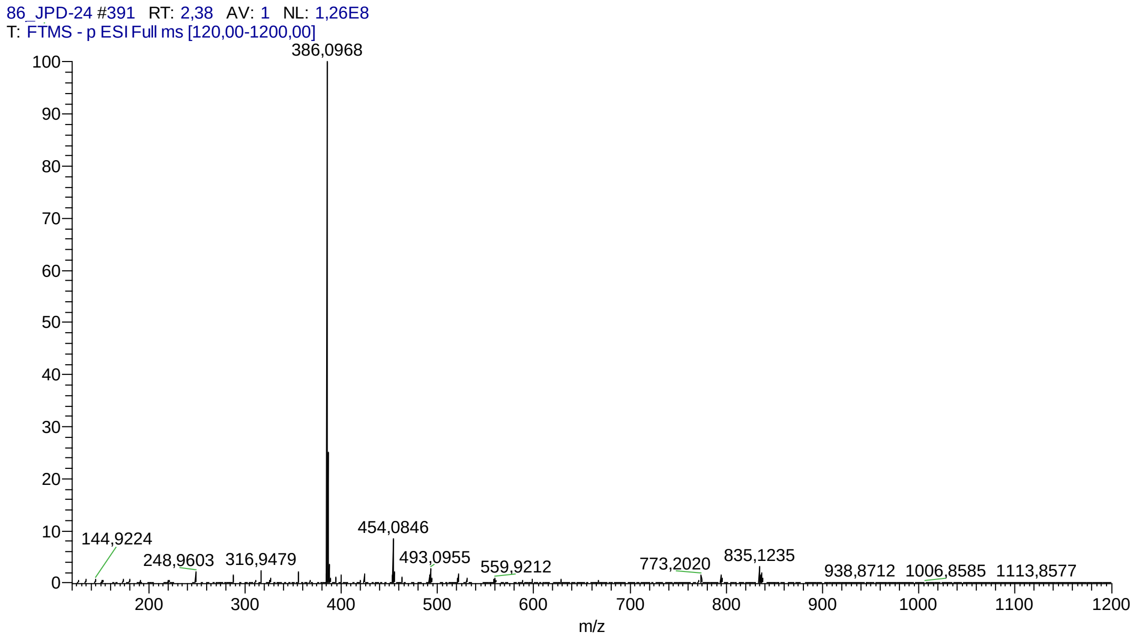

Figure S75: HRMS spectra of **3e**.

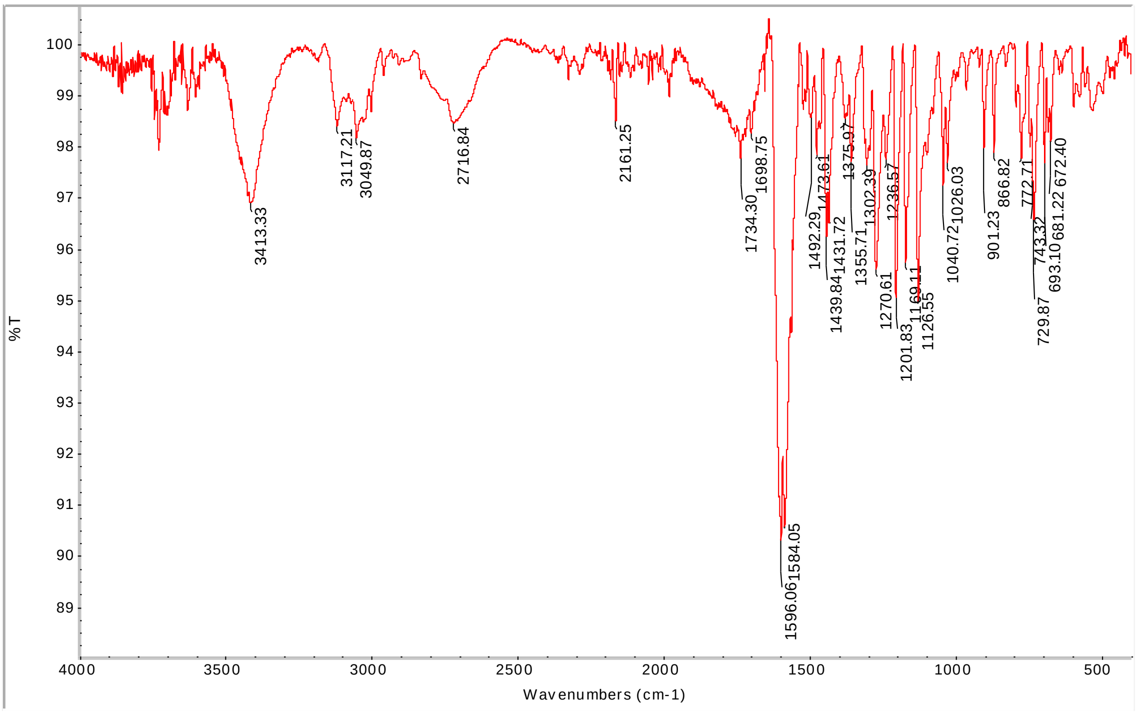

Figure S76: IR spectra of **3f**.

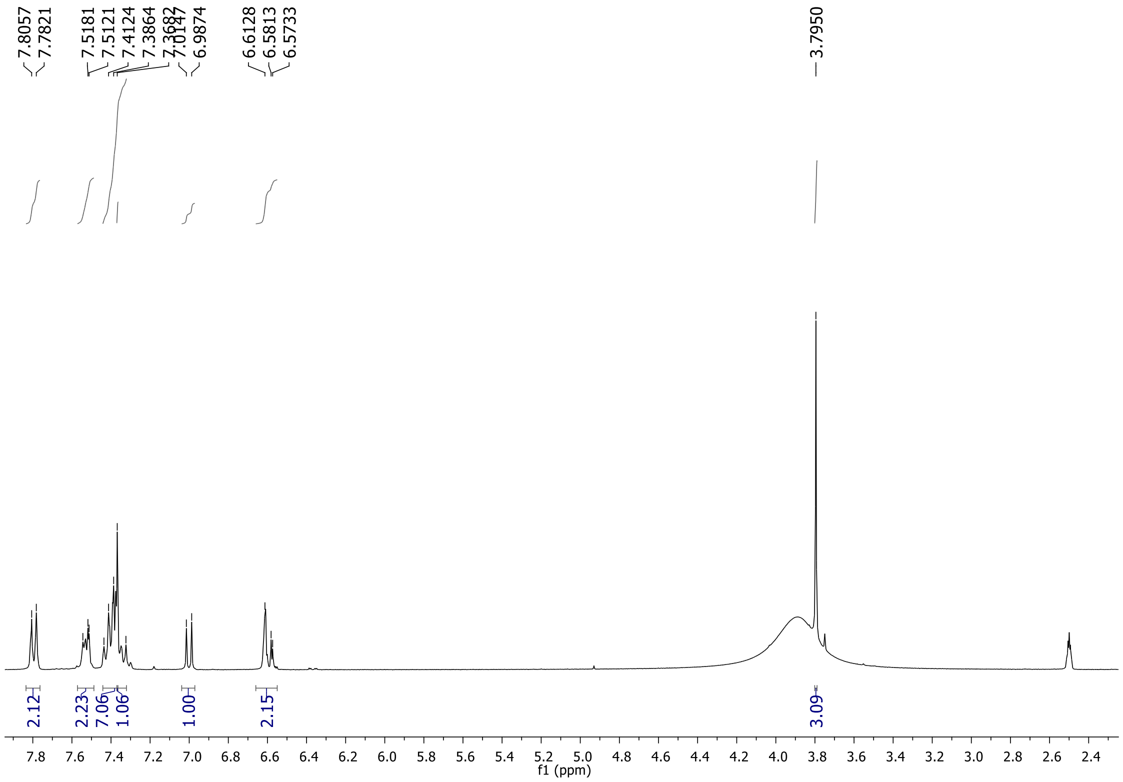

Figure S77: ^{1}H NMR spectra of **3f** (DMSO-d_6, 300 MHz).

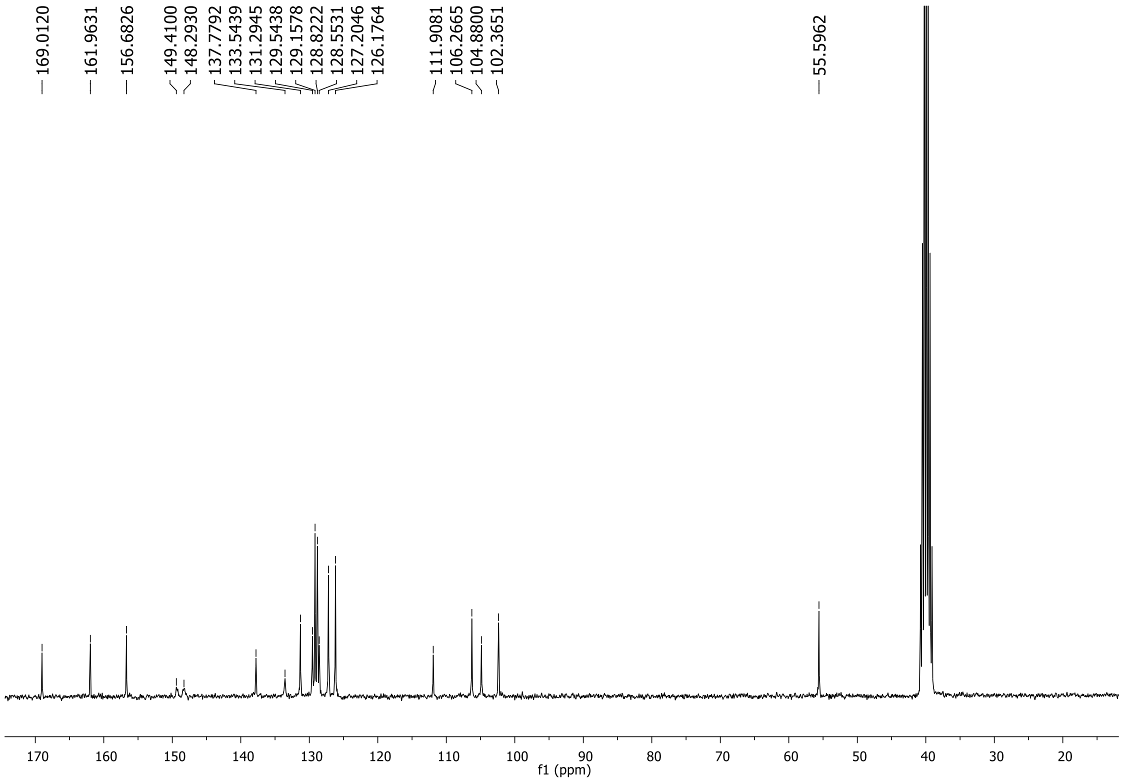

Figure S78: ^{13}C NMR spectra of **3f** (DMSO-d_6, 75 MHz).

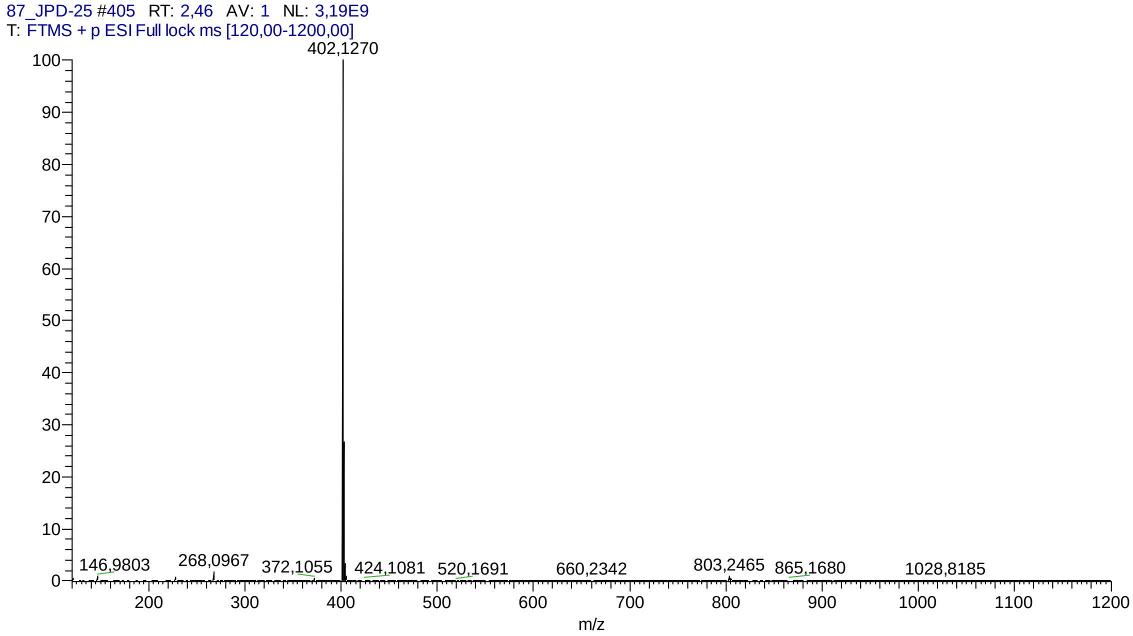

Figure S79: HRMS spectra of **3f**.

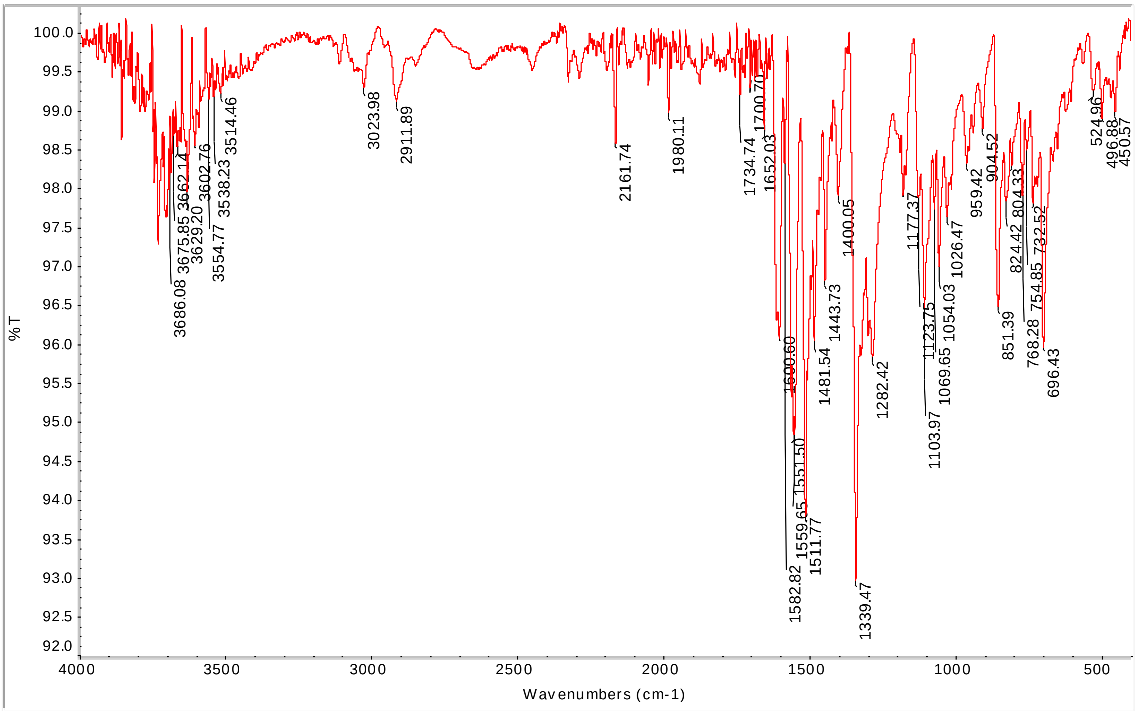

Figure S80: IR spectra of **3g**.

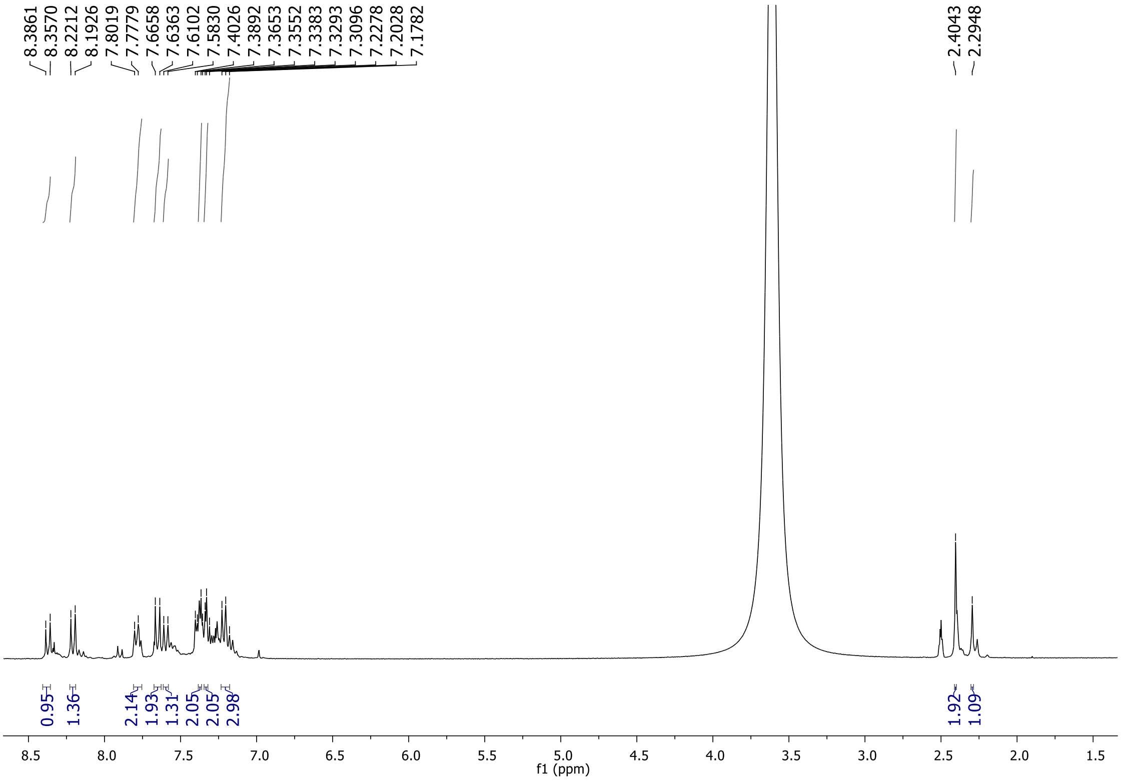

Figure S81: ^{1}H NMR spectra of **3g** (DMSO-d_6, 300 MHz).

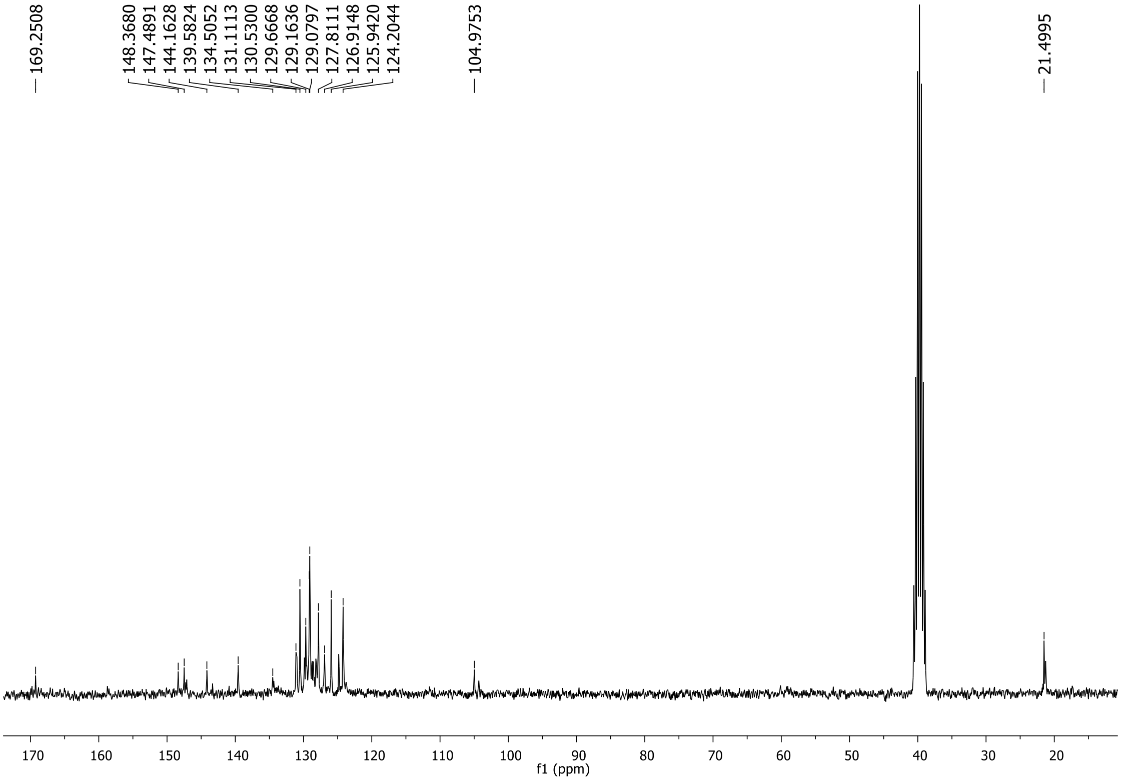

Figure S82: ^{13}C NMR spectra of **3g** (DMSO-*d*$_6$, 75 MHz).

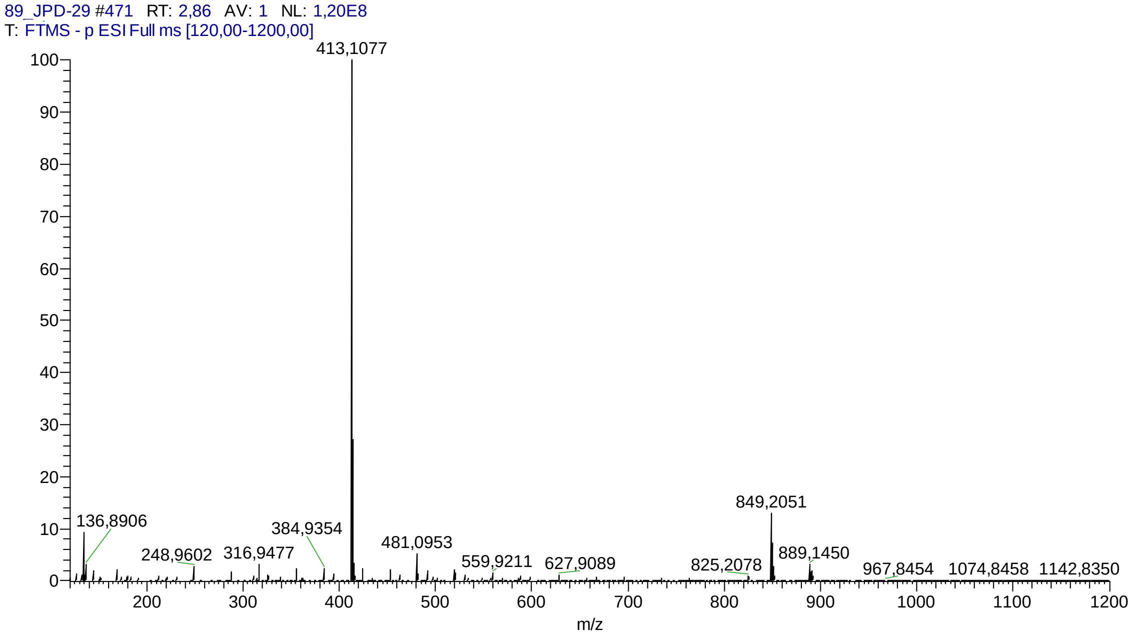

Figure S83: HRMS spectra of **3g**.

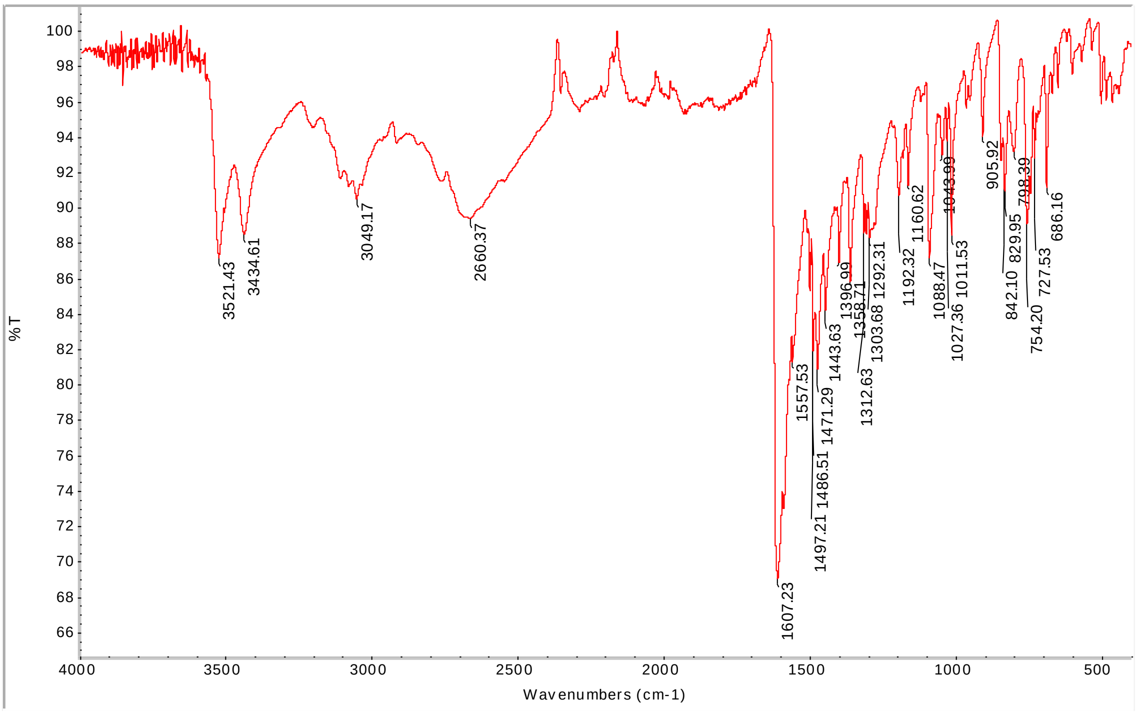

Figure S84: IR spectra of **3h**.

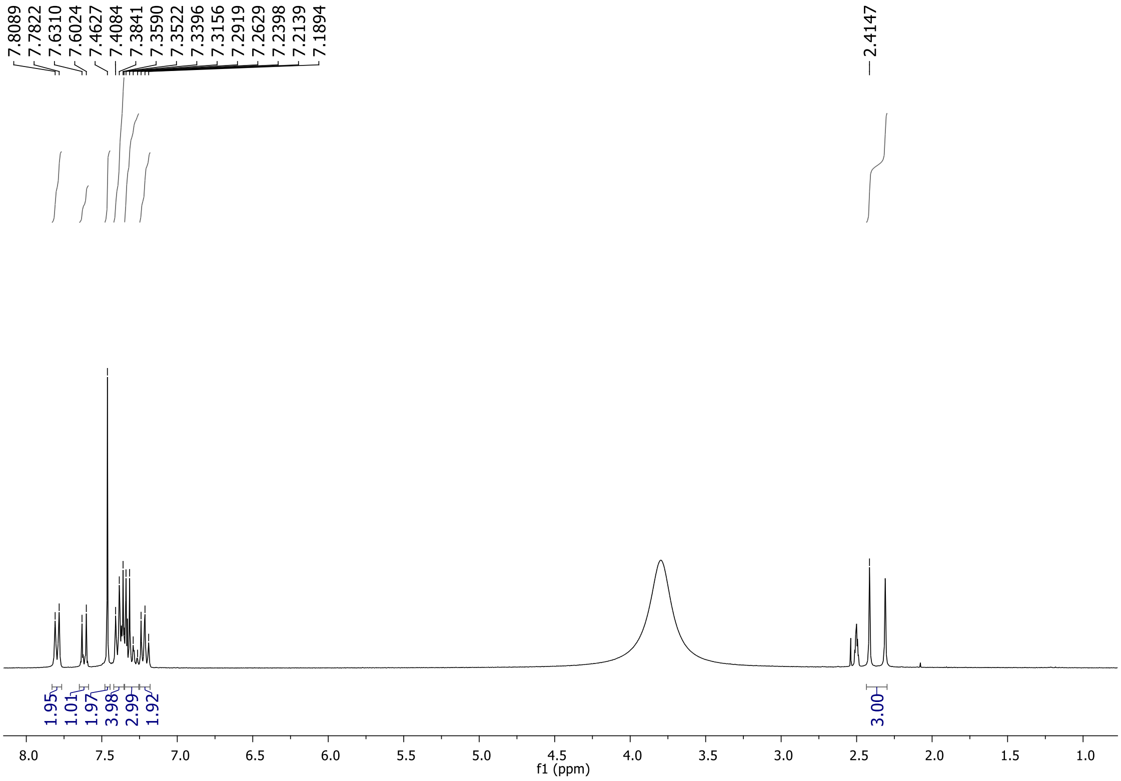

Figure S85: ^{1}H NMR spectra of **3h** (DMSO-d_6, 300 MHz).

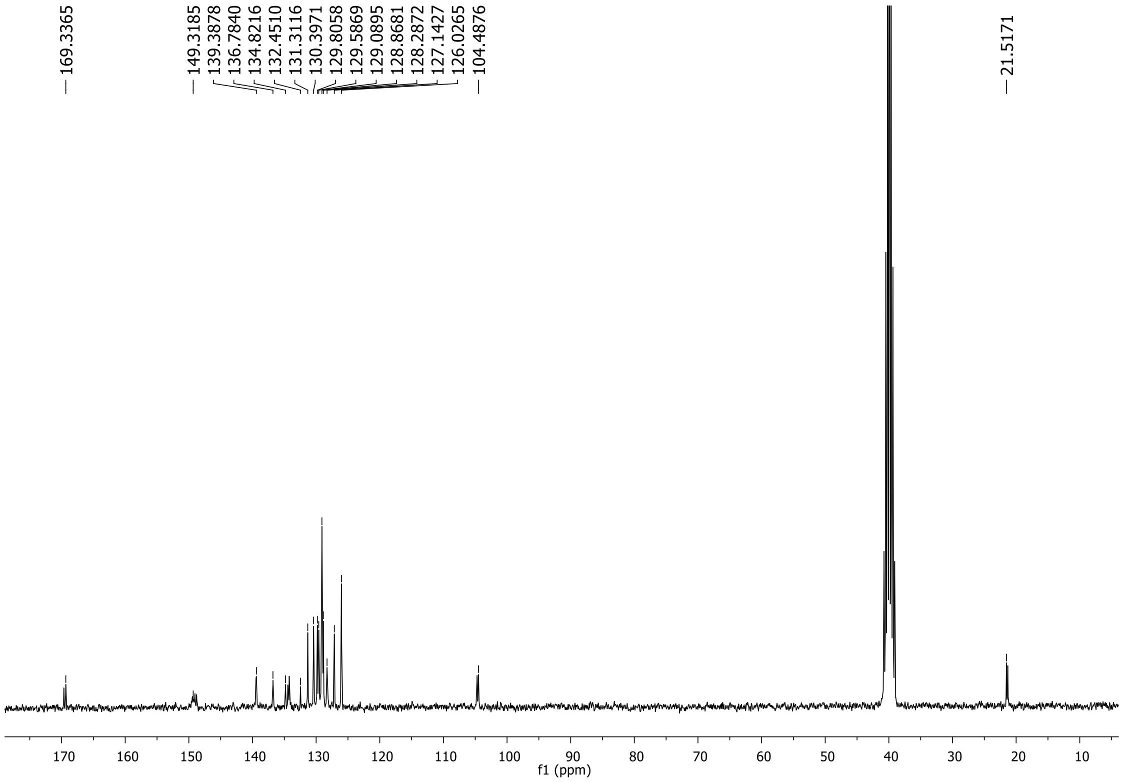

Figure S86: ^{13}C NMR spectra of **3h** (DMSO-d_6, 75 MHz).

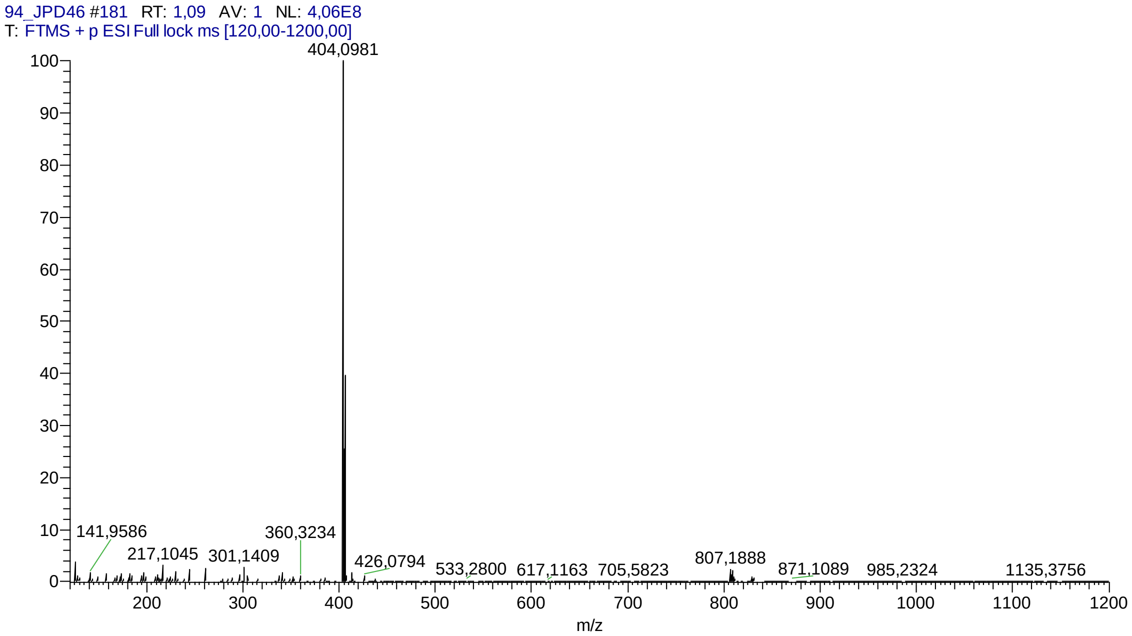

Figure S87: HRMS spectra of **3h**.

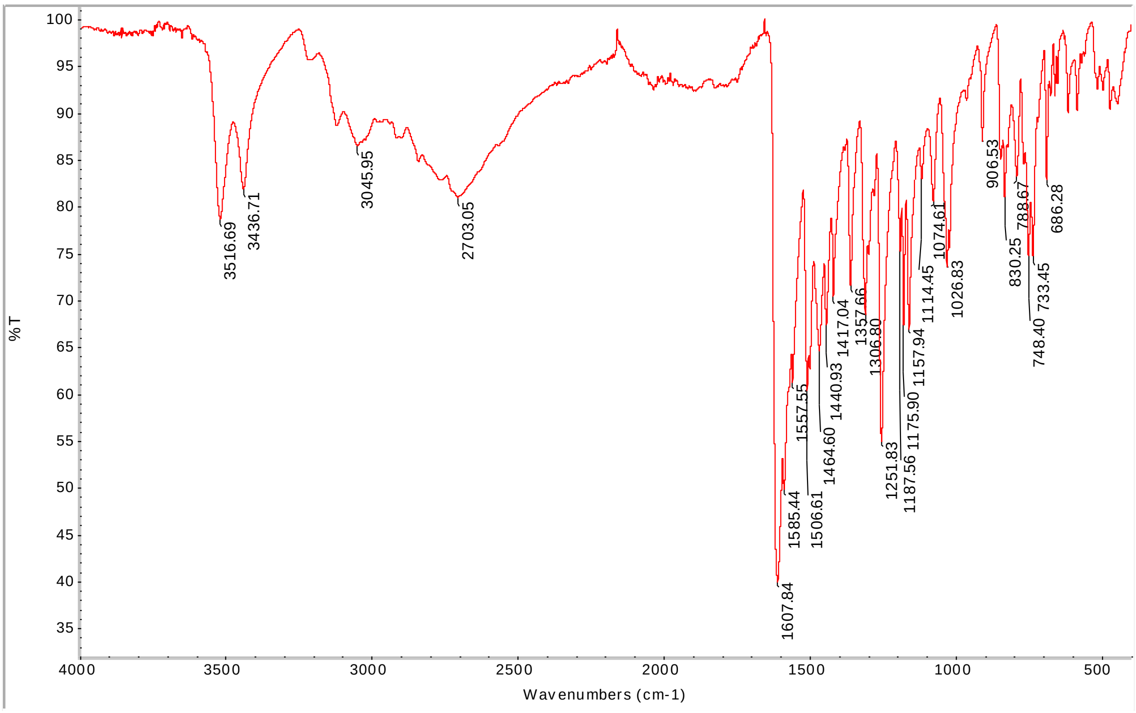

Figure S88: IR spectra of **3i**.

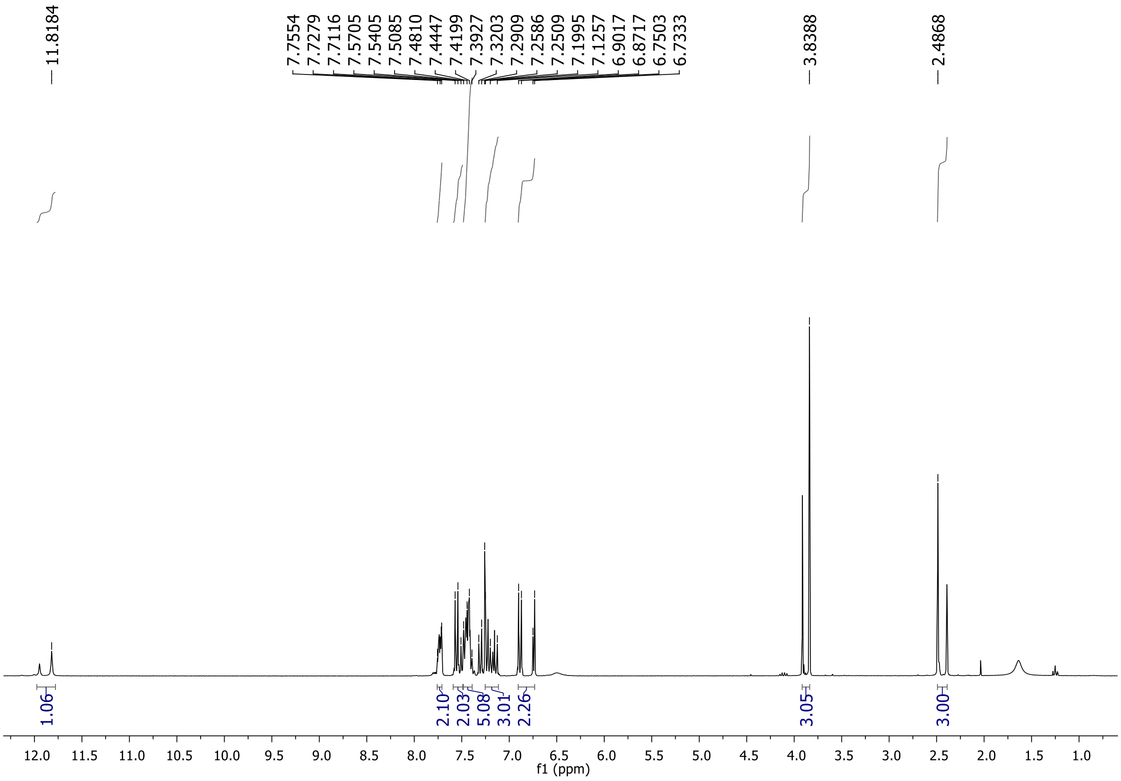

Figure S89: ^{1}H NMR spectra of **3i** ($CDCl_3$, 300 MHz).

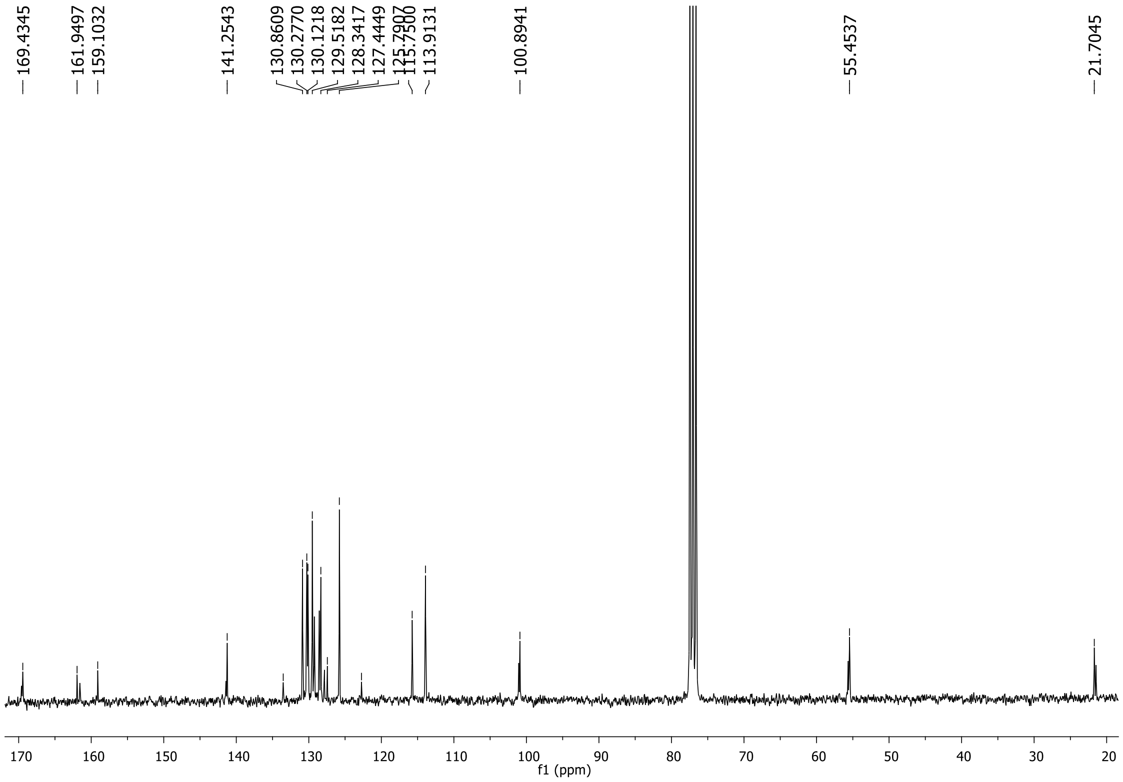

Figure S90: ^{13}C NMR spectra of **3i** ($CDCl_3$, 75 MHz).

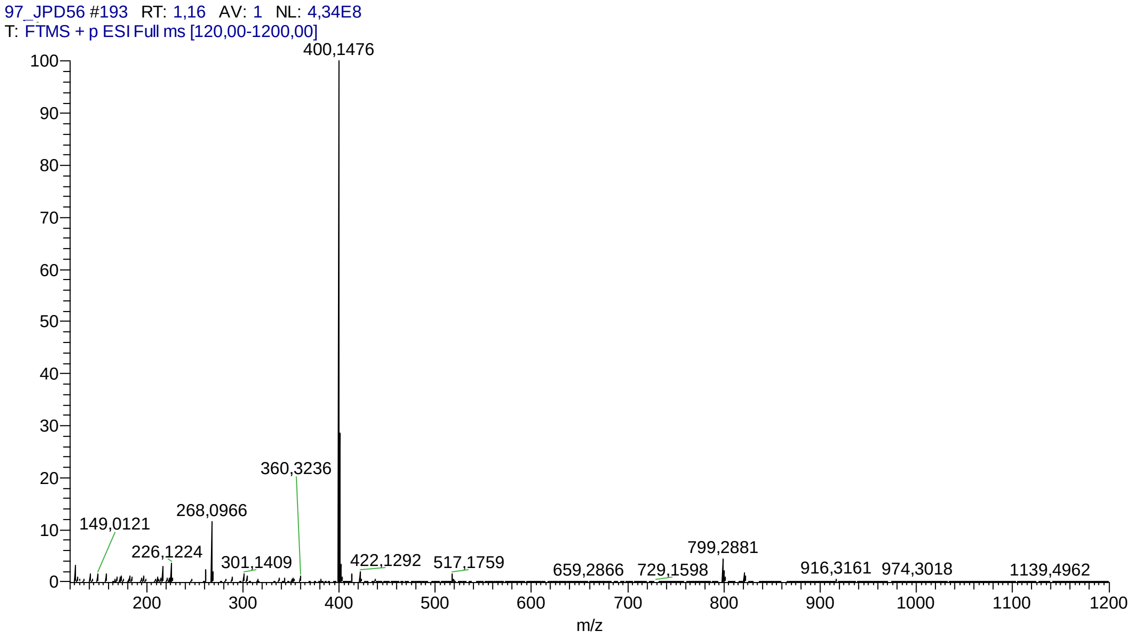

Figure S91: HRMS spectra of **3i**.

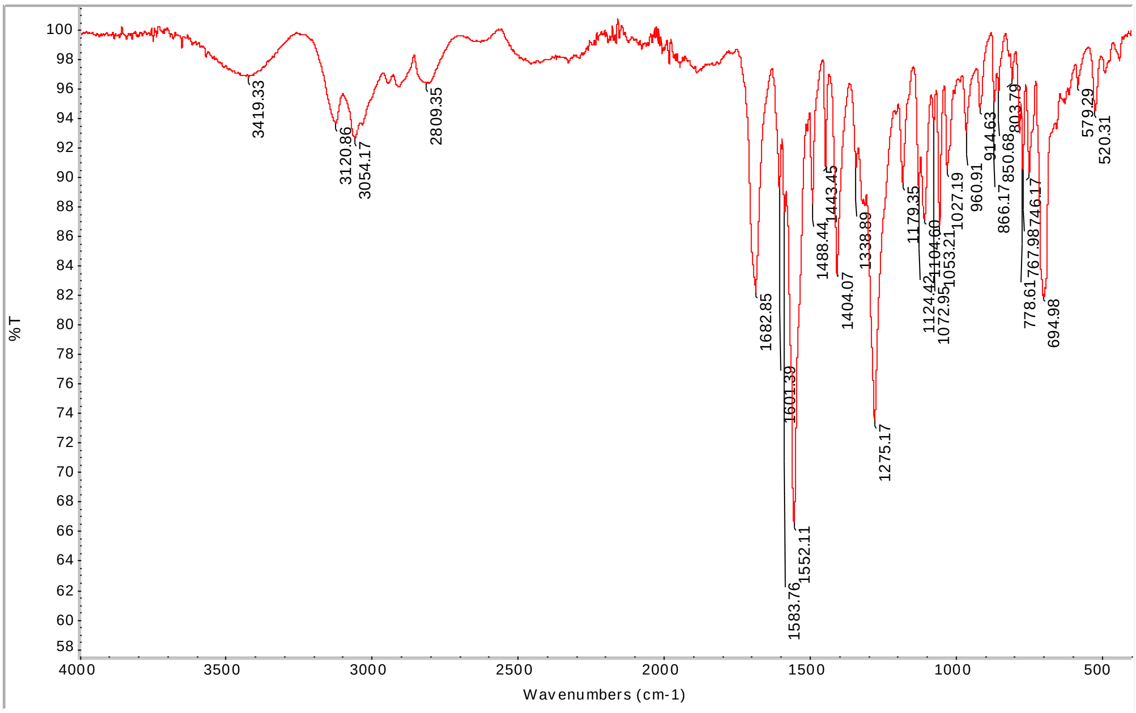

Figure S92: IR spectra of **3i**.

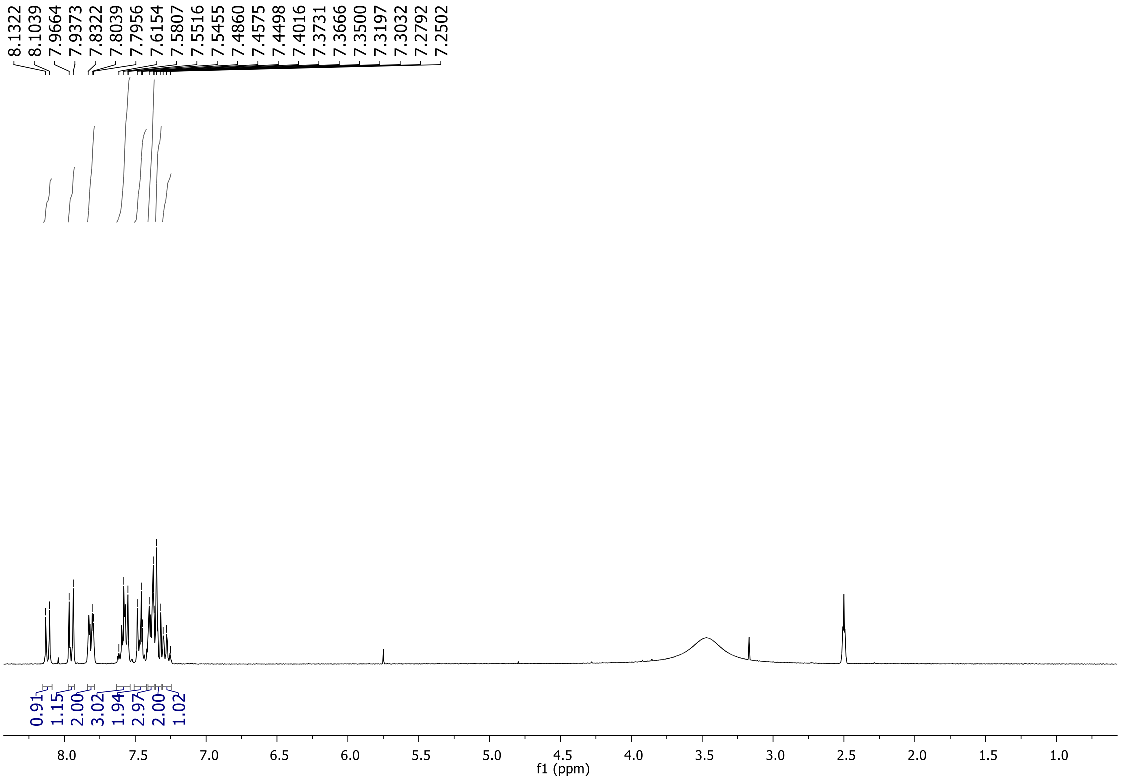

Figure S93: ^{1}H NMR spectra of **3j** (DMSO-d_6, 300 MHz).

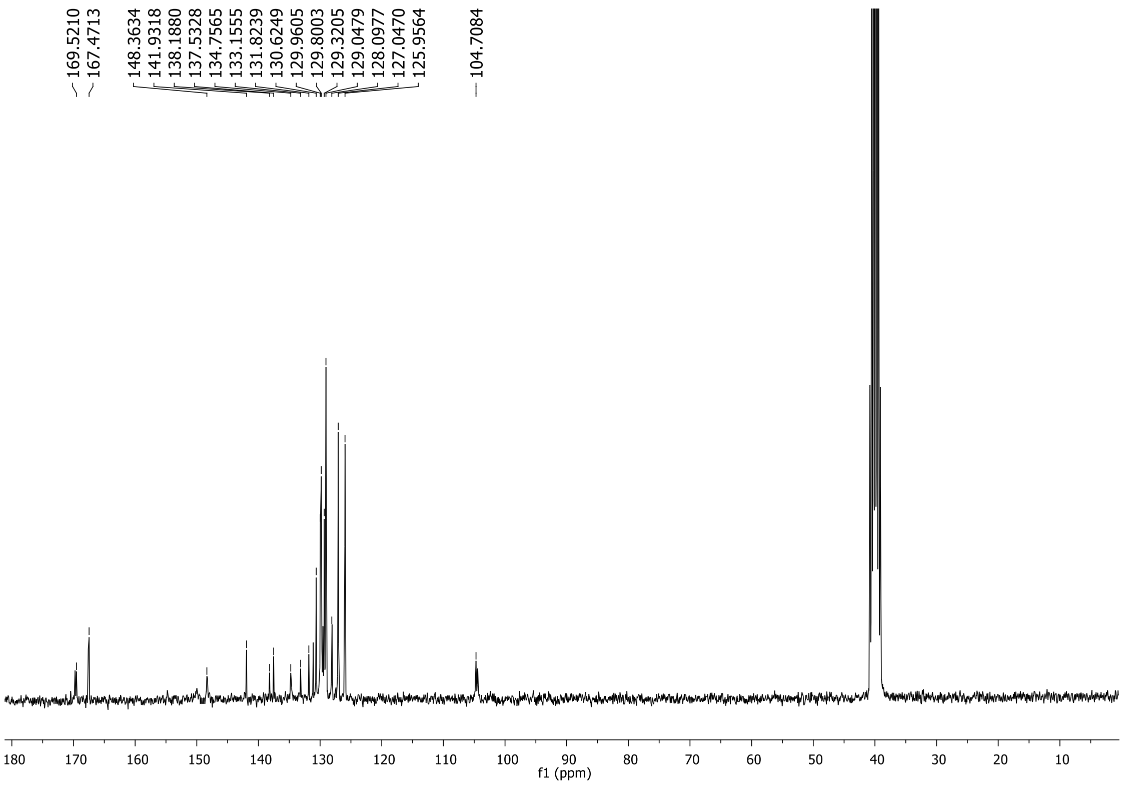

Figure S94: ^{13}C NMR spectra of **3j** (DMSO-d_6, 75 MHz).

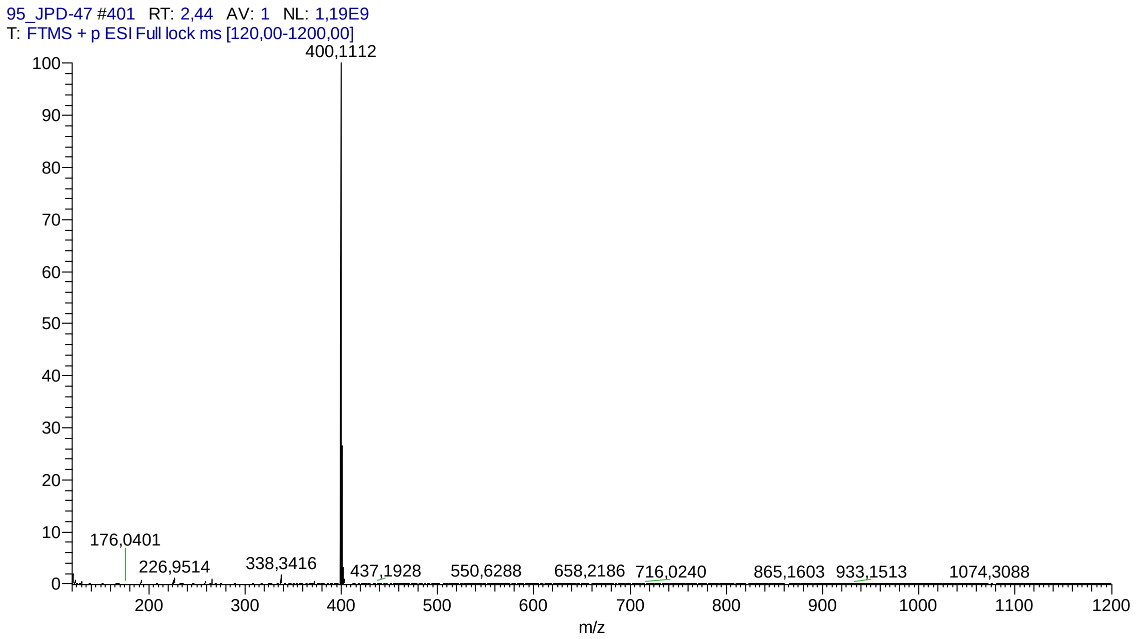

Figure S95: HRMS spectra of **3j**.
